# Broad spectrum structure discovery in large-scale higher-order networks

**DOI:** 10.1038/s41467-026-71903-0

**Published:** 2026-04-27

**Authors:** John Hood, Caterina De Bacco, Aaron Schein

**Affiliations:** 1 Department of Statistics, University of Chicago, Chicago, IL USA; 2 Faculty of Electrical Engineering, Mathematics and Computer Science, Delft University of Technology, Delft, The Netherlands

**Keywords:** Complex networks, Statistical physics

## Abstract

Complex systems are often driven by higher-order interactions among multiple units, naturally represented as hypergraphs. Understanding dependency structures within these hypergraphs is crucial for understanding and predicting the behavior of complex systems but is made challenging by their combinatorial complexity and computational demands. In this paper, we introduce a class of probabilistic models that efficiently represents and discovers a broad spectrum of mesoscale structure in large-scale hypergraphs. The key insight enabling this approach is to treat classes of similar units as themselves nodes in a latent hypergraph. By modeling observed node interactions through latent interactions among classes using low-rank representations, our approach tractably captures rich structural patterns while ensuring model identifiability. This allows for direct interpretation of distinct node- and class-level structures. Empirically, our model improves link prediction over state-of-the-art methods and discovers interpretable structures in diverse real-world systems, including pharmacological and social networks, advancing our ability to incorporate large-scale higher-order data into the scientific process.

## Abstract (web summary)

Identifying higher-order structure that governs how units interact is crucial for modeling complex systems. Here, authors develop a class of scalable probabilistic models that efficiently extract a spectrum of assortative, core-periphery and disassortative structure from higher-order network data.

## Introduction

Complex systems—social, biological, or technological, among other types—are often driven by higher-order interactions among potentially many nodes^1^. Such systems can be modeled as hypergraphs, which extend the traditional notion of graphs or networks from dyadic or pairwise interactions to those of higher order.

Like traditional networks, real-world hypergraphs exhibit mesoscale structure^2,3^—i.e., patterns of interaction among groups of nodes. Broadly, modeling such a structure reduces to clustering nodes into groups and characterizing how those groups interact, if at all. In doing so, one reduces the conceptual complexity of the system and the dimensionality of the data, potentially revealing real functional components and underlying mechanisms that drive observed interactions^4^.

Mesoscale structure comes in many different forms, not all of which are tractably modeled. Perhaps the most commonly modeled structure is assortative structure, wherein nodes form communities and interact mostly with other similar nodes within the same community. Recent work develops methods for efficient detection of these communities in hypergraph data^5–7^. However, many complex systems also exhibit some degree of disassortative structure, wherein similar nodes form classes, but may not interact within these classes. Within these classes, nodes have similar characteristics, but may (exclusively) interact with nodes in other classes. Predator-prey networks in ecology are one such example, wherein species take one of two classes (predator or prey) and interact mostly with species of the other.

Even in traditional network settings, any degree of disassortativity (interaction between nodes of different classes) is generally difficult to model. This challenge stems from the baseline complexity required to model both how nodes form groups and how these combinations of groups interact. For hypergraphs, this complexity compounds: higher-order interactions among nodes introduce higher-order interactions within and between groups, resulting in a combinatoric explosion in the number of model parameters that makes estimation impossible. Recent work contends with this by developing models that surface highly restricted forms of disassortativity in hypergraphs, such as structure that can be modeled by a Bethe approximation^7^, or core-periphery structure wherein a dense core of tightly connected nodes can be distinguished from a sparse, loosely connected periphery of nodes^8,9^.

While prior efforts have analyzed a wide range of hypergraph settings theoretically^5,10–14^, practitioners are limited to only a few options for modeling mesoscale structure in hypergraphs tractably, each of which has its own drawbacks. One approach restricts analysis to assortative (or similarly constrained) structure, risking mischaracterization of a non-assortative system. Another limits the data to a moderate number of small-order interactions (e.g., three- or four-way) to enable the application of one of the more flexible existing approaches, which scale poorly to large, high-order hypergraphs. A third strategy reduces higher-order data to pairwise interactions, potentially discarding crucial structural information through data preprocessing steps^6,15,16^. Collectively, these limitations significantly hamper researchers’ ability to leverage large-scale data to draw reliable conclusions regarding the complex systems they study.

Motivated by these challenges, this paper introduces a family of probabilistic generative models to tractably capture a wide range of mesoscale structures underlying large-scale hypergraphs. This family encompasses several existing models and spans an omniassortative continuum of structural patterns, ranging from strictly assortative to highly disassortative. Where along this spectrum a given model falls is dictated by its parameter values.

**Fig. 1:**
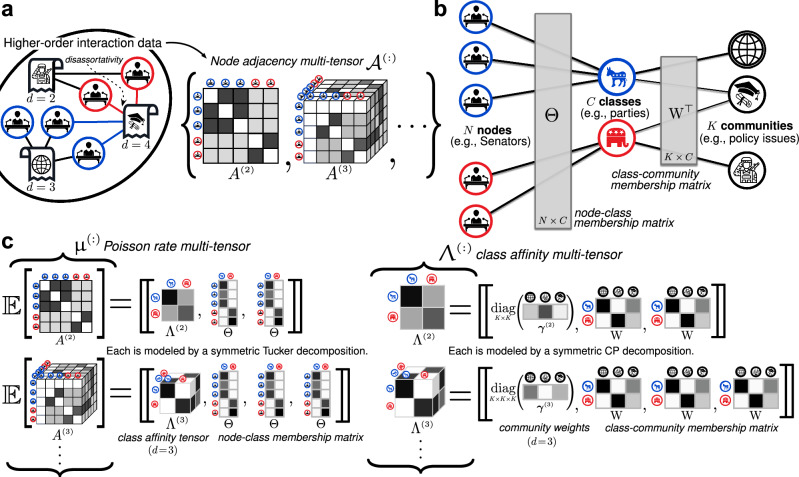
Omni-Hype-SMT models a range of mesoscale structures by assigning nodes to classes and classes to communities. A schematic illustration detailing how Omni-Hype-SMT models a diverse range of mesoscale structures. **a** Hypergraph data encodes higher-order interactions among entities, such as bill co-sponsorship (left). Each hyperedge captures a multi-way relationship among nodes. These interactions are represented as a multi-order adjacency tensor ${{{\mathcal{A}}}}^{(:)}$ (right), where *A*^(*d*)^ denotes the order-*d* adjacency tensor capturing *d*-way interactions. Such a formulation enables the modeling of complex, non-pairwise relational structures. **b** Two parameters, Θ and W, govern interactions between nodes (e.g., Senators). The node-class membership matrix Θ soft clusters similar nodes to classes (e.g., political parties), while the class-community membership matrix W models interactions between classes through communities (e.g., policy issues). Together, the matrix ΘW captures assortative and disassortative activity between nodes through interactions within and between classes. **c** The multi-tensor *μ*^(:)^ (left) models the observed higher-order adjacency tensors ${{{\mathcal{A}}}}^{(:)}$ via symmetric low-rank tensor decomposition using a class affinity tensor *Λ*^(*d*)^ and a node-class membership matrix Θ, shared over orders *d*. The class affinity multi-tensor Λ^(:)^ (right) is further decomposed into community-order rates ${\gamma }_{k}^{(d)}$ and the symmetric outer products of the columns of the class-community membership matrix W, enabling interpretable modeling of multi-way class interactions across different orders. The icons are taken from^59 -- 61^, and the OmniGraffle^63^ software was used to create these diagrams.

The key idea driving our approach is to jointly cluster nodes into classes and classes into communities. Each clustering is soft, permitting nodes to belong to multiple classes, and classes to multiple communities, as shown in Fig. 1b. The proposed model, which we refer to as Omni-Hype-SMT, avoids the combinatoric explosion associated with modeling all combinations of inter-class interaction by dictating that classes interact exclusively within communities. By clustering classes into communities, Omni-Hype-SMT allows for disassortative interactions between nodes. Since classes are made up of similar nodes, interactions between different classes yield disassortative interactions between nodes of those classes. It is through this framework that the proposed model captures rich and interpretable latent structure governing higher-order interactions among nodes.

We leverage a principled framework of statistical inference to rigorously identify mesoscale structure in hypergraph data. Our proposed model is defined such that its parameters are provably identifiable from the data, enhancing the reliability and robustness of their interpretation. Beyond providing a rigorous proof of model identifiability, we derive efficient parameter updates by exploiting the probabilistic nature of the proposed model and conditional distributions within. It is this efficiency that enables large-scale, data-driven discovery of mesoscale structure in practice. We demonstrate how this efficiency enables the development of an extremely simple yet scalable algorithm for generating synthetic hypergraphs with tunable mesoscale structure—a capability largely missing from the current literature^5,17^, due to the computational challenges posed by the high-dimensional nature of hypergraphs. Altogether, the analytic and computational tractability of our approach, along with its theoretical guarantees, makes it a principled and effective way to disentangle different types of mesoscale structure underlying large-scale complex systems.

We demonstrate the expressiveness and scalability of Omni-Hype-SMT through extensive experiments on two biological datasets, three human-contact networks, and three political datasets, each of which has a natural hypergraph representation. We find a diverse range of mesoscale structures extending beyond strict assortativity. We prove that a state-of-the-art assortative model^6^ is a specific, restricted instance of the proposed model class. We focus our comparisons here to demonstrate the proposed model’s advanced capabilities. Our flexible modeling approach leads to better performance on downstream tasks, yielding enhanced higher-order link prediction and more interpretable node clustering than existing approaches. Our results reflect an intuitive insight: as the order of interaction increases, appropriately modeling disassortative structure becomes more important. The proposed method is a powerful tool for discovery in higher-order networks consisting of a mix of assortative and disassortative structure. In a case study of higher-order drug combinations among emergency room (ER) patients, we demonstrate how learning diverse types of mesoscale structure provides more nuanced insights into drug classes and their interaction patterns than previously available. We show how Omni-Hype-SMT discovers both classes of similar drugs *and* higher-order interactions between them. For example, pairwise interactions between drugs tend to be between recreational drugs—a class inferred by the model and labeled post hoc via generative AI—such as alcohol and marijuana. On the other hand, among patients with many drugs in their system, if one is a cardiovascular medication such as lisinopril or metoprolol, the others are often psychotropic medications (quetiapine, clonazepam) or opioid analgesics (oxycodone, hydrocodone, fentanyl). When mixed, these classes of drugs that are often prescribed for medical benefit increase in potency. These insights may inform clinical risk assessment and the design of safer polypharmacy protocols by revealing latent, higher-order interaction patterns that are obscured in pairwise analyses. Together, these findings highlight the importance of modeling a full spectrum of mesoscale structure in uncovering clinically and scientifically meaningful patterns in complex, higher-order data.

## Results

### Motivating departure from strict assortativity: two case studies

We start by showing how existing probabilistic approaches, which are based on assumptions of strict assortativity, provide sub-optimal representations of the data when fit to hypergraphs exhibiting realistic degrees of disassortativity. We analyze two datasets, each exhibiting a different type of mesoscale structure departing from strict assortativity.

First, we draw upon a dataset of human-contact interactions in a hospital, where nodes are either staff (i.e., doctors, nurses, or administrative assistants) or patients^18^. In this dataset, hyperedges describe proximity interactions as measured by wearable Bluetooth devices. We create a semi-synthetic version of the data by removing hyperedges that do not contain at least one patient. Thus, all interactions are either exclusively between patients or those consisting of at least one patient and one staff. We then fit two models: 1) the state-of-the-art strictly assortative model^6^ mentioned above, and 2) our omniassortative model.

**Fig. 2:**
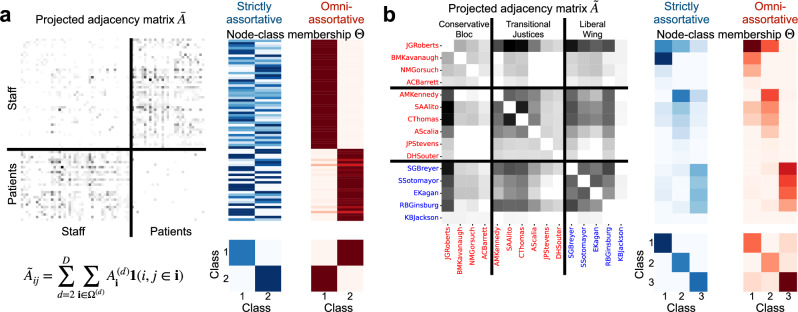
Omni-Hype-SMT recovers core-periphery structure among US Supreme Court justices and disassortative structure in hospital proximity data. In each setting, the projected adjacency matrix of the hypergraph data is visualized next to the inferred node-class membership and class affinity matrices. The omniassortative model’s class affinity matrix has non-zero intensity on its off-diagonal, representing possible interactions between classes, while the strictly assortative matrices are zero on the off-diagonal. **a** In the hospital setting, the node-class membership matrix cleanly separates patients from staff for the omniassortative model (cosine similarity with input node attributes: 0.76 ± 0.003, averaged over random initializations) but not the strictly assortative one (cosine similarity with input node attributes: 0.46 ± 0.002); we normalize each node’s membership vector to emphasize this difference. For the omniassortative model, the class affinity matrix is strongly off-diagonal, suggesting that interactions among patients and staff are highly disassortative. **b** In the Supreme Court Justice setting, we color-code the justices according to the party of the nominating President, and see that both models cleanly separate three blocks of justices, known loosely as the liberals, conservatives, and transitional justices. The omniassortative model’s node-class membership matrix is sharper, with less mixed-membership, and it explains the crossover in voting patterns across blocks by inferring core-periphery structure, where the class affinity matrix is strongly diagonal but has a non-negligible off-diagonal.

At a high-level, both models can be understood as soft-clustering *N* nodes into *C* latent classes, represented by an *N* × *C* node-class membership matrix. We visualize the matrices learned by each of the two models in the bottom panel of Fig. 2a. The strictly assortative model (left column) requires that nodes of different classes do not interact and fails to recover the underlying staff-versus-patient block structure. On the other hand, the omniassortative model (right column) cleanly partitions the nodes into patients and staff and appropriately models interactions between the groups. In addition to providing a more interpretable group-level description of the data, the omniassortative model predicts held-out hyperedges better than the strictly assortative one (AUC of 0.91 versus 0.85).

In our second example, we use a dataset of United States Supreme Court cases ranging over the years 2005–2024. The data forms a hypergraph wherein nodes are Supreme Court justices and each hyperedge corresponds to the set of justices who concurred with the majority opinion of a given case. Here, both models recover similar block structure, shown in the top panel of Fig. 2b. However, the classes inferred by the assortative model are less distinguishable, as measured by the entropy ${\mathbb{H}}(\Theta )$ (defined later in this section) of the node-class membership matrix. The omniassortative model, on the other hand, cleanly distinguishes between classes corresponding largely to Democrat- versus Republican-appointed justices. The strictly assortative model assigns the Republican-appointed Justices Roberts, Kennedy, Alito, Thomas, and Scalia partial membership in a class dominated by Democratic appointees, while the omniassortative model does not. The omniassortative model permits interactions between classes—thus, it does not require heavily mixed class membership to explain interactions between Republican-appointed justices who sometimes concur with Democrat-appointed ones. The two bottom-most panels in Fig. 2b depict affinities between classes, where we note that the strictly assortative affinity matrix (blue) is constrained to be diagonal.

These examples represent two distinctly different departures from strict assortativity. The first shows a highly disassortative structure, where patients and staff primarily interact across classes, instead of within them. The second example shows a core-periphery structure, where justices primarily agree with other justices of similar ideology (i.e., conservatives agree with conservatives) but also agree with justices of opposing ideologies (such as in unanimous rulings), albeit less often. These simple examples motivate our modeling approach, described next.

### Omni-Hype-SMT: an omniassortative model of hypergraphs from symmetric multi-tensors

Here, we introduce Omni-Hype-SMT, a probabilistic model that can infer a flexible array of mesoscale structure from large-scale hypergraph data. In its specification, the model blends structural patterns from stochastic block modeling approaches for traditional networks^19–21^ with multilinear tensor decomposition approaches for multiplex networks^22–25^. In particular, nodes have overlapping membership in a set of classes, which themselves then exhibit higher-order interactions. Class-level interaction rates are governed by an affinity multi-tensor Λ^(:)^, settings of which determine the type of mesoscale structure present in the hypergraph. Λ^(:)^ is very high-dimensional—we impose a certain low-rank factorization, which makes the model both identifiable, enabling the recovery of meaningful latent structure, and tractable to estimate.

Before describing *Λ*^(:)^ in detail, we define hypergraphs mathematically using tensor notation. Formally, a hypergraph (with hyperedges of arbitrary size) can be represented as a multi-tensor—i.e., a series of tensors—${{{\mathcal{A}}}}^{(:)}:=\{{A}^{(2)},\ldots,{A}^{(D)}\}$—where the *d*^th^ adjacency tensor *A*^(*d*)^ stores all observed *d*-order interactions, with *d* = 2 corresponding to pairwise interactions, and *d* = *D* to the maximum observed order.

Each adjacency tensor ${A}^{(d)}\in {{\mathbb{N}}}_{0}^{N\times \cdots \times N}$ is a *d*-way symmetric count tensor with entries ${A}_{{{\bf{i}}}}^{(d)}:={A}_{{i}_{1}\ldots {i}_{d}}^{(d)}$, each of which denotes the number of observed interactions between a particular set of *d* nodes, where the multi-index **i** ≔ (*i*_1_, …, *i*_*d*_) ∈ *Ω*^(*d*)^, takes values in the set of all possible interactions between *d* nodes. For instance, for a legislative bills dataset, ${A}_{{{\bf{i}}}}^{(d)}$ could be the number of bills that a given group of legislators *i*_1_,…, *i*_*d*_ co-sponsored. We explicitly model the entries *i*_1_ < ⋯ < *i*_*d*_ and define the remaining entries by symmetry, such that $$| {\Omega }^{(d)}| \,=\,\left(\begin{array}{c}N\\ d\end{array}\right)$$ and the remaining (e.g., diagonal) entries are undefined. Our model applies to hypergraphs with arbitrary hyperedge sizes. It can be applied to heterogeneous datasets that span a wide distribution of hyperedge sizes, as well as to homogenous datasets, including pairwise networks, where *d* = 2 for all (hyper)edges.

The proposed model builds on previous work^5–7^ in assuming that the number of observed higher-order interactions between different sets of nodes are conditionally Poisson-distributed—i.e.:

1 $${{{\mathbb{P}}}}({{{\mathcal{A}}}}^{(:)}\,| \,{\mu }^{(:)})={\prod }_{d=2}^{D}{\prod }_{{{\bf{i}}}\in {\Omega }^{(d)}}{\mathrm{Poisson}}\left({A}_{{{\bf{i}}}}^{(d)};\,{{\mu }_{{{\bf{i}}}}^{(d)}}\right),$$

where ${{\mu }_{{{\bf{i}}}}^{(d)}} > 0$ is an interaction rate—i.e., ${\mathbb{E}}[{A}_{{{\bf{i}}}}^{(d)}]\,=\,{{\mu }_{{{\bf{i}}}}^{(d)}}$. In practice, these rates are unknown, and we aim to estimate them. The number of such rates is combinatoric, one for each possible higher-order interaction, and typically only a small fraction of interactions are observed in practice (i.e., ${{{\mathcal{A}}}}^{(:)}$ is sparse), making estimation difficult.

To handle the prohibitive dimensionality of *μ*^(:)^, we assume it admits the following low-rank factorization:

2 $${{\mu }_{{{\bf{i}}}}^{(d)}}={\sum }_{{c}_{1}=1}^{C}\ldots {\sum }_{{c}_{d}=1}^{C}{\Lambda }_{{c}_{1}\ldots {c}_{d}}^{(d)}{\prod }_{r=1}^{d}{\theta }_{{i}_{r}{c}_{r}}.$$

Beyond reducing dimensionality, this factorization yields a particular interpretation wherein ${\theta }_{{i}_{r}{c}_{r}} > 0$ represents the rate at which node *i*_*r*_ acts as a member of class *c*_*r*_ and ${\Lambda }_{{c}_{1}\ldots {c}_{d}}^{(d)} > 0$ represents the rate of *d*-order interactions between the classes *c*_1_, …, *c*_*d*_. We can interpret each *C*-length vector ***θ***_*i*_ ≔ (*θ*_*i*1_, …, *θ*_*iC*_) as representing the overlapping class membership of node *i*, and the set of such vectors as collectively forming the *N* ×* C* node-class membership matrix Θ for a fixed number of classes *C*.

Analogous to the data, the rates ${\Lambda }_{{c}_{1}\ldots {c}_{d}}^{(d)}$ collectively form a multi-tensor Λ^(:)^ ≔ {Λ^(2)^, …, Λ^(*D*)^} corresponding to a series of class affinity tensors, each of which is *d*-way of size *C* × ⋯ × *C*. Note that to ensure *μ*^(*d*)^ is symmetric, Λ^(*d*)^ should also be symmetric, a condition our parameterization imposes.

Particular settings of Λ^(:)^ encode different mesoscale patterns regulating hyperedge formation. For example, a purely diagonal multi-tensor where ${\Lambda }_{{c}_{1}\ldots {c}_{d}}^{(d)}$ is only nonzero if *c*_1_ = … = *c*_*d*_ encodes strict assortativity. In fact, in this setting, the proposed model coincides exactly with Hypergraph-MT, a model recently proposed^6^. The proposed model class introduced in this paper is much more general, however, allowing for arbitrary higher-order interaction between classes.

The parametrization in Eq. (2) offers a parsimonious and interpretable framework for modeling hypergraphs but still presents significant practical challenges. While it drastically reduces the dimensionality of the original data ${{{\mathcal{A}}}}^{(:)}$, the number of parameters in Λ^(:)^ still grows exponentially in *D*, totaling ${\sum }_{d=2}^{D}{C}^{d}$ terms, a prohibitively large number for even moderate *C* and *D*. Moreover, this particular decomposition is generally non-unique^26^, complicating the interpretation of the estimated parameters.

We draw an analogy to the data and view Λ^(:)^ as itself a latent hypergraph of higher-order interactions among classes. We then posit that Λ^(:)^ admits a low-rank factorization:

3 $${\Lambda }_{{c}_{1}\ldots {c}_{d}}^{(d)}={\sum }_{k=1}^{K}{\gamma }_{k}^{(d)}{\prod }_{q=1}^{d}{w}_{{c}_{q}k},$$

where here we introduce a set of *K* class-level blocks we call communities. The *k*^th^ community is defined as a distribution over classes given by the column **w**_*k*_ of a *C* × *K* class-community membership matrix W, which is shared across *d* ∈ {2, …, *D*}. This matrix plays a role analogous to the node-class membership matrix Θ (see Fig. 1). However, unlike the factorization in Eqs. (2, 3), it imposes assortativity on the mesoscale structure of Λ^(:)^—i.e., that classes interact exclusively within (not between) communities. The rate of such interactions in a given community *k* is then given by ${\gamma }_{k}^{(d)} > 0$, which is allowed to vary by the order *d*. We refer to this term as the community-order rate. The ${\gamma }_{k}^{(d)}$ and W together parameterize Λ^(:)^, greatly reducing the number of model parameters. This factorization ensures that Λ^(:)^ is symmetric, which in turn ensures that *μ*^(:)^ is symmetric.

The parameterization in Eq. (3) allows nodes of different classes to interact disassortatively, as off-diagonal elements of Λ^(:)^ can be nonzero. Imposing that interactions among classes occur exclusively within communities mitigates the exponential blowup in parameters without encoding overly restrictive assumptions about the mesoscale structure of the observed hypergraph ${{{\mathcal{A}}}}^{(:)}$.

To realize a high degree of disassortativity—i.e., where Λ^(*d*)^ has much greater off- than on-diagonal elements—some of the parameters ${\gamma }_{k}^{(d)}$ must be allowed to be negative. If all are non-negative (and not trivially zero), we show in Supplementary Note 1 that the highest possible degree of disassortativity is bounded below by

4 $$\frac{{\sum }_{c=1}^{C}{\Lambda }_{c\ldots c}^{(d)}}{{\sum }_{{c}_{1}=1}^{C}\ldots {\sum }_{{c}_{d}=1}^{C}{\Lambda }_{{c}_{1}\ldots {c}_{d}}^{(d)}}\ge 1/{C}^{d-1}.$$

We refer to this sub-family of the model as semi-assortative, which allows for departure from strict assortativity, such as in core-periphery structure (and so it is useful in practice), but not to the extreme of strict disassortativity. We show in Supplementary Note 2 how to realize a fully omniassortative model by allowing some ${\gamma }_{k}^{(d)}$ to be negative and detail a careful modeling scheme that ensures all elements ${\Lambda }_{{c}_{1}\ldots {c}_{d}}^{(d)}$ remain non-negative.

Under a connection to tensor decomposition, the expression for ${{\mu }_{{{\bf{i}}}}^{(d)}}$ in Eq. (2) can be equivalently expressed for all entries **i** simultaneously as expressing the entire tensor *μ*^(*d*)^ as following a Tucker decomposition^27^:

5 $${\mu }^{(d)}=[{\Lambda }^{(d)};\Theta,\ldots,\Theta ]$$

where, in general, the first argument is the core tensor and the remaining arguments are the factor matrices, one for each mode of the input tensor. In this case, the factor matrices are *d* repeats of the same node-class membership matrix Θ, and the core is the class affinity tensor Λ^(*d*)^.

Similarly, the expression for ${\Lambda }_{{c}_{1}\ldots {c}_{d}}^{(d)}$ in Eq. (3) can be equivalently viewed as the tensor Λ^(*d*)^ following a special case of the Tucker decomposition, called the canonical polyadic (CP) decomposition^28^, where the core tensor is diagonal. See Fig. 1c for a visualization.

While the Tucker decomposition in Eq. (2) is not generally unique, we prove in Supplementary Note 1 that combining Eq. (3) with two additional assumptions, both of which are easily enforced, renders the model uniquely identified—an important property to ensure reliable parameter estimation. The first assumption is that the columns of Θ and W lie on the probability simplex, that is, their entries are non-negative and sum to 1. The second is that the first *C* columns of W form the identity matrix—i.e.,

6 $$[{{{\bf{w}}}}_{1},\ldots,{{{\bf{w}}}}_{C}]={{{\rm{I}}}}_{C}.$$

In enforcing the latter assumption, we impose that the number of communities exceeds the number of classes, so that *K *≥ *C*. This structure enforces that every class *c* has a corresponding pure community *k* = *c* within which no other classes have membership. In these communities, nodes interact in a strictly assortative manner.

These assumptions, satisfied in all of our experiments, allow for classes and communities to be identified from the data. This is a nontrivial result that arises from combining uniqueness results from tensor decomposition and non-negative matrix factorization^28,29^. For proofs and additional results, see Supplementary Note 1.

### Automatic discovery of drug classes and their interactions from large-scale pharmacological data

Here, we apply Omni-Hype-SMT to analyze the Drug Abuse Warning Network (DAWN) database^30^. In this setting, nodes are drugs, and each hyperedge represents a set of drugs that an emergency room patient self-reported to having taken. There are 2558 nodes and 141,178 hyperedges in this dataset; see Table 1 for additional details. Selecting *C* = 15 classes and *K* = 50 communities (refer to Supplementary Note 4 for details regarding the selection criteria), we fit the model to the entire dataset and perform an exploratory analysis of the inferred latent structure.

**Table 1:** Dataset summary statistics

Dataset	*K*	*C*	%(*d* = 2)	*D*	$\bar{d}$	${{{\mathcal{A}}}}^{(\bullet )}$	*N*_nz_	*N*	*C* grid	*K* grid
hospital (semi-synthetic)	3	2	72.5	5	2.3	8216	792	73	-	-
justice	6	3	0.0	9	6.4	1310	282	15	-	-
DAWN	48	16	22.4	16	4.0	834,643	141,178	2558	(16, 32, 48, 64)	(16, 32, 48, 64)
NDC-substances	64	32	17.9	25	8.0	29,296	6383	5539	(16, 32, 48, 64, 80)	(16, 32, 48, 64, 80)
workplace	8	5	94.1	4	2.1	9645	788	92	(5, 8, 11, 14)	(5, 8, 11, 14)
contact-high-school	15	12	70.3	5	2.3	172,035	7818	327	(9, 12, 15, 18)	(9, 12, 15, 18)
senate-bills	24	24	17.8	40	8.2	29,157	21,830	294	(12, 24, 36, 48)	(12, 24, 36, 48)
senate-committees	6	4	0.0	31	17.6	315	302	282	(2, 4, 6, 8, 10)	(2, 4, 6, 8, 10)

We report the number of communities *K*, classes *C*, the percentage of pairwise interactions %(*d* = 2), maximum hyperedge size *D*, mean hyperedge size $\bar{d}$, sum of hyperedges ${{{\mathcal{A}}}}^{(\bullet )}$, number of nonzero hyperedges *N*_nz_, number of nodes *N*, and the grids used to perform the grid search to select *C* and *K*.

**Fig. 3:**
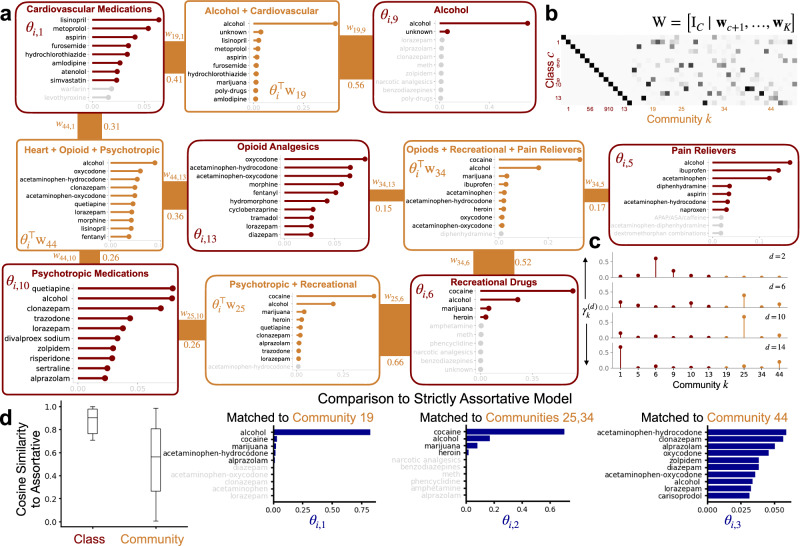
Omni-Hype-SMT learns a latent hypergraph between identified drug classes in drug-drug interaction data. **a** We highlight six classes of drugs $c\in \left\{1,5,6,9,10,13\right\}$ (maroon) and four communities, each defined by a convex combination of classes, $k\in \left\{19,25,34,44\right\}$ (bronze). The columns of the class-community matrix W (entries correspond to bronze links) define the class weights corresponding to each community. Here, communities may be interpreted as mixtures of classes. The width of the edge between the *c*^th^ class and *k*^th^ community is proportional to *w*_*ck*_, representing the weight of class *c* in community *k*. We show the top drugs occurring in each class, as measured by the node-class membership matrix Θ, and top drugs in each community, where the node-community loadings are given by the matrix multiplication ΘW (drugs with small nonzero values are grayed). **b** W, the class-community matrix. Each element *w*_*ck*_ is the weight of class *c* in community *k*, and each column sums to 1. **c** Conditional on the classes and communities shown in (**a**), for a fixed *d*, shown are the normalized community-order rates of each class and community given by ${\gamma }_{k}^{(d)}$. **d** A comparison to the strictly assortative model. Obtaining the closest assortative community (as measured by cosine similarity) to each community, we show the box plots (horizontal lines represent the minimum, first quartile, median, third quartile, and maximum, in ascending order) for the pure communities (*k *≤ 15) and mixture communities (*k *≥ 16). To the right, we show the closest strictly assortative community to each of the four communities shown in (**a**).

We begin by interpreting the inferred classes of drugs, represented by the node-class membership matrix Θ. In Fig. 3, we visualize six of the classes as maroon stem plots, where the drugs *i* with the largest values of *θ*_*ic*_ in each class *c* are shown. The labels given to each class are assigned by OpenAI’s GPT-4o (accessed May 4, 2025), which we prompted with a list of each class’s top drugs. We find that the inferred classes often represent groups of drugs with a shared function or purpose, such as cardiovascular medications (e.g., lisinopril, metoprolol), opioid analgesics (e.g., oxycodone, morphine), or psychotropic medications (e.g., quetiapine, clonazepam). We note that drugs in the same class are not necessarily drugs that are often found to have been taken together by ER patients. For example, while the drugs in the inferred cardiovascular medication class have a similar pharmacological function, they typically occur in our data with drugs of other inferred classes—i.e., this is a disassortative class. With the exception of alcohol, which is present in many classes due to its extreme prevalence in the data, we find that the classes’ top drugs are highly coherent and accord with the label assigned to them by GPT-4o.

We next inspect the inferred mixtures of classes represented by the class-community membership matrix W. Recall that each column of this matrix, **w**_*k*_, is the distribution over *C* classes defining community *k*. In Fig. 3 we visualize as bronze stem plots four different communities, where we show the top drugs *i* in each community *k* which have the largest values of ${{{\boldsymbol{\theta }}}}_{i}^{\top }{{{\bf{w}}}}_{k}={\sum }_{c=1}^{C}{\theta }_{ic}{w}_{ck}$. According to Eq. (3), the top drugs in community *k* should often occur in the data together in *d*-order hyperedges if there is a large value for the parameter ${\gamma }_{k}^{(d)}$. We chose to visualize these four communities because, according to W, they were all mainly a mixture of some subset of the six classes we chose to visualize—the bronze bars between classes and communities indicate that mixture, with the width of the bar corresponding to the magnitude of the mixture weight. The community labels are then just combinations of the relevant class labels.

One of these inferred communities represents a two-way mixture of psychotropic medications and recreational drugs and has large weights for *d* = 6 and *d* = 10, as shown in Fig. 3c. One hypothesis this supports is that some contingent of patients who self-reported taking many (e.g., *d* ≫ 2) drugs may have suffered a bad interaction between recreational drugs like cocaine and psychotropics like quetiapine. The particular mixture of quetiapine and cocaine—which are the top drugs in both classes, respectively—is actually referred to as a *Q-ball* and already known by researchers to cause increased sedation and cardiovascular strain, among other adverse effects^31^.

Given the structure we impose on W (see Eq. (6)), each class *c* is associated with a pure community *k* = *c*, which represents only assortative interactions between nodes of that class. We can see in Fig. 3c that for *d* = 2, a large weight is placed on *k* = 6, which is the pure community for the recreational drugs class (*c* = 6), suggesting that many ER patients who have only taken two drugs have taken two recreational drugs. We also see that for *d* = 14, a large weight is placed on community *k* = 1, which is the pure community for the cardiovascular class (*c* = 1), suggesting that many ER patients who have taken 14 drugs have taken 14 cardiovascular drugs. We surmise this is due to a non-causal correlation in the data—i.e., that patients who are on many cardiovascular medications are those who are already at high risk of heart attack, and wind up in the ER despite (not because of) the drugs.

Finally, we compare these qualitative findings with the strictly assortative baseline^6^ in Fig. 3d. We fit the strictly assortative model with *K* = *C* = 50 communities and match the communities found by Omni-Hype-SMT to its closest community (as measured by cosine similarity) recovered by the baseline. For the pure communities (*k* ∈ {1, …, 15}), or classes, the median cosine similarity is 0.91, while for the mixture communities (*k* ∈ {16, …, 50}, the value is 0.56. On the right, we show the strictly assortative community most closely resembling each bronze community shown in Fig. 3a.

Our findings indicate that Omni-Hype-SMT recovers a mixture structure that the baseline cannot. The closest assortative community to the Omni-Hype-SMT community 19 is dominated by alcohol and does not contain any cardiovascular medications. Similarly, the assortative model fails to capture the mixture with opioids, pain relievers and psychotropics. The closest communities to that of Omni-Hype-SMT (*k* = 25, 34) contain only recreational drugs, similar to Omni-Hype-SMT’s pure community (*c* = 6). It fails to mix with other classes (*c* = 5, 10, 13) to yield disassortative communities (*k* = 25, 34). The assortative model does best in the final case (*k* = 44), mixing psychotropics (*c* = 10) and opioids (*c* = 13), although it fails to capture the third component present in the community, the class of cardiovascular medications (*c* = 1). Even when the mixture structure is recovered, it is unclear whether the community represents a mixture of two distinct drug classes or one larger class. On the other hand, Omni-Hype-SMT unambiguously estimates a well-defined mixture of three distinct classes.

### Applicability across domains: prediction, interpretability, and heterogeneity

We consider six hypergraphs derived from empirical data: two drug interaction datasets (DAWN, NDC-substances)^5,30^, two congress-level datasets (senate-committees^32^, senate-bills^5,33,34^), and two additional human-contact interaction datasets (workplace^35^, contact-high-school^36^). Details on each dataset are provided in the “Methods” section and Table 1. These datasets span political, social, and pharmacological settings, but are not limited to them. Our method is just as applicable to other domains containing hypergraph data, such as ecological and co-authorship settings.

The proposed model allows for recovery of mesoscale structure by specifying the number of classes (*C*) and communities (*K*). To select the most plausible generative mechanism underlying the data, we compare the held-out likelihoods obtained by the model under various choices of *C* and *K* in a link prediction experiment and cross-validate to select the most appropriate *C* and *K*. Under strict assortativity, the best fit occurs when the number of classes and communities is equal (*K* = *C*). Otherwise, modeling omniassortative structure (setting *K* > *C*) offers gains in predictive performance.

Specifically, for each hyperedge order *d*, we randomly mask either 1000 of the nonzero counts or 10% of them, whichever is smaller. We also randomly mask an equal number of zeros. We denote the full set of masked hyperedges by ${\Omega }_{{{\rm{test}}}}^{(d)}$. We then fit the model to the unmasked portion of the data.

**Fig. 4:**
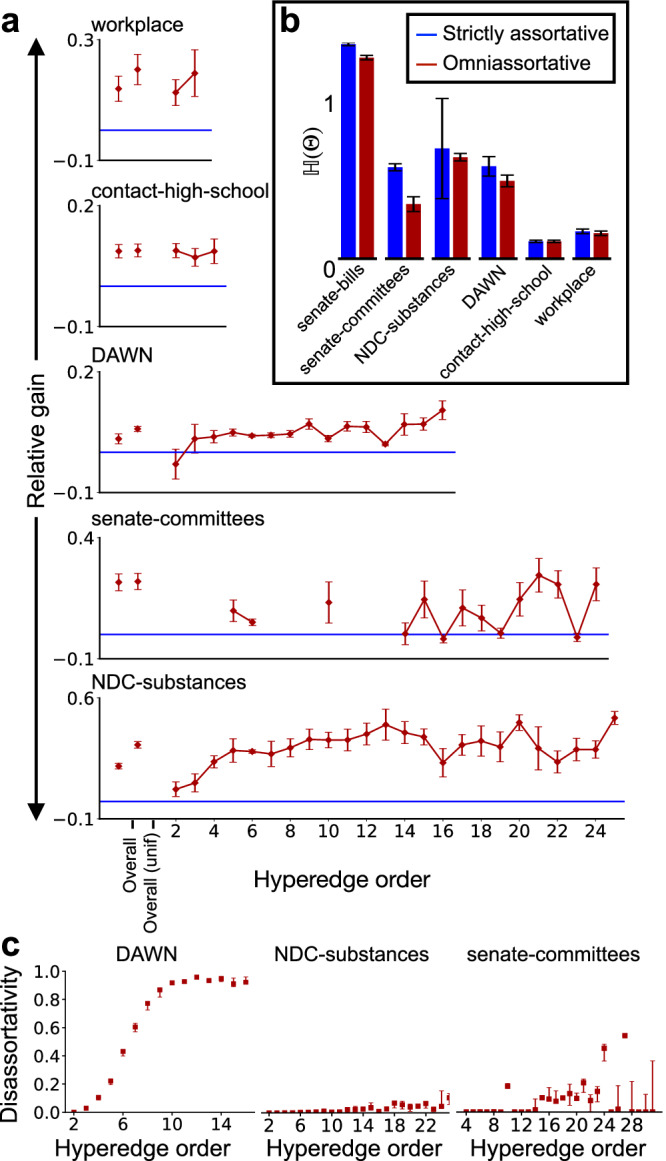
Relaxing strict assortativity improves interpretability and link prediction. **a** For each dataset, we show the relative gain in held-out log-likelihood over the strictly assortative baseline. Positive values indicate better link prediction for Omni-Hype-SMT, with error bars representing standard errors across five train-test splits. **b** Median entropy of ***θ***_*i*_ across nodes *i*, with error bars denoting its interquartile range across five random initializations. Lower values denote a more interpretable class structure with less uniform mixed-membership. **c** Inferred disassortativity levels vary by hyperedge order and dataset.

After training, we assess model fit using three metrics based on the log-likelihoods of the masked hyperedges. We employ multiple evaluation metrics because aggregated measures can obscure important differences in model fit. For instance, a model that performs well on pairwise interactions may achieve a similar overall log-likelihood to one that excels at higher-order interactions (*d *≥ 3), despite capturing fundamentally different structural aspects of the hypergraph. Therefore, we compute the heldout log-likelihood of each hyperedge **i** in the test set ${\Omega }_{{{\rm{test}}}}^{(d)}$ and compute

$${{{\mathcal{L}}}}^{(d)}={\sum }_{{{\bf{i}}}\in {\Omega }_{{\mathrm{test}}}^{(d)}}\log {{{\mathbb{P}}}}({A}_{{{\bf{i}}}}^{(d)}\,| \,{{\mu }_{{{\bf{i}}}}^{(d)}})$$

for each *d*. We also compute aggregate metrics

$${{\mathcal{L}}}={\sum }_{d=2}^{D}{{{\mathcal{L}}}}^{(d)}\,{{\rm{and}}}\,{{{\mathcal{L}}}}^{({{\rm{uniform}}})}={\sum }_{d=2}^{D}\frac{1}{| {\Omega }_{{{\rm{test}}}}^{(d)}| }{{{\mathcal{L}}}}^{(d)},$$

which are the heldout log-likelihood, and a weighted heldout log-likelihood which gives uniform weight to each order, respectively. These metrics are shown in Fig. 4a as overall and overall (unif).

For each dataset, we repeat this procedure over a grid of *C* and *K*, evaluating the combinations for which *C *≤ *K*. The choice of *C* and *K*, which achieves the highest ${{{\mathcal{L}}}}^{({{\rm{uniform}}})}$ value and the corresponding grids are reported in Table 1. In general, the user may apply the cross-validation procedure to select *C* and *K*. Additional model selection criteria, such as BIC or AIC, may be used if more appropriate for the application. When domain-specific knowledge is available, domain experts may specify a choice of *C* and *K* most appropriate for the application.

The log-likelihoods vary drastically, and we normalize them to compare results across datasets as follows. We compute relative gain over the strictly assortative baseline model^6^; for a given *d*, this is

$${{{\mathcal{RG}}}}^{(d)}=\frac{{{{\mathcal{L}}}}_{{{\rm{omni}}}}^{(d)}-{{{\mathcal{L}}}}_{{{\rm{assort}}}}^{(d)}}{| {{{\mathcal{L}}}}_{{{\rm{assort}}}}^{(d)}| },$$

with similar formulas applied to ${{\mathcal{L}}}$ and ${{{\mathcal{L}}}}^{({{\rm{uniform}}})}$.

In Fig. 4a, we show the relative gain metrics for the datasets where the omniassortative model performs best (five of the six datasets). That is, the highest values of ${{{\mathcal{L}}}}_{{\mathrm{omni}}}$ occur when *K* > *C*. Both overall metrics agree, yielding positive relative gains of varying amounts that range from 0.06 on the DAWN data to a peak of 0.33 on the NDC-substances data. The relative gain generally increases with hyperedge order.

Strict assortativity yields the highest prediction performance in senate bills (*C* = *K* = 24). This result is consistent with previous results obtained for this type of data^37^, where strong notions of group homophily are found in groups of congress members formed by cosponsorship of legislative bills. This dataset is not shown in Fig. 4a because all values are trivially zero.

Finally, we note that the standard errors of the predictive values over five train-test splits, as shown in Fig. 4a are relatively small across datasets, indicating that the communities inferred under edge removal do not change substantially across splits. We have run additional experiments on the Supreme Court and hospital datasets to further explore sensitivity to small perturbations and found little variation of the inferred communities learned from the full and perturbed datasets; see Supplementary Note 4 for details.

Beyond prediction, we assess whether a more expressive affinity tensor yields more interpretable inferred node-level memberships. For each dataset, we compare the proposed model with (*C*, *K*) as selected in the prediction task (for senate-bills, since the optimal combination occurs for *C* = *K* = 24, we choose *C* = 24 and *K* = 36) to the strictly assortative baseline^6^, where *C* = *K*. The models share the same choice of *C*. We fit each model to the full datasets and compute the empirical entropy of each node’s normalized class membership vector ${\bar{{{\boldsymbol{\theta }}}}}_{i}$. This value is given by ${\mathbb{H}}({\bar{{{\boldsymbol{\theta }}}}}_{i})=-{\sum }_{c=1}^{C}{\bar{\theta }}_{ic}\log ({\bar{\theta }}_{ic})$, which is non-negative. We compute the median value over nodes, reported as ${\mathbb{H}}(\Theta )$. In the extreme case, each node is assigned to exactly one class and ${\mathbb{H}}(\Theta )\,=\,0$. Lower values of ${\mathbb{H}}(\Theta )$ represent less mixed membership across classes and correspond to a more interpretable class structure.

Omni-Hype-SMT tends to infer less mixed class memberships than the strictly assortative model, as shown in Fig. 4b. The omniassortative model achieves lower ${\mathbb{H}}(\Theta )$ values for five of the six datasets (senate-bills, senate-committees, NDC-substances, DAWN, and workplace), while for contact-high-school, the values are similar. The difference is most apparent for Congress datasets, where the standard errors do not overlap.

We analyze how disassortativity varies with hyperedge order using Omni-Hype-SMT with *C* and *K* selected based on the link prediction results. Given the estimated model parameters, we assign hyperedges to class combinations (*c*_1_, …, *c*_*d*_). Interactions are strictly assortative when all classes are equal (*c*_1_ = ⋯ = *c*_*d*_) and non-assortative otherwise. For each hyperedge order, we compute the proportion of hyperedges involving multiple classes. We use this proportion as a measure of disassortativity; see the “Methods” section for further details regarding how it is defined and computed.

Figure 4c shows these proportions for three higher-order datasets. Each dataset contains higher-order interactions with maximum order (*D*) exceeding 15. In each setting, modeling omniassortativity improves prediction performance. The qualitative behavior of the curves varies by dataset. For example, in DAWN, we observe a monotonic increase in disassortativity until about order *d* = 10, after which the proportion plateaus near 1. These results illustrate the importance of carefully modeling omniassortativity in the hypergraph setting to appropriately capture underlying latent structure.

### Fast hypergraph generation

Finally, Omni-Hype-SMT is a generative model, and here we show how to use it to generate synthetic hypergraphs with prespecified mesoscale structures. The problem of hypergraph generation remains an open problem due to computational challenges^5,17,38^. However, exploiting properties of the proposed model allows us to address this task tractably. We describe an algorithm in the “Methods” section, which generates hypergraphs of arbitrarily-sized orders and whose computational complexity is linear in the expected number of hyperedges.

To illustrate this algorithm empirically, we generate synthetic hypergraph data using the model parameters learned on the DAWN data, as described in the pharmacological case study setting. Our method is fast: running on a personal laptop with one CPU, in 70 seconds it generates 833,564 higher-order drug interactions (similar in number to the true data), ranging from order *d* = 2 to *d* = 16.

**Fig. 5:**
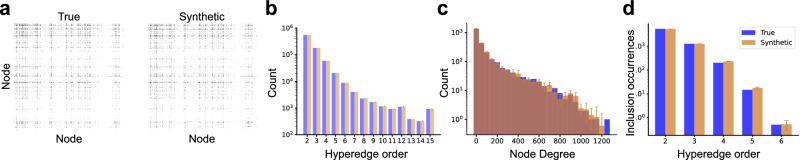
Generating synthetic data with Omni-Hype-SMT. We use Omni-Hype-SMT to generate data using parameters learned from fitting to the DAWN dataset. We use several metrics to compare the synthetic multi-tensor ${\widehat{{{\mathcal{A}}}}}^{(:)}$ to the true observed data ${{{\mathcal{A}}}}^{(:)}$. **a** Projected adjacency matrices of the true (left) and synthetic datasets (right). Hyperedge counts are shown in log scale. **b** Number of inclusion occurrences for each hyperedge order *d* in randomly sampled subsets; this is the number of nonzero hyperedges of size *d*, which appear as a subset of a hyperedge of size *d* + 1. **c** Empirical node degree distribution. **d** Empirical hyperedge order distribution. Error bars represent standard deviations over 5 random seeds.

To check whether the synthetic data is similar to the true data, we compare a number of statistics: the node-degree distributions, hyperedge order distributions, inclusion occurrence distributions^39^, and projected adjacency matrices. Figure 5 shows these comparisons. The plots appear nearly identical, demonstrating that the synthetic data closely resembles the true data.

## Discussion

This paper introduces a family of probabilistic generative models for higher-order interaction data that can represent and efficiently discover a broad spectrum of mesoscale structure in large-scale hypergraphs, from strictly assortative to disassortative. Different kinds of such structures offer fundamentally different descriptions of complex systems. It is thus critically important for understanding and predicting the behavior of such systems to be able to flexibly model a broad spectrum of structures in their underlying hypergraph.

We resolve the central computational challenge presented by higher-order interaction data by combining block modeling techniques with low-rank factorized representations. The proposed model blocks nodes into classes and then represents higher-order interactions among classes. The key insight is to view classes as themselves forming a latent hypergraph whose structure is strictly assortative. Unlike prior work, which assumes the observed hypergraph is assortative, this assumption still permits the observed graph to take on a broad range of structures, as we prove. The proposed model thus exploits the computational benefits of assortative structure among latent classes while still being able to represent and discover omniassortative structure among observed nodes.

Our theoretical results provide rigorous guarantees that guide and license the model’s use in a wide range of settings. Among them, we show that the model respects the symmetry of the data, that it generalizes existing strictly assortative models, and that it is identifiable.

Empirically, we demonstrated that the model is sufficiently flexible to capture a variety of latent mesoscale structures in a variety of higher-order network datasets arising from various real-world social, political, and biomedical settings. Initial comparisons between modeling approaches in two case studies, one within hospitals and another concerning Supreme Court Justices, underscore the proposed method’s significance. We illustrated how mesoscale structure can vary with hyperedge order, as the same class of nodes may behave assortatively in hyperedges of one order but disassortatively in another. We demonstrate the model’s scalability and expressivity through a case study of drugs taken by patients in emergency hospital visits. The proposed model is able to learn classes of drugs with similar function, and to predict which drugs of different classes are likely to have been taken together by ER patients. This is a nontrivial task that requires modeling both assortative and disassortative structure. We hope that our method motivates further work in this direction, as real-world, complex higher-order networks exhibit intricate omniassortative structure. As the network grows larger, the importance of appropriately accounting for this structure only increases.

Finally, we have shown how to efficiently sample synthetic hypergraphs with a pre-specified mesoscale structure under the proposed framework. We have presented an algorithm, derived from the model’s probabilistic properties, that is capable of rapidly producing large-scale hypergraphs exhibiting diverse structures and varying orders. We have empirically demonstrated the algorithm’s speed by quickly generating large hypergraphs at scale. Moreover, we have shown that the synthetic data closely matches real-world hypergraph data. This result has a far-reaching impact, potentially enabling researchers to study realistic higher-order networks of various structures in controlled settings.

Our method, while adaptable and flexible relative to existing approaches, has its limitations. One limitation is the extra computational cost required to model purely disassortative structure. If domain knowledge suggests that disassortativity is unlikely to arise in the application, this cost can be avoided. In general, though, it may not be known in advance what mesoscale structure could best explain a given dataset. Moreover, some hypergraph topologies may be too complex to be appropriately modeled by a low-rank factorization, such as hypergraphs with highly localized behavior. For these cases, more complex families of models may be more appropriate, but it is not clear how the handling of these cases would impact mathematical and computational tractability. Then, the proposed estimation algorithms provide point estimates for model parameters, which do not quantify uncertainty. For this, one would need to consider inference methods for estimating posterior distributions, which may require extra computational cost. The proposed model allows hyperedges of different orders to contribute differently to community structure. In several cases however, hyperedges are observed to express nestedness or other forms of hierarchy^40–43^. These may create strong correlations between hyperedges of different orders that may call for imposing explicit functional dependencies between these parameters, e.g., between parameters corresponding to hyperedges of subsequent orders. We considered here hypergraphs with discrete weights, but a natural model extension would be to allow for real-valued weights, such as through a compound Poisson construction^44,45^. Similarly, we focused here on static hypergraphs, where the set of nodes and hyperedges is fixed. In temporal hypergraphs, where the structure varies in time, it would be more appropriate to incorporate dynamical mechanisms for hyperedge formations directly into the model^46–50^. In the presence of node attributes in hypergraphs, beyond the information contained intrinsically in hyperedges, a natural extension would be to incorporate this extra information into the model formulation^51,52^. Finally, the proposed model targets undirected hypergraphs, where the order of nodes organized in hyperedges is not defined. A future direction would be to adapt our formulations to directed hypergraphs^53^, where one can identify a subset of nodes that play different roles, e.g., sender and receiver nodes in a network. In this setting, some symmetries break down, potentially allowing for the incorporation of ideas from asymmetric tensor decompositions for modeling multi-layer network data^23–25^.

## Methods

### Parameter estimation

To estimate the parameters Θ, W, and $\Gamma :={\left({\gamma }_{k}^{(d)}\right)}_{d,k}$, given the input data ${{{\mathcal{A}}}}^{(:)}$, we perform maximum likelihood estimation. Specifically, we maximize the log-likelihood, proportional in (Γ, W, Θ) to

7 $${{\mathcal{L}}}({{{\mathcal{A}}}}^{(:)},{\mu }^{(:)})=-{\sum }_{d=2}^{D}{\sum }_{{{\bf{i}}}\in {\Omega }^{(d)}}{{\mu }_{{{\bf{i}}}}^{(d)}}+{\sum }_{{A}_{{{\bf{i}}}}^{(d)} > 0}{A}_{{{\bf{i}}}}^{(d)}\log ({{\mu }_{{{\bf{i}}}}^{(d)}}),$$

where Ω^(*d*)^ is the set of all size-*d* combinations between *N* nodes such that $$| {\Omega }^{(d)}| \,=\,\left(\begin{array}{c}N\\ d\end{array}\right)$$.

We derive a generalized expectation-maximization (EM) algorithm^54^ specific to the proposed model which exploits the sparsity in the data and the low-rank, shared parameterization of the affinity tensors to quickly estimate parameters. A schematic visualization is given in Algorithm 1.

#### Algorithm 1

**EM Algorithm: general framework. The specific updates for the E-step and M-step depend on the model variant, as detailed in Supplementary Note** 2.

1: **Input:** Data: hyperedges, counts. Parameters: random restarts *R* = 10, convergence criteria, hyperparameters (*C*, *K*, learning rate), model (assort., semi, omni)

2: **Output:** model parameters (Γ^*^, Θ^*^, W^*^)

3: $${{{\mathcal{L}}}}^{(\max )}\leftarrow -\infty$$

4: **for** *r* = 1 **to** *R* **do**

5: randomly initialize Γ, Θ, W

6: **repeat**

7: **E-step,** **M-step:** update Γ, Θ, W

8: compute log-likelihood ${{\mathcal{L}}}$

9: **until** convergence

10: **if** $${{\mathcal{L}}} > {{{\mathcal{L}}}}^{(\max )}$$ **then**

11: $${{{\mathcal{L}}}}^{(\max )}\leftarrow {{\mathcal{L}}}$$

12: (Γ^*^, Θ^*^, W^*^) ← (Γ, Θ, W)

13: **end if**

14: **end for**

15: **return** (Γ^*^, Θ^*^, W^*^)

The algorithm relies on the Poisson latent subcount representation of the observed data^55,56^. Under this representation, each observed count ${A}_{{{\bf{i}}}}^{(d)}$ is the sum of *C*^*d*^ ⋅ *K* conditionally independent latent counts indexed by multi-index ***c*** = (*c*_1_, …, *c*_*d*_) and community index *k* ∈ [*K*], given by

8 $${A}_{{{\bf{i}}}{{\boldsymbol{c}}}k}^{(d)}{ \sim }^{{{\rm{ind.}}}}{{\rm{Poisson}}}\left({\gamma }_{k}^{(d)}{\prod }_{r=1}^{d}{w}_{{c}_{r}k}\,{\theta }_{{i}_{r}{c}_{r}}\right).$$

We model these latent counts to construct and maximize an evidence lower bound to the log-likelihood until the evidence lower bound converges or a stopping criterion is reached. The fixed point is guaranteed to be a local maximum, but not the global maximum. Therefore, we perform ten runs of the algorithm with different random initializations for (Γ, W, Θ), and take the fixed point with the highest log-likelihood as in Eq. (7). We discuss the generalized EM algorithm in greater detail in Supplementary Note 2, where we give exact updates and derivations for each of the strictly assortative, semi-assortative, and omniassortative models.

Alternatively, we may also place prior distributions on the parameters and extend to a maximum a posteriori inference procedure. While we do not do this in practice, we provide details in Supplementary Note 3.

The computational complexity of parameter estimation varies with parameterization. The tradeoff between model expressivity and complexity is reported in Table 2. The dominant term varies between *O*(*CN*_nz_) and *O*(*CKN*_nz_), where ${N}_{{{\rm{nz}}}}={\sum }_{d=2}^{D}| | {A}^{(d)}| {| }_{0}$ is the number of nonzero entries of ${{{\mathcal{A}}}}^{(:)}$, making the model scalable and applicable in practice. The fastest and least expressive model is the strictly assortative model. The most expressive—albeit most expensive—model is the omniassortative model, which includes an additional factor of *K* compared to the faster assortative version. The semi-assortative model offers a middle ground. We fit the semi-assortative model in all of our experiments except for those performed on the semi-synthetic hospital data. In Supplementary Note 4, we provide a runtime comparison between the the semi-assortative and omniassortative models, and show how the trade-off between runtime and performance varies by dataset.

**Table 2:** Tradeoff between expressivity and computational complexity

	Strictly assortative	Semi-assortative	Omni-assortative
E step	O(*CN*_nz_)	O(*KN*_nz_)	O(*KCN*_nz_)
M step	O(*NDC*)	O(*NDCK*)	O(*NDCK*)
Assortativity	*✓*	*✓*	*✓*
Core-periphery	✗	*✓*	*✓*
Disassortativity	✗	✗	*✓*

Here ${N}_{{{\rm{nz}}}}:={\sum }_{d=2}^{D}| | {A}^{(d)}| {| }_{0}$ is the number of nonzero entries of the input set of hypergraph adjacency tensors ${{{\mathcal{A}}}}^{(:)}$.

### Evaluation metrics

We consider several metrics to evaluate the proposed method. Here we provide more details describing and defining these metrics.

Several of the metrics in this paper are functions of latent subcounts^55,56^. Starting from the multi-index subcounts in Eq. (8), we can either consider latent community subcounts:

9 $${A}_{{{\bf{i}}}k}^{(d)}:={\sum }_{{c}_{1}=1}^{C}\ldots {\sum }_{{c}_{d}=1}^{C}{A}_{{{\bf{i}}}{{\boldsymbol{c}}}k}^{(d)}$$

or latent multi-class subcounts:

10 $${A}_{{{\bf{i}}}{{\boldsymbol{c}}}}^{(d)}:={\sum }_{k=1}^{K}{A}_{{{\bf{i}}}{{\boldsymbol{c}}}k}^{(d)}.$$

To interpret the mesoscale structure among classes, we compute a *C* × *C* class affinity matrix as:

11 $${\sum }_{d=2}^{D}{\sum }_{k=1}^{K}{\gamma }_{k}^{(d)}{{{\bf{w}}}}_{k}{{{\bf{w}}}}_{k}^{\top }.$$

This is an aggregate measure of affinity across communities and hyperedge orders. It is shown in Fig. 4a, b beneath the node-class memberships *Θ*.

To measure the mixed-membership levels learned by a model, we consider each node’s class membership vector given by ${{{\boldsymbol{\theta }}}}_{i}\in {{\mathbb{R}}}^{C}$. We first normalize each node’s class membership vector, dividing by ${\sum }_{c=1}^{C}{\theta }_{ic}$ to make it a valid discrete probability distribution over classes and compute its entropy.

For a vector **v**, the entropy is defined as

12 $${\mathbb{H}}({{\bf{v}}})=-{\sum }_{i}{v}_{i}\log ({v}_{i}),$$

where ${v}_{i}\log ({v}_{i})=0$ if *v*_*i*_ = 0. Higher entropy indicates greater mixing of a node’s class membership across different classes, which can hinder interpretability. In contrast, the extreme case of zero entropy, ${\mathbb{H}}({{\bf{v}}})=0$, corresponds to hard membership, where each node belongs to exactly one class.

We quantitatively measure the dissimilarity of a community indexed by *k* from an assortative class indexed by *c* using the Jensen-Shannon (JS) divergence between distributions, given by 

13 $${\mathrm{JS}}({{{\boldsymbol{\theta }}}}_{c\,}| | \,\Theta {{{\bf{w}}}}_{k})\,=\,\frac{1}{2}{\mathrm{KL}}({{{\boldsymbol{\theta }}}}_{c}\,| | \,{{{\boldsymbol{m}}}}^{(c,k)})$$

14 $$+\frac{1}{2}{\mathrm{KL}}(\Theta {{{\bf{w}}}}_{k}| | \,{{{\boldsymbol{m}}}}^{(c,k)}),$$

where ***m***^(*c*, *k*)^ is the mixture of ***θ***_*c*_ and Θ**w**_*k*_, given by ${{{\boldsymbol{m}}}}^{(c,k)}:=\frac{1}{2}({{{\boldsymbol{\theta }}}}_{c}+\Theta {{{\bf{w}}}}_{k})$. The Jensen-Shannon divergence is a symmetric alternative to the Kullback-Leibler (KL) divergence^57^. The lower its value, the more similar the two distributions are. For each community *k*, we compute the Jensen-Shannon divergence with each class *c* and select the minimum ${\min }_{c}{{\rm{JS}}}({{{\boldsymbol{\theta }}}}_{c}| | \Theta {{{\bf{w}}}}_{k})$ of these values. We consider this minimum value to be a measure of non-assortativity. For instance, if *w*_*ck*_ = 1 for a class *c* and 0 for all other classes, then Θ**w**_*k*_ = ***θ***_*c*_ and the proposed metric is equal to zero.

We also compute the expected number of hyperedges allocated to the *k*^th^ community, given by ${\sum }_{d=2}^{D}{\sum }_{{{\bf{i}}}\in {\Omega }^{(d)}}{A}_{{{\bf{i}}}k}^{(d)}$, where we use latent community subcounts as in Eq. (9). The summation over the space of possible hyperedges ${\bigcup }_{d=2}^{D}{\Omega }^{(d)}$ can be done efficiently, as we only need to sum over the nonzero entries of ${{{\mathcal{A}}}}^{(:)}$, which is linear in the number of observed hyperedges and usually linear in the number of nodes.

We use the two metrics described above to identify communities dissimilar from each class, which account for a meaningful portion of the observed hypergraph data (high allocation of hyperedges). In particular, we identify communities *k* = 19 and *k* = 44 as starting points for the exploratory analysis in the pharmacological case study.

For each hyperedge order *d*, we compute the expected proportion of hyperedges that are disassortative. Again, we rely on the latent subcount representation. Given latent subcounts ${A}_{{{\bf{i}}}{c}_{1}\ldots {c}_{d}}$, we compute

15 $$1-\frac{{\sum }_{{{\bf{i}}}\in {\Omega }^{(d)}}{\sum }_{c=1}^{C}{A}_{{{\bf{i}}}c\ldots c}^{(d)}}{{\sum }_{{{\bf{i}}}\in {\Omega }^{(d)}}{A}_{{{\bf{i}}}}^{(d)}},$$

where the numerator of the second term sums over assortative communities only (*c*, …, *c*). In practice, we cannot compute this term naively. Doing so requires summing over a combinatorial number of terms. Instead, we leverage the Poisson thinning property. In particular, for a hyperedge **i** and weight ${A}_{{{\bf{i}}}}^{(d)}$, we first distribute the weight across communities, as given by *A*_**i***k*_^*(d)*^. Then, for each community *k* and hyperedge **i**, we compute its disassortativity proportion, given by

16 $${\rho }_{{{\bf{i}}}k}^{({\mathrm{dis}})}=1-\frac{{\sum }_{c=1}^{C}\,{w}_{ck}^{d}{\prod }_{i\in {{\bf{i}}}}{\theta }_{ic}}{{\prod }_{i\in {{\bf{i}}}} \, {{{\bf{w}}}}_{k}^{\top }{{{\boldsymbol{\theta }}}}_{i}},$$

and multiply by *A*_**i***k*_^*(d)*^ We then add over all hyperedges and communities and divide by the sum of the hyperedges to reach the aggregate measure of disassortativity proportion given above:

17 $$\frac{{\sum }_{{{\bf{i}}},k}{A}_{{{\bf{i}}}k}^{(d)}\,{\rho }_{{{\bf{i}}}k}^{({\mathrm{dis}})}}{{\sum }_{{{\bf{i}}}\,}{A}_{{{\bf{i}}}}^{(d)}}.$$

In the semi-synthetic hospital experiment, we measure the area under the receiver-operator characteristic curve (AUC) on the held-out data to evaluate model performance. The binary categorization is whether each held-out hyperedge is greater than zero or zero. The AUC is equal to the probability the model assigns more probability to a randomly selected true positive hyperedge being positive than a randomly selected zero-weighted hyperedge^58^. The term

$${\mathbb{P}}({A}_{{\bf{i}}}^{(d)} > 0)\,=\,1-{e}^{-{{\mu }_{{{\bf{i}}}}}^{(d)}}$$

is increasing in ${{\mu }_{{{\bf{i}}}}^{(d)}}$, and so we compare rate parameters between hyperedges. That is, for the balanced heldout set ${\Omega }_{{{\rm{test}}}}^{(d)}$ consisting of an equal number of zeros and nonzeros, let the nonzeros be denoted by ${\Omega }_{{{\rm{test}}},1}^{(d)}$ and the zeros be denoted by ${\Omega }_{{{\rm{test}}},0}^{(d)}$. Then we compute the AUC by randomly pairing zero and nonzero hyperedges. For a given pair *p* in the set of pairs ${{\mathcal{P}}}$, we compute the AUC:

18 $${\mathrm{AUC}}\,=\,\frac{1}{| {{\mathcal{P}}}| }\left[{\sum }_{p}1({\mu }_{p,1} > {\mu }_{p,0})\,+\,0.5{\sum }_{p}1({\mu }_{p,0}\,=\,{\mu }_{p,1})\right].$$

*μ*_*p*,1_ and *μ*_*p*,0_ are the rates of the positive and zero-weighted hyperedges in pair *p*, respectively.

### Hypergraph generation

The aggregate hyperedge count

19 $${{{\mathcal{A}}}}^{(\bullet )}={\sum }_{d=2}^{D}{\sum }_{{{\bf{i}}}\in {\Omega }^{(d)}}{A}_{{{\bf{i}}}}^{(d)}$$

is marginally Poisson under Omni-Hype-SMT:

20 $${{{\mathcal{A}}}}^{(\bullet )} \sim {{\rm{Poisson}}}({\mu }^{(\bullet )}),\,{\mu }^{(\bullet )}:={\sum }_{d=2}^{D}{\sum }_{k=1}^{K}{\gamma }_{k}^{(d)}{\phi }_{k}^{(d)},$$

where ${\phi }_{k}^{(d)}:={\sum }_{{{\bf{i}}}\in {\Omega }^{(d)}}{\prod }_{i\in {{\bf{i}}}}{{{\bf{w}}}}_{k}^{\top }{{{\boldsymbol{\theta }}}}_{i}$. To sample a hypergraph, we first sample ${{{\mathcal{A}}}}^{(\bullet )}$ and thin ${{{\mathcal{A}}}}^{(\bullet )}$ over *d* and *k* into community counts *A*^(*d*, *k*)^. For each *d* and *k*, we sample *A*^(*d*, *k*)^ independent and identically distributed interactions by sampling *d* nodes (*i*_1_, …, *i*_*d*_) without replacement from the set of nodes [*N*], with sampling weight of node *i* proportional to ${m}_{ik}:={{{\bf{w}}}}_{k}^{\top }{{{\boldsymbol{\theta }}}}_{i}$. We denote the *i*^th^ interaction of order *d* and community *k* by ${{{\bf{e}}}}_{i}^{(d,k)}$. Each hyperedge is then defined as ${A}_{{{\bf{i}}}}^{(d)}:={\sum }_{k=1}^{K}{\sum }_{i=1}^{{A}^{(d,k)}}1{({{{\bf{e}}}}_{i}={{\bf{i}}})}^{(d,k)}$. Under this procedure, computational cost scales linearly in the count ${{{\mathcal{A}}}}^{(\bullet )}$ and rate *μ*^(•)^.

### Description of the datasets

We consider eight datasets in our experiments, as detailed in Table 1. Each dataset consists of a list of hyperedge occurrences (which we bin to create count-valued weights) and node labels.

We analyze three datasets (workplace, contact-high-school, hospital) collected by the SocioPatterns collaboration (http://www.sociopatterns.org). Each dataset describes the interactions between humans in close physical proximity, obtained by wearable sensors. The workplace dataset contains hyperedges that are contacts of employees across five different departments in an office building in France. The contact-high-school dataset describes interactions between students across nine classrooms. The hospital data describes interactions between and among patients and healthcare workers. The semi-synthetic hypergraph constructed from this data removes interactions that are exclusively among healthcare workers.

The following four datasets were constructed in https://www.cs.cornell.edu/~arb/data/. The first two concern interactions between substances. The Drug Abuse Warning Network (DAWN) data consists of interactions between drugs, where each interaction is the set of drugs in a patient (as reported by the patient) who visited the emergency department of one of multiple hospitals in the United States. The NDC-substances data consists of drugs found in the National Drug Code directory, where a hyperedge is a drug code consisting of the substances (nodes) that make up that drug.

The third and fourth datasets are United States Senate datasets, senate-bills and senate-committees. In each dataset, nodes are senators. In the bill co-sponsorship data, hyperedges are the set of senators who co-sponsor a bill in the Senate. We restrict our analysis to hyperedges of size *d *≤ *D* = 40. In the senate-committees data, hyperedges are the sets of senators serving as members of the same committee.

The justice hypergraph example is constructed from data found in http://scdb.wustl.edu/about.php. The dataset consists of United States Supreme Court cases and each United States Supreme Court Justice’s vote (for or against). Each node is a Justice, and each hyperedge corresponds to the set of Justices that sided with the majority opinion of a case. We consider cases with decisions from 2005–2024, coinciding with the time John Roberts served as Chief Justice up through the beginning of this study.

## Supplementary information

Supplementary Information

Transparent Peer Review file

## Data Availability

The datasets used in the paper are publicly available from their sources listed in the Methods section and in the Supplementary Materials.

## Code availability

An open-source algorithmic implementation of the model is publicly available and can be found at https://github.com/jhood3/omni-hype^62^.

## References

[1] Battiston, F. et al. Networks beyond pairwise interactions: structure and dynamics. *Phys. Rep.* **874**, 1–92 (2020).

[2] Porter, M. A., Onnela, J.-P. & Mucha, P. J. Communities in networks. *Notices of the AMS* **56** (2009).

[3] Fortunato, S. Community detection in graphs. *Phys. Rep.* **486**, 75–174 (2010).

[4] Antelmi, A. et al. A survey on hypergraph representation learning. *ACM Comput. Surv.* **56**, 1–38 (2023).

[5] Chodrow, P. S., Veldt, N. & Benson, A. R. Generative hypergraph clustering: from blockmodels to modularity. *Sci. Adv.* **7**, eabh1303 (2021). 34233880 10.1126/sciadv.abh1303 PMC11559555

[6] Contisciani, M., Battiston, F. & De Bacco, C. Inference of hyperedges and overlapping communities in hypergraphs. *Nat. Commun.* **13**, 7229 (2022). 36433942 10.1038/s41467-022-34714-7 PMC9700742

[7] Ruggeri, N., Contisciani, M., Battiston, F. & De Bacco, C. Community detection in large hypergraphs. *Sci. Adv.* **9**, eadg9159 (2023). 37436987 10.1126/sciadv.adg9159 PMC10337898

[8] Papachristou, M. & Kleinberg, J. Core-periphery models for hypergraphs. In *Proc. 28th ACM SIGKDD Conference on Knowledge Discovery and Data Mining*, 1337–1347 (ACM, 2022).

[9] Tudisco, F. & Higham, D. J. Core-periphery detection in hypergraphs. *SIAM J. Math. Data Sci.* **5**, 1–21 (2023).

[10] Sales-Pardo, M., Mariné-Tena, A. & Guimerà, R. Hyperedge prediction and the statistical mechanisms of higher-order and lower-order interactions in complex networks. *Proc. Natl. Acad. Sci. USA* **120**, e2303887120 (2023). 38060555 10.1073/pnas.2303887120 PMC10723119

[11] Ruggeri, N., Lonardi, A. & De Bacco, C. Message-passing on hypergraphs: detectability, phase transitions and higher-order information. *J. Stat. Mech.: Theory Exp.* **2024**, 043403 (2024).

[12] Brusa, L. & Matias, C. Model-based clustering in simple hypergraphs through a stochastic block model. *Scand. J. Stat.*10.1111/sjos.12754 (2024).

[13] Veldt, N., Benson, A. R. & Kleinberg, J. Hypergraph cuts with general splitting functions. *SIAM Rev.* **64**, 650–685 (2022).

[14] Pister, A. & Barthelemy, M. Stochastic block hypergraph model. *Phys. Rev. E* **110**, 034312 (2024). 39425428 10.1103/PhysRevE.110.034312

[15] Yoon, S.-E., Song, H., Shin, K. & Yi, Y. How much and when do we need higher-order information in hypergraphs? A case study on hyperedge prediction. In *Proc. Web Conference 2020*, 2627–2633 (ACM, 2020).

[16] Purkait, P., Chin, T.-J., Sadri, A. & Suter, D. Clustering with hypergraphs: the case for large hyperedges. *IEEE Trans. Pattern Anal. Mach. Intell.* **39**, 1697–1711 (2016). 28113490 10.1109/TPAMI.2016.2614980

[17] Ruggeri, N., Battiston, F. & De Bacco, C. Framework to generate hypergraphs with community structure. *Phys. Rev. E* **109**, 034309 (2024). 38632750 10.1103/PhysRevE.109.034309

[18] Vanhems, P. et al. Estimating potential infection transmission routes in hospital wards using wearable proximity sensors. *PLOS ONE* **8**, e73970 (2013). 24040129 10.1371/journal.pone.0073970 PMC3770639

[19] Holland, P. W., Laskey, K. B. & Leinhardt, S. Stochastic blockmodels: first steps. *Soc. Netw.* **5**, 109–137 (1983).

[20] Airoldi, E. M., Blei, D. M., Fienberg, S. E. & Xing, E. P. Mixed membership stochastic blockmodels. *J. Mach. Learn. Res.* **9**, 1981–2014 (2008). 21701698 PMC3119541

[21] Ball, B., Karrer, B. & Newman, M. E. Efficient and principled method for detecting communities in networks. *Phys. Rev. E* **84**, 036103 (2011). 10.1103/PhysRevE.84.036103 22060452

[22] Schein, A., Zhou, M., Blei, D. M. & Wallach, H. Bayesian Poisson Tucker decomposition for learning the structure of international relations. In *Proc. 33rd International Conference on Machine Learning*, 2810–2819 (JMLR.org., 2016).

[23] De Bacco, C., Power, E. A., Larremore, D. B. & Moore, C. Community detection, link prediction, and layer interdependence in multilayer networks. *Phys. Rev. E* **95**, 042317 (2017). 28505768 10.1103/PhysRevE.95.042317

[24] Aguiar, I., Taylor, D. & Ugander, J. A tensor factorization model of multilayer network interdependence. *J. Mach. Learn. Res.*, **25**, 1–54 (2024).

[25] Hood, J., Schein, A. J. The ALL0CORE tensor decomposition for sparse count data. In *International Conference on Artificial Intelligence and Statistics*, 4654–4662 (PMLR, 2024).

[26] Kolda, T. G. & Bader, B. W. Tensor decompositions and applications. *SIAM Rev.* **51**, 455–500 (2009).

[27] Tucker, L. R. Some mathematical notes on three-mode factor analysis. *Psychometrika* **31**, 279–311 (1966). 5221127 10.1007/BF02289464

[28] Hitchcock, F. L. The expression of a tensor or a polyadic as a sum of products. *J. Math. Phys.* **6**, 164–189 (1927).

[29] Gillis, N. *Nonnegative Matrix Factorization.* (SIAM, 2020).

[30] Benson, A. R., Abebe, R., Schaub, M. T., Jadbabaie, A. & Kleinberg, J. Simplicial closure and higher-order link prediction. *Proc. Natl. Acad. Sci.* **115**, E11221–E11230 (2018). 30413619 10.1073/pnas.1800683115 PMC6275482

[31] Waters, B. M. & Joshi, K. G. Intravenous quetiapine-cocaine use ("q-ball”). *Am. J. Psychiatry* **164**, 173–174 (2007). 17202567 10.1176/ajp.2007.164.1.173a

[32] Stewart III, C. & Woon, J. Congressional committee assignments, 103rd to 114th congresses, 1993–2017: senate. *Cambridge, MA: Massachusetts Institute of Technology* (2017).

[33] Fowler, J. H. Legislative cosponsorship networks in the U.S. House and Senate. *Soc. Netw.* **28**, 454–465 (2006).

[34] Fowler, J. H. Connecting the Congress: a study of cosponsorship networks. *Political Anal.* **14**, 456–487 (2006).

[35] Génois, M. et al. Data on face-to-face contacts in an office building suggest a low-cost vaccination strategy based on community linkers. *Netw. Sci.* **3**, 326–347 (2015).

[36] Mastrandrea, R., Fournet, J. & Barrat, A. Contact patterns in a high school: a comparison between data collected using wearable sensors, contact diaries and friendship surveys. *PLOS ONE* **10**, e0136497 (2015). 26325289 10.1371/journal.pone.0136497 PMC4556655

[37] Veldt, N., Benson, A. R. & Kleinberg, J. Combinatorial characterizations and impossibilities for higher-order homophily. *Sci. Adv.* **9**, eabq3200 (2023). 36608141 10.1126/sciadv.abq3200 PMC9821936

[38] Kamiński, B., Prałat, P. & Théberge, F. Hypergraph artificial benchmark for community detection (h–abcd). *J. Complex Netw.* **11**, cnad028 (2023).

[39] Lotito, Q. F., Musciotto, F., Montresor, A. & Battiston, F. Higher-order motif analysis in hypergraphs. *Commun. Phys.* **5**, 79 (2022).

[40] Mariani, M. S., Ren, Z.-M., Bascompte, J. & Tessone, C. J. Nestedness in complex networks: observation, emergence, and implications. *Phys. Rep.* **813**, 1–90 (2019).

[41] Landry, N. W., Young, J.-G. & Eikmeier, N. The simpliciality of higher-order networks. *EPJ Data Sci.* **13**, 17 (2024). 39677596 10.1140/epjds/s13688-024-00458-1 PMC11643508

[42] LaRock, T. & Lambiotte, R. Encapsulation structure and dynamics in hypergraphs. *J. Phys.: Complex.* **4**, 045007 (2023).

[43] Joslyn, C. A. et al. Hypernetwork science: from multidimensional networks to computational topology. In *International Conference on Complex Systems*, 377–392 (Springer, 2020).

[44] Zhou, M., Cong, Y. & Chen, B. Augmentable gamma belief networks. *J. Mach. Learn. Res.* **17**, 1–44 (2016).

[45] Basbug, M. & Engelhardt, B. Hierarchical compound Poisson factorization. In *International Conference on Machine Learning*, 1795–1803 (PMLR, 2016).

[46] Gallo, L., Lacasa, L., Latora, V. & Battiston, F. Higher-order correlations reveal complex memory in temporal hypergraphs. *Nat. Commun.* **15**, 4754 (2024). 38834592 10.1038/s41467-024-48578-6 PMC11150504

[47] Iacopini, I., Karsai, M. & Barrat, A. The temporal dynamics of group interactions in higher-order social networks. *Nat. Commun.* **15**, 7391 (2024). 39191743 10.1038/s41467-024-50918-5 PMC11349943

[48] Chowdhary, S., Kumar, A., Cencetti, G., Iacopini, I. & Battiston, F. Simplicial contagion in temporal higher-order networks. *J. Phys.: Complex.* **2**, 035019 (2021).

[49] He, X., Chodrow, P. S. & Mucha, P. J. Edge correlations and link prediction in growing hypergraphs. *Phys. Rev. E* **112**, 024305 (2025). 10.1103/4lkd-mtzq 40954746

[50] Benson, A. R., Kumar, R. & Tomkins, A. Sequences of sets. In *Proc. 24th ACM SIGKDD International Conference on Knowledge Discovery & Data Mining,* 1148–1157 (2018).

[51] Badalyan, A., Ruggeri, N. & De Bacco, C. Structure and inference in hypergraphs with node attributes. *Nat. Commun.* **15**, 7073 (2024). 39152121 10.1038/s41467-024-51388-5 PMC11329712

[52] M. Contisciani*, M. Hobbhahn*, E. A. Power, P. Hennig, C. De Bacco, Flexible inference in heterogeneous and attributed multilayer networks. *PNAS Nexus* pgaf005 (2025). 10.1093/pnasnexus/pgaf005 PMC11756377 39850077

[53] Gallo, G., Longo, G., Pallottino, S. & Nguyen, S. Directed hypergraphs and applications. *Discret. Appl. Math.* **42**, 177–201 (1993).

[54] Dempster, A. P., Laird, N. M., Rubin, D. B. Maximum likelihood from incomplete data via the em algorithm. *Journal of the Royal Statistical Society: Series B* **39**, 1–22 (1977).

[55] Schein, A. Allocative Poisson factorization for computational social science. PhD dissertation, *University of Massachusetts Libraries* (2019).

[56] Yıldırım, S., Kurutmaz, M. B., Barsbey, M., Şimşekli, U. & Cemgil, A. T. Bayesian allocation model: marginal likelihood-based model selection for count tensors. *IEEE J. Sel. Top. Signal Process.* **15**, 560–573 (2020).

[57] Kullback, S. & Leibler, R. A. On information and sufficiency. *Ann. Math. Stat.* **22**, 79–86 (1951).

[58] Fawcett, T. An introduction to ROC analysis. *Pattern Recognit. Lett.* **27**, 861–874 (2006).

[59] Eucalyp, Military. *Noun Project*. https://thenounproject.com/icon/military-3048399/ (2019).

[60] DinosoftLabs, a politician. *Noun Project*. https://thenounproject.com/icon/politician-2309034/ (2019).

[61] Freepik, Graduation hat and diploma. *Flaticon*. https://www.flaticon.com/free-icon/graduation-hat-and-diploma_42972 (2014).

[62] Hood, J., De Bacco, C. and Schein, A. Broad Spectrum Structure Discovery in Large-Scale Higher Order Networks: Omni-hype (v0.0). *Zenodo*. 10.5281/zenodo.18599065 (2026). 10.1038/s41467-026-71903-0 PMC13324705 42045213

[63] The Omni Group, OmniGraffle. *The Omni Group*. https://www.omnigroup.com/omnigraffle (2025).

