# Supplementary material for: Broad spectrum structure discovery in large-scale higher-order networks
Source: Nat Commun. 2026 Apr 27;17:5765. doi: 10.1038/s41467-026-71903-0 (PMC13324705; doi:10.1038/s41467-026-71903-0)
Supplement: Supplementary file 1 — Supplementary Information [file 41467_2026_71903_MOESM1_ESM.pdf]

Supplementary Information for:
"Broad Spectrum Structure Discovery in Large-Scale Higher-Order Networks"

John Hood,[1] Caterina De Bacco,[2] Aaron Schein[1]
[1] *University of Chicago*
[2] *Delft University of Technology*

CONTENTS

Supplementary Note 1: Theoretical properties 1
A. Summary of main theoretical results 1
Proofs 4

Supplementary Note 2: Inference 8
Background on the EM algorithm 8
Inference: the general pipeline 9
Model-specific updates 10

Supplementary Note 3: Additional mathematical details 13
Priors and MAP estimation 13
Graph (pairwise) setting 14
Properties of the Poisson 14
Computation 14

Supplementary Note 4: Experimental details 16

Supplementary Note 5: Detailed derivations 18

References 19

SUPPLEMENTARY NOTE 1: THEORETICAL PROPERTIES

Here we derive in more details the main theoretical properties described in the main manuscript. We first give a summary of the main results in Section A and then provide detailed proofs of each result in Section A. Most of these results focus on the semi-assortative model. Lemma A.8 lets us apply these results to the omniassortative model. Throughout, we assume the $C \leq K \leq N$ and the matrix $\Theta \in \mathbb{R}^{N \times C}$ is of (full) rank C, which holds almost surely. Our measure-theoretic results are with respect to the Lebesgue measure over the parameter space $\mathbb{R}_{>0}^{D-1 \times K} \times (\Delta^{C-1})^{K-C} \times (\Delta^{N-1})^{C}$ which contains the parameters $(\gamma_k^{(d)})_{d,k}$, $\mathbf{w}_{c+1}, \ldots, \mathbf{w}_K$ and Θ. For the omniassortative model, we consider negative values of $\gamma_c^{(d)}, c \in [C]$, as specified below.

A. Summary of main theoretical results

Recall that the expression for $\mu_{i_1 \ldots i_d}^{(d)}$ is given by

$$\mu_{i_1 \ldots i_d}^{(d)} = \sum_{c_1=1}^{C} \cdots \sum_{c_d=1}^{C} \Lambda_{c_1 \ldots c_d}^{(d)} \prod_{r=1}^{d} \theta_{i_r c_r}. \tag{S1}$$

The parameterization of $\Lambda^{(d)}$ is defined element-wise by

$$\Lambda_{c_1 \ldots c_d}^{(d)} = \sum_{k=1}^{K} \gamma_k^{(d)} \prod_{q=1}^{d} w_{c_q k}. \tag{S2}$$

Lemma A.1. *Each affinity tensor $\Lambda^{(d)}$ defined element-wise in Eq.* (S2) *is symmetric.*

Lemma A.1 ensures our reparameterization of the affinity tensor preserves the symmetric invariance to permutations of classes. The next two lemmas provide context for our statement on identifiability. The first draws upon the separable non-negative matrix factorization literature (1) to describe a unique matrix factorization.

Lemma A.2. *For* W*, as defined, the mapping* $(\Theta, \mathrm{W}) \to \Theta\mathrm{W}$ *is injective.*

The second gives conditions for which a CP tensor decomposition (2) is unique (3).

Lemma A.3 (Uniqueness of CP)**.** *The rank K CP decomposition of the tensor $A^{(d)} \in \mathbb{R}^{N\times\cdots\times N}$ given element-wise by*

$$A^{(d)}_{\mathbf{i}} = \sum_{k=1}^{K} \gamma^{(d)}_k \prod_{i\in\mathbf{i}} m_{ik}, \tag{S3}$$

is unique, up to permutation and scaling of the columns of the factor matrix $M = \Theta\mathrm{W} \in \mathbb{R}^{N\times K}$ for $\gamma^{(d)}_k, \sum_i m_{ik} > 0$ and $K \leq \frac{1}{2}(d(C-1)+1)$.

Remark. The decomposition is only unique, up to the permutation and scaling of the columns of M.
Justification. The reconstructed tensor remains invariant to arbitrary permutations π of the columns $[K]$, as $A^{(d)} = \sum_{k=1}^{K} \tilde{\gamma}_k \tilde{\boldsymbol{m}}^d_k = \sum_{k=1}^{K} \tilde{\gamma}_{\pi(k)} \tilde{\boldsymbol{m}}^d_{\pi(k)}$ for any permutation π, i.e., the sum does not change.
Invariance under scaling is proved as follows. For any decomposition parameterized by $(M, \boldsymbol{\gamma})$, given by

$$A^{(d)} = \sum_{k=1}^{K} \gamma_k \mathbf{m}^d_k = \sum_{k=1}^{K} \gamma_k \left(\mathbf{m}_k \circ \cdots \circ \mathbf{m}_k\right),$$

for arbitrary positive scaling constants $\psi_k > 0$, there exists another decomposition parameterized by $(\tilde{M}, \tilde{\boldsymbol{\gamma}})$, with:

$$\tilde{\gamma}_k := \gamma_k \left(\psi_k\right)^d, \quad \tilde{m}_{ik} := \frac{m_{ik}}{\psi_k}$$

such that

$$\begin{aligned}\sum_{k=1}^{K} \tilde{\gamma}_k \tilde{\boldsymbol{m}}^d_k = \sum_{k=1}^{K} \gamma_k (\psi_k)^d \left(\tfrac{\mathbf{m}_k}{\psi_k}\right)^d = \sum_{k=1}^{K} \gamma_k \frac{\psi^d_k}{\psi^d_k} \mathbf{m}^d_k \\ = \sum_{k=1}^{K} \gamma_k \mathbf{m}^d_k = A^{(d)}.\end{aligned}$$

This result implies the uniqueness of the assortative model as defined in Theorem A.4.

Theorem A.4 (*Uniqueness of the strictly assortative model*)**.** *Let $d \geq 3$, $\Lambda^{(d)}_{c_1\ldots c_d} = \begin{cases} \gamma^{(d)}_c & \text{if } c_j = c,\ \forall j \in [d] \\ 0 & \text{otherwise} \end{cases}$. Moreover, let $||\boldsymbol{\theta}_c||_1 = 1$ for each c. Then the decomposition given by*

$$\mu^{(d)}_{i_1\ldots i_d} = \sum_{c=1}^{C} \gamma^{(d)}_c \prod_{r=1}^{d} \theta_{i_r c}, \tag{S4}$$

is unique up to the permutation of classes.

Note that this result is valid for $d \geq 3$, a result of (3), which holds for tensors of $d \geq 3$. When $d = 2$, this model parameterizes a potentially non-unique, non-negative rank-C matrix factorization $\mu^{(d)} = \Theta S \Theta^\top$, where $S = \mathrm{diag}(\boldsymbol{\gamma})$. Despite this, Corollary A.7 shows that sharing parameters across $d \in \{2, \ldots, D\}$ generically identifies the model.

We combine Lemmas A.2 and A.3 to prove the following theorem, which states that under mild conditions (satisfied in all of our experiments) the parameters $(\Gamma, \Theta, \mathrm{W})$ uniquely determine $\mu^{(d)}$ for some $d \leq D$ under the semi-assortative parameterization.

Theorem A.5 (*Uniqueness*). *Let the following hold:* $d \geq 3, C < \mathit{Rank}(\Lambda^{(d)}) = K \leq \frac{1}{2}(d(C-1)+1), \mathrm{W} = \left[\mathrm{I}_C \mid \mathbf{w}_{c+1}, \ldots, \mathbf{w}_K\right] \in \mathbb{R}^{C \times K}, \Theta \in \mathbb{R}^{N \times C}, ||\Theta||_{1,1} = \sum_{i=1}^{N} \sum_{c=1}^{C} \theta_{ic} = C$*, where* W *is column stochastic. Then the symmetric Tucker decomposition of* $A \in \mathbb{R}^{N \times \cdots \times N}$ *with affinity tensor of rank* K *and dimension* $C \times \cdots \times C$*, given element-wise by*

$$A^{(d)}_{i_1 \ldots i_d} = \sum_{c_1=1}^{C} \cdots \sum_{c_d=1}^{C} \Lambda^{(d)}_{\boldsymbol{c}} \prod_{r=1}^{d} \theta_{i_r c_r}, \quad \Lambda^{(d)}_{\boldsymbol{c}} = \sum_{k=1}^{K} \gamma^{(d)}_k \prod_{c \in \boldsymbol{c}} w_{ck} \tag{S5}$$

and parameterized by $(\boldsymbol{\gamma}, \mathrm{W}, \Theta)$ *is unique, up to the permutation of classes* $[C]$ *and communities* $[K]$.

The condition that K is sufficiently small is not satisfied for all $d \in \{2, \ldots, D\}$, but it is satisfied for some $d \leq D$ in all of our experiments (the bound is increasing in d and C), which is sufficient to ensure our model is identifiable. In our experiments, the tightest bound occurs in the hospital setting, where the upper bound is tight: $\frac{1}{2}(D(C-1)+1) = \frac{1}{2}(5(2-1)+1) = 3 = K$.

Definition A.6 (*Generic identifiability*). We say that the model defined by Eqs. (S1) and (S2) is *generically identifiable* if

$$\mathbb{P}(\mathcal{A}^{(\cdot)} \mid \Gamma, \mathrm{W}, \Theta) = \mathbb{P}(\mathcal{A}^{(\cdot)} \mid \tilde{\Gamma}, \tilde{\mathrm{W}}, \tilde{\Theta})$$

if and only if

$$(\Gamma, \mathrm{W}, \Theta) = (\tilde{\Gamma}, \tilde{\mathrm{W}}, \tilde{\Theta}),$$

almost surely over the parameter space $\mathbb{R}^{D-1 \times K}_{>0} \times (\Delta^{C-1})^{K-C} \times (\Delta^{N-1})^{C}$ up to permutations of latent classes $[C]$ and communities $[K]$, where $\Delta^{p-1} := \{\mathbf{x} \in \mathbb{R}^p : \mathrm{x}_i \geq 0, ||\mathbf{x}||_1 = 1\}$ is the $p-1$-dimensional simplex in $\mathbb{R}^p$.

Corollary A.7 (Identifiability). *The model over the adjacency tensors* $\mathcal{A}^{(\cdot)} = \{A^{(2)}, \ldots, A^{(D)}\}$ *defined by Eqs.* (S1) *and* (S2) *is generically identifiable.*

Remark. Similarly, the model defined by the strictly assortative model is identifiable. Often, the separability condition $\mathrm{W} = [\mathrm{I}_C \mid \mathbf{w}_{c+1}, \ldots, \mathbf{w}_K]$ can be prohibitive, requiring optimization methods that identify C columns of W which correspond to the identity matrix I_C, where $w_{cc'} = 0$ unless $c = c'$, in which case $w_{cc'} = 1$, i.e., columns are one-hot encoded:

$$[\mathbf{w}_1, \ldots, \mathbf{w}_C] = \mathrm{I}_C.$$

Often, these columns correspond to observed variables or features in the data and cannot be assigned arbitrarily. However, in our setting, both dimensions of W are latent, and so we arbitrarily assign the first C columns as pivots at initialization and learn the community structure accordingly.

Lemma A.8 shows the omniassortative model may be alternatively parameterized as an extension of the semi-assortative model to include negative scaling constants.

Lemma A.8. *Let* $\mu^{(d)}$ *be the* $N \times \cdots \times N$ *tensor defined element-wise by the omniassortative model as defined according to Eq.* (S26)*. Then the reconstructed tensor* $\mu^{(d)}$ *has the CP decomposition representation*

$$\mu^{(d)}_{\mathbf{i}} = \sum_{k=1}^{K} \tilde{\gamma}^{(d)}_k \prod_{i \in \mathbf{i}} \boldsymbol{\theta}_i^\top \mathbf{w}_k,$$

where $\tilde{\gamma}^{(d)}_k = \begin{cases} \gamma^{(d)}_k - \sum_{k'=C+1}^{K} \gamma^{(d)}_{k'} w^d_{kk'} & \text{if } k \in [C] \\ \gamma^{(d)}_k & \text{otherwise.} \end{cases}$

Lemma A.8 lets us apply the results of Theorem A.5 to the omniassortative model.

Corollary A.9. *Under the same set of assumptions described in Theorem A.5, the decomposition defined by the omniassortative model is unique, up to permutation and scaling. Let* $||\mathbf{w}_k||_1 = 1$ *and* $||\Theta||_{1,1} = C$*. Then the decomposition is unique up to permutation of classes* $[C]$ *and communities* $[K]$.

By Theorem A.5, $\tilde{\Gamma}$ and Θ, and W are uniquely determined. For $c \in [C]$, we may solve for $\gamma^{(d)}_c = \tilde{\gamma}^{(d)}_c + \sum_{k=C+1}^{K} \tilde{\gamma}^{(d)}_k w^d_{ck}$ and for $k > C$, $\gamma^{(d)}_k = \tilde{\gamma}^{(d)}_k$. Leveraging this result, we claim the omniassortative model is generically identifiable.

Proofs

Proof of inequality S25.

Proof. The proof relies on Jensen's inequality, which states that for convex function $f : \mathbb{R} \to \mathbb{R}$

$$f(\tfrac{1}{n}\textstyle\sum_{i=1}^{n} x_i) \leq \displaystyle\frac{1}{n}\sum_{i=1}^{n} f(x_i).$$

Letting $f(x) = x^d$, which is convex for $x \geq 0$, $d \geq 1$ implies

$$\left(\frac{1}{C}\sum_{c=1}^{C} w_{ck}\right)^d = f\left(\tfrac{1}{C}\sum_{c=1}^{C} w_{ck}\right) \leq \frac{1}{C}\sum_{c=1}^{C} f(w_{ck}) = \frac{1}{C}\sum_{c=1}^{C} w_{ck}^d.$$

Consider an element $\Lambda^{(d)}_{c\dots c} = \sum_{k=1}^{K} \gamma_k^{(d)} \prod_{r=1}^{d} w_{c_r k} = \sum_{k=1}^{K} \gamma_k^{(d)} w_{ck}^d$ on the diagonal of $\Lambda^{(d)}$. Then

$$\begin{aligned}
\frac{1}{C}||\text{diag}(\Lambda^{(d)})||_{1,1} &= \frac{1}{C}\sum_{c=1}^{C} \Lambda^{(d)}_{c\dots c} \\
&= \frac{1}{C}\sum_{c=1}^{C}\sum_{k=1}^{K} \gamma_k^{(d)} \prod_{r=1}^{d} w_{c_r k} && (\text{def. of } \Lambda^{(d)}_{c\dots c}) \\
&= \sum_{k=1}^{K} \gamma_k^{(d)} \frac{1}{C}\sum_{c=1}^{C} w_{ck}^d && (c_r = c, \forall r \implies \prod_{r=1}^{d} w_{c_r k} = w_{ck}^d) \\
&\geq \sum_{k=1}^{K} \gamma_k^{(d)} \frac{1}{C^d}\left(\sum_{c=1}^{C} w_{ck}\right)^d && (\text{Jensen's}) \\
&= \frac{1}{C^d}\sum_{k=1}^{K} \gamma_k^{(d)} \sum_{c_1=1}^{C} \cdots \sum_{c_d=1}^{C} \prod_{r=1}^{d} w_{c_r k} \\
&= \frac{1}{C^d}\sum_{c_1=1}^{C} \cdots \sum_{c_d=1}^{C} \sum_{k=1}^{K} \gamma_k^{(d)} \prod_{r=1}^{d} w_{c_r k} \\
&= \frac{1}{C^d}\sum_{c_1=1}^{C} \cdots \sum_{c_d=1}^{C} \Lambda^{(d)}_{c_1\dots c_d} && (\Lambda^{(d)}_{c_1\dots c_d} = \sum_{k=1}^{K} \gamma_k^{(d)} \prod_{r=1}^{d} w_{c_r k}) \\
&= \frac{1}{C^d}||\Lambda^{(d)}||_{1,}.
\end{aligned}$$

Multiplying both sides by $\frac{C}{||\Lambda^{(d)}||_1}$ yields

$$\frac{||\text{diag}(\Lambda^{(d)})||_{1,1}}{||\Lambda^{(d)}||_{1,1}} \geq \frac{1}{C^{d-1}}. \tag{S6}$$

□

Proof of Lemma A.1.

Proof. Consider an element of the dth affinity tensor $\Lambda^{(d)}_{c_1\dots c_d} := \Lambda^{(d)}_{\boldsymbol{c}}$ and $\Lambda^{(d)}_{\pi(\boldsymbol{c})}$, where $\pi(\boldsymbol{c}) := (\pi(c_1), \dots, \pi(c_d))$ is an

arbitrary permutation of the indices $\boldsymbol{c} := (c_1, \ldots, c_d)$. Then $\Lambda^{(d)}_{\boldsymbol{c}} = \Lambda^{(d)}_{\pi(\boldsymbol{c})}$:

$$\begin{aligned}\Lambda^{(d)}_{\boldsymbol{c}} &= \sum_{k=1}^{K} \gamma_k^{(d)} \prod_{r=1}^{d} w_{c_r k} \\ &= \sum_{k=1}^{K} \gamma_k^{(d)} \left(w_{c_1 k}\, w_{c_2 k} \ldots w_{c_d k}\right) \\ &= \sum_{k=1}^{K} \gamma_k^{(d)} \left(w_{\pi(c_1)k}\, w_{\pi(c_2)k}\, \cdots\, w_{\pi(c_d)k}\right) \\ &= \sum_{k=1}^{K} \gamma_k^{(d)} \prod_{r=1}^{d} w_{\pi(\boldsymbol{c}_r)k} = \Lambda^{(d)}_{\pi(\boldsymbol{c})}.\end{aligned}$$

□

Proof of Lemma A.2.

Proof. Let $\Theta \mathrm{W} = M = \tilde{\Theta}\tilde{\mathrm{W}}$. Then

$$[\Theta \mid \Theta[\mathbf{w}_{c+1}, \ldots, \mathbf{w}_K]] = \Theta[\mathrm{I}_C \mid \mathbf{w}_{c+1}, \ldots, \mathbf{w}_K] = \Theta\mathrm{W} = M = \tilde{\Theta}\tilde{\mathrm{W}} = \tilde{\Theta}[\mathrm{I}_C \mid \tilde{\mathbf{w}}_{c+1}, \ldots, \tilde{\mathbf{w}}_K] = [\tilde{\Theta} \mid \tilde{\Theta}[\tilde{\mathbf{w}}_{c+1}, \ldots, \tilde{\mathbf{w}}_K]].$$

That is, $\Theta = \tilde{\Theta}$ and $\mathrm{W} = (\Theta^\dagger\Theta)\mathrm{W} = \Theta^\dagger(\Theta\mathrm{W}) = \Theta^\dagger M = \tilde{\Theta}^\dagger M = \tilde{\Theta}^\dagger\tilde{\Theta}\tilde{\mathrm{W}} = \tilde{\mathrm{W}}$ (assuming Θ is full rank), where $\Theta^\dagger$ denotes the Moore-Penrose pseudoinverse (4) of Θ. Together, $(\Theta, \mathrm{W}) = (\tilde{\Theta}, \tilde{\mathrm{W}})$. □

Proof of Lemma A.3.

Proof. We begin with the definition of *Kruskal rank.*

Definition A.10. (Kruskal rank) The **Kruskal rank** $\mathcal{K}_B$ of a matrix B is the maximal $\mathcal{K}_B$ such that any $\mathcal{K}_B$ columns of B are linearly independent.

The Kruskal rank of $M = \Theta\mathrm{W}$ is $\mathcal{K}_M = C$ for all $\Theta, \mathrm{W} \in (\Delta^{N-1})^C \times I_C \times (\Delta^{C-1})^{K-C}$ except for the measure-zero subset where the subsets of columns of W are linearly dependent or Θ is rank deficient.

The result is a corollary of (3), who extend Kruskal's theorem (5) to show the decomposition of the rank K tensor $A^{(d)} \in \mathbb{R}^{N_1 \times \cdots \times N_d}$ is unique (up to permutation and scaling) if $\sum_{i=1}^{D} \mathcal{K}_{B_i} \geq 2K + (D-1)$, where $\mathcal{K}_{B_i}$ is the Kruskal rank of the ith factor matrix $B_i \in \mathbb{R}^{N_i \times K}$. This result holds for tensors not restricted to be non-negative or symmetric. We apply the result to our setting, where $C = \mathcal{K}_i$ for each $i \in [D]$, which yields $DC \geq 2K + (D-1)$. Isolating K yields the sufficient condition $K \leq \frac{1}{2}(D(C-1)+1)$. □

Proof of Theorem A.4.

Proof. By Lemma A.3, Eq. (S4) is unique, up to permutation and scaling of the columns of Θ. That is, if (Γ, Θ) and $(\tilde{\Gamma}, \tilde{\Theta})$ parameterize Eq. (S4), then $\tilde{\gamma}_c^{(d)} = \frac{\gamma_{d\pi(c)}}{\psi_c^d}$ and $\tilde{\boldsymbol{\theta}}_c = \psi_c \boldsymbol{\theta}_{\pi(c)}$ for some permutation π and scalars $\psi_c > 0$. The ℓ_1 constraint on each column of Θ implies that $\psi_c = 1$ for all $c \in [C]$, that is,

$$1 = ||\tilde{\boldsymbol{\theta}}_c||_1 = ||\psi_c \boldsymbol{\theta}_{\pi(c)}||_1 = \psi_c ||\boldsymbol{\theta}_{\pi(c)}||_1 = \psi_c,$$

and so $\tilde{\boldsymbol{\theta}}_c = \boldsymbol{\theta}_{\pi(c)}$ for each c. □

Proof of Theorem A.5.

Proof. We plug in $\Lambda^{(d)}_{c_1\dots c_d} = \sum_{k=1}^K \gamma^{(d)}_k \prod_{r=1}^d w_{c_r k}$ to re-express Eq. (S5) as

$$A^{(d)}_{i_1\dots i_d} = \sum_{c_1=1}^C \cdots \sum_{c_d=1}^C \sum_{k=1}^K \gamma^{(d)}_k \prod_{r=1}^d w_{c_r k} \prod_{r=1}^d \theta_{i_r c_r} \qquad \text{(plugging in } \Lambda^{(d)}_{c_1\dots c_d} = \sum_{k=1}^K \gamma^{(d)}_k \prod_{r=1}^d w_{c_r k}) \tag{S7}$$

$$= \sum_{k=1}^K \gamma^{(d)}_k \sum_{c_1=1}^C \cdots \sum_{c_d=1}^C \prod_{r=1}^d w_{c_r k} \prod_{r=1}^d \theta_{i_r c_r} \qquad \text{(swap order of summation)} \tag{S8}$$

$$= \sum_{k=1}^K \gamma^{(d)}_k \prod_{r=1}^d \left(\sum_{c_r=1}^C w_{c_r k} \theta_{i_d c_r} \right) \qquad \text{(push sum into product)} \tag{S9}$$

$$= \sum_{k=1}^K \gamma^{(d)}_k \prod_{r=1}^d \boldsymbol{\theta}_{i_r}^\top \mathbf{w}_k \qquad \text{(simplify)} \tag{S10}$$

$$= \sum_{k=1}^K \gamma^{(d)}_k \prod_{r=1}^d m_{i_r k} \qquad m_{i_r k} := \boldsymbol{\theta}_{i_r}^\top \mathbf{w}_k. \tag{S11}$$

We make two observations, which help us finish the proof.

- Eq. (S11) parametrizes a (symmetric) rank K CP decomposition with factor matrix $M = \Theta \mathrm{W} \in \mathbb{R}^{N\times K}$. Lemma A.3 implies this decomposition is unique, up to permutations of the columns of M and scaling constants $\boldsymbol{\psi}$.
- By Lemma A.2, Θ and W uniquely construct $M = \Theta\mathrm{W}$.

Suppose that two distinct parameterizations $(\Gamma, \Theta, \mathrm{W})$ and $(\tilde{\Gamma}, \tilde{\Theta}, \tilde{\mathrm{W}})$ reconstruct A. Then Eq. (S11) implies that

$$\Theta\mathrm{W} = M, \tilde{\Theta}\tilde{\mathrm{W}} = \tilde{M},$$

where $M = \tilde{M}$, up to column permutation and scaling. We show that under the constraints $\mathrm{W} = \left[\mathrm{I}_C \mid \mathbf{w}_{c+1}, \dots, \mathbf{w}_K\right]$, $||\Theta||_{1,1} = C$, then $(\Theta, \mathrm{W}) = (\tilde{\Theta}, \tilde{\mathrm{W}})$ must hold.

By Eq. (S11), each column of $\tilde{M}$ and scalar $\tilde{\psi}_{dk}$ can be expressed as

$$\tilde{\boldsymbol{m}}_k = \psi_k \mathbf{m}_{\pi(k)}, \quad \tilde{\gamma}^{(d)}_k = \frac{\gamma_{d\pi(k)}}{\psi_k^d}.$$

Then

$$\sum_{c=1}^C \tilde{\theta}_{ic} \tilde{w}_{ck} = \tilde{\boldsymbol{m}}_{ik} = \psi_k \mathbf{m}_{i\pi(k)} = \psi_k \sum_{c=1}^C \theta_{ic} w_{c\pi(k)}.$$

Since $\sum_{c=1}^C w_{c\pi(k)} = \sum_{c=1}^C w_{ck} = 1$ it must hold that $\tilde{\boldsymbol{m}}_k = \psi_k \Theta \mathbf{w}_{\pi(k)}$, i.e., $\mathrm{W} = \tilde{\mathrm{W}}\Pi_K$, for some permutation matrix Π_K. Then $\tilde{\Theta} = \psi_k \Theta$ for all k, which implies that $\psi_k = \psi$ for all k. But then $C = ||\tilde{\Theta}||_{1,1} = ||\psi\Theta||_{1,1} = \psi||\Theta||_{1,1} = \psi C$ and so $\psi = 1$. But then $\Theta = \Pi_C \tilde{\Theta}$, for some permutation matrix Π_C, and so the two parameterizations are equivalent. □

Proof of Corollary A.7.

Proof. Suppose $\mathbb{P}(\mathcal{A}^{(\cdot)} \mid \Gamma, \mathrm{W}, \Theta) = \mathbb{P}(\mathcal{A}^{(\cdot)} \mid \tilde{\Gamma}, \tilde{\mathrm{W}}, \tilde{\Theta})$. Then for each $d \in \{2, \dots, D\}$, $i_1 < \cdots < i_d$, $\mu^{(d)}_{i_1\dots i_d} = \tilde{\mu}^{(d)}_{i_1\dots i_d}$. For $\mathbf{m}_k = \Theta\mathbf{w}_k \in \mathbb{R}^N_{>0}$,

$$\mu^{(d)}_{i_1\dots i_d} = \sum_{k=1}^K \gamma^{(d)}_k \mathbf{m}_k \circ \cdots \circ \mathbf{m}_k, \quad \tilde{\mu}^{(d)}_{i_1\dots i_d} = \sum_{k=1}^K \tilde{\gamma}^{(d)}_k \tilde{\boldsymbol{m}}_k \circ \cdots \circ \tilde{\boldsymbol{m}}_k$$

the "residual" tensor $R^{(d)} := \tilde{\mu}^{(d)} - \mu^{(d)}$ is zero on the indices $i_1 \neq \ldots \neq i_d$, and potentially nonzero elsewhere.

Thus, $R^{(d)}$ contains (at least) $\binom{N}{d}$ entries that are exactly zero, one for each $i_1 < \cdots < i_d$. Suppose that $R^{(d)}$ is not the 0 tensor, i.e., $R^{(d)} \neq 0$. By assumption, $\mathrm{Rank}(\mu^{(d)}) = K = \mathrm{Rank}(\tilde{\mu}^{(d)}) = \mathrm{Rank}(\mu^{(d)} + R^{(d)})$, which implies that

$R^{(d)}$ lies in the span of the rank-one tensors $(\mathbf{m}_k \circ \cdots \circ \mathbf{m}_k)_{k=1}^K$, i.e., $R^{(d)} = \sum_{k=1}^K r_k \mathbf{m}_k \circ \cdots \circ \mathbf{m}_k$ for some constants $r_k \in \mathbb{R}$. Since each entry $m_{ik} > 0$ and there are $K < \binom{N}{d}$ rank-1 tensors, $R^{(d)}$ lies outside the span of the K rank-one tensors almost surely. This implies that for $d \geq 3$, $\mu^{(d)} = \tilde{\mu}^{(d)}$.

Consider the case where $R^{(d)} = 0$, i.e., $\mu^{(d)} = \tilde{\mu}^{(d)}$. By Theorem A.5, for large enough d, $\mu^{(d)}$ is uniquely defined, with factor matrix $M = \Theta\mathrm{W}$, (which is uniquely defined by Θ and W), up to permutation. Therefore, it must be that $\Theta = \tilde{\Theta}$ and $\mathrm{W} = \tilde{\mathrm{W}}$. We remove scaling ambiguity of the constants $\gamma_k^{(d)}$ by requiring $||\mathbf{w}_k||_1 = ||\boldsymbol{\theta}_c||_1 = 1$, which implies that $||\mathbf{m}_k||_1 = \sum_{i=1}^N \sum_{c=1}^C \theta_{ic} w_{ck} = 1$.

Let $\boldsymbol{\gamma}_d = (\gamma_k^{(d)})_{k=1}^K$. Consider the matrix setting, $d = 2$ and suppose $\boldsymbol{\gamma}_2 \neq \tilde{\boldsymbol{\gamma}}_2$. The higher-order parameterization ($d \geq 3$) requires that Θ, W are unique and together they define $M = \Theta\mathrm{W}$. Suppose that $\mu_{ij}^{(2)} = \tilde{\mu}_{ij}^{(2)}$ for all $i \neq j$. Similar to above, $\mu_{ii}^{(d)} \neq \tilde{\mu}_{ii}^{(d)}$ occurs with probability 0. Thus, suppose $\mu^{(2)} = \tilde{\mu}^{(2)}$. Then $\Theta\mathrm{W}S\mathrm{W}^\top\Theta^\top = \Theta\mathrm{W}\tilde{S}\mathrm{W}^\top\Theta^\top$, and taking the pseudo-inverse of Θ implies that $\mathrm{W}S\mathrm{W}^\top = \mathrm{W}\tilde{S}\mathrm{W}^\top$. Since W is separable with l_1-norm constrained columns, it follows that $S = \tilde{S}$ (1). Similar for $d \geq 3$, the constants are uniquely defined almost surely.

For $(\Gamma, \Theta, \mathrm{W}) = (\tilde{\Gamma}, \tilde{\Theta}, \tilde{\mathrm{W}})$, the tensor reconstruction given by Eqs. (S1) and (S2) is well-defined, implying that

$$\mathbb{P}(\mathcal{A}^{(:)} \mid \Gamma, \mathrm{W}, \Theta) = \mathbb{P}(\mathcal{A}^{(:)} \mid \tilde{\Gamma}, \tilde{\mathrm{W}}, \tilde{\Theta}).$$

□

Proof of Lemma A.8.

Proof. Noting that for $C < k \leq K$, each sum over $c_1, \ldots, c_D$ may be written by computing the sum over all terms (ignoring the indicator), and then subtracting the terms s.t. $1(\exists c_i \neq c_j) = 0$, which occurs for $(c_1, \ldots, c_d) = (c, \ldots, c)$ for $c \in [C]$. For a given k, $C < k \leq K$,

$$\sum_{c_1=1}^C \cdots \sum_{c_d=1}^C 1(\exists c_i \neq c_j)\gamma_k^{(d)} \prod_{r=1}^d w_{c_r k}\theta_{i_r c_r} = \gamma_k^{(d)} \left(\sum_{c_1=1}^C \cdots \sum_{c_d=1}^C \prod_{r=1}^d w_{c_r k}\theta_{i_r c_r} - \sum_{c=1}^C w_{ck}^d \prod_{r=1}^d \theta_{i_r c_r} \right).$$

We re-express $\mu_{i_1 \ldots i_d}^{(d)}$ as

$$\begin{aligned}
\mu_{i_1 \ldots i_d}^{(d)} &= \sum_{c_1=1}^C \cdots \sum_{c_d=1}^C \sum_{k=1}^C \gamma_k^{(d)} \prod_{r=1}^d w_{c_r k}\theta_{i_r c_r} + \sum_{c_1=1}^C \cdots \sum_{c_d=1}^C 1(\exists c_i \neq c_j) \sum_{k=C+1}^K \gamma_k^{(d)} \prod_{r=1}^d w_{c_r k}\theta_{i_r c_r} && \text{(S12)} \\
&= \sum_{c=1}^C \gamma_c^{(d)} w_{cc}^d \prod_{r=1}^d \theta_{i_r c} + \sum_{k=C+1}^K \gamma_k^{(d)} \left(\sum_{c_1=1}^C \cdots \sum_{c_d=1}^C \prod_{r=1}^d w_{c_r k}\theta_{i_r c_r} - \sum_{c=1}^C w_{ck}^d \prod_{r=1}^d \theta_{i_r c} \right) && \text{(S13)} \\
&= \sum_{c=1}^C \gamma_c^{(d)} \prod_{r=1}^d \theta_{i_r c} + \sum_{k=C+1}^K \gamma_k^{(d)} \left(\prod_{r=1}^d \left(\sum_{c=1}^C w_{ck}\theta_{i_r c} \right) - \sum_{c=1}^C w_{ck}^d \prod_{r=1}^d \theta_{i_r c} \right) && \text{(S14)} \\
&= \sum_{c=1}^C \gamma_c^{(d)} \prod_{r=1}^d \theta_{i_r c} + \sum_{k=C+1}^K \gamma_k^{(d)} \left(\prod_{r=1}^d m_{i_r k} - \sum_{c=1}^C w_{ck}^d \prod_{r=1}^d \theta_{i_r c} \right). && \text{(S15)}
\end{aligned}$$

Combining like terms in Eq. (S15), we obtain

$$\begin{aligned}
\mu_{i_1 \ldots i_d}^{(d)} &= \sum_{c=1}^C \gamma_c^{(d)} \prod_{r=1}^d m_{i_r c} - \sum_{c=1}^C \sum_{k=C+1}^K \gamma_k^{(d)} w_{ck}^d \prod_{r=1}^d m_{i_d c} + \sum_{k=C+1}^K \gamma_k^{(d)} \prod_{r=1}^d m_{i_r k} \\
&= \sum_{c=1}^C \underbrace{\left(\gamma_c^{(d)} - \sum_{k=C+1}^K \gamma_k^{(d)} w_{ck}^d \right)}_{:=\tilde{\gamma}_c^{(d)}} \prod_{r=1}^d m_{i_r c} + \sum_{k=C+1}^K \underbrace{\gamma_k^{(d)}}_{:=\tilde{\gamma}_k^{(d)}} \prod_{r=1}^d m_{i_r k} \\
&= \sum_{k=1}^K \tilde{\gamma}_k^{(d)} \prod_{r=1}^d m_{i_r k}.
\end{aligned}$$

□

SUPPLEMENTARY NOTE 2: INFERENCE

Background on the EM algorithm

In maximum likelihood estimation, the goal is to find the parameter $\boldsymbol{\theta}$ that maximizes the likelihood function $p_{\boldsymbol{\theta}}(\mathbf{x})$ of the observed data $\mathbf{x}$ given $\boldsymbol{\theta}$. This entails solving

$$\widehat{\boldsymbol{\theta}} = \arg\max_{\boldsymbol{\theta}} p_{\boldsymbol{\theta}}(\mathbf{x}), \tag{S16}$$

or equivalently, maximizing the log-likelihood

$$\mathcal{L}(\mathbf{x}, \boldsymbol{\theta}) = \log p_{\boldsymbol{\theta}}(\mathbf{x}).$$

Often, the solution to Eq. (S16) is intractable. Starting at an initial point, the expectation-maximization (EM) algorithm (6) iteratively updates parameters according to a set of update rules specific to the problem at hand.

The EM algorithm is particularly useful when introducing a set of latent variables, denoted by $\mathbf{z}$, makes maximizing the complete log-likelihood $\log p_{\boldsymbol{\theta}}(\mathbf{x}, \mathbf{z})$ tractable. In this setting, the observed data likelihood is

$$p_{\boldsymbol{\theta}}(\mathbf{x}) = \int p_{\boldsymbol{\theta}}(\mathbf{x}, \mathbf{z})\, d\mathbf{z}.$$

We cannot maximize the complete log-likelihood (since $\mathbf{z}$ is unobserved), but we can maximize its expectation $\mathcal{Q}(\mathbf{x}, \boldsymbol{\theta}) = \mathbb{E}_{\mathbf{z}}[\log p_{\boldsymbol{\theta}}(\mathbf{x}, \mathbf{z}) \mid \mathbf{x}]$. $\mathcal{Q}(\mathbf{x}, \boldsymbol{\theta})$ is the *evidence lower bound*, a lower bound on the log-likelihood:

$$\begin{aligned}
\log p_{\boldsymbol{\theta}}(\mathbf{x}) &= \log \int p_{\boldsymbol{\theta}}(\mathbf{x}, \mathbf{z})\, d\mathbf{z} \\
&= \log \int p_{\boldsymbol{\theta}}(\mathbf{x}, \mathbf{z}) \frac{p_{\boldsymbol{\theta}}(\mathbf{z} \mid \mathbf{x})}{p_{\boldsymbol{\theta}}(\mathbf{z} \mid \mathbf{x})}\, d\mathbf{z} \\
&= \log \int p_{\boldsymbol{\theta}}(\mathbf{z} \mid \mathbf{x}) \frac{p_{\boldsymbol{\theta}}(\mathbf{x}, \mathbf{z})}{p_{\boldsymbol{\theta}}(\mathbf{z} \mid \mathbf{x})}\, d\mathbf{z} \\
&= \log \mathbb{E}_{\mathbf{z} \sim p_{\boldsymbol{\theta}}(\cdot \mid \mathbf{x})} \left[\frac{p_{\boldsymbol{\theta}}(\mathbf{x}, \mathbf{z})}{p_{\boldsymbol{\theta}}(\mathbf{z} \mid \mathbf{x})} \right] \\
&\geq \mathbb{E}_{\mathbf{z} \sim p_{\boldsymbol{\theta}}(\cdot \mid \mathbf{x})} \left[\log \frac{p_{\boldsymbol{\theta}}(\mathbf{x}, \mathbf{z})}{p_{\boldsymbol{\theta}}(\mathbf{z} \mid \mathbf{x})} \right] && \text{(by Jensen's inequality)} \\
&= \mathbb{E}_{\mathbf{z} \sim p_{\boldsymbol{\theta}}(\cdot \mid \mathbf{x})} \left[\log p_{\boldsymbol{\theta}}(\mathbf{x}, \mathbf{z}) \right] - \mathbb{E}_{\mathbf{z} \sim p_{\boldsymbol{\theta}}(\cdot \mid \mathbf{x})} \left[\log p_{\boldsymbol{\theta}}(\mathbf{z} \mid \mathbf{x}) \right] && \text{(decomposition into } \mathcal{Q} \text{ and entropy term)} \\
&= \mathcal{Q}(\mathbf{x}, \boldsymbol{\theta}) + \mathbb{H}(p(\mathbf{z} \mid \mathbf{x})) \\
&\geq \mathcal{Q}(\mathbf{x}, \boldsymbol{\theta}). && (\mathbb{H}(p(\mathbf{z} \mid \mathbf{x})) \geq 0)
\end{aligned}$$

Often, $p_{\boldsymbol{\theta}}(\mathbf{x}, \mathbf{z})$ is easier to maximize (with respect to $\boldsymbol{\theta}$) than $p_{\boldsymbol{\theta}}(\mathbf{x})$. Starting at initial point $\boldsymbol{\theta}_0$, the EM algorithm alternates between computing the expectation (E) step $\mathcal{Q}(\mathbf{x}, \boldsymbol{\theta}_t)$ and the maximization (M) step $\boldsymbol{\theta}_{t+1} = \arg\max_{\boldsymbol{\theta} \in \Theta} \mathcal{Q}(\mathbf{x}, \boldsymbol{\theta})$. The log-likelihood $\mathcal{L}(\mathbf{x}, \boldsymbol{\theta}_t)$ increases at each iteration t of the algorithm (6) and the sequence $(\mathcal{L}(\mathbf{x}, \boldsymbol{\theta}_t))_{t=0}^{\infty}$ converges to a local maxima (7).

When closed-form updates to $\boldsymbol{\theta}$ are not available, the *generalized* EM algorithm naturally extends the EM algorithm. Instead of finding the exact maximizer $\boldsymbol{\theta}_{t+1} = \arg\max_{\boldsymbol{\theta} \in \Theta} \mathcal{Q}(\mathbf{x}, \boldsymbol{\theta})$, the generalized EM algorithm requires only that the updated parameter increases the expected log-likelihood, i.e., $\mathcal{Q}(\mathbf{x}, \boldsymbol{\theta}_{t+1}) > \mathcal{Q}(\mathbf{x}, \boldsymbol{\theta}_t)$, at each iteration. The sequence $\{\mathcal{L}(\mathbf{x}, \boldsymbol{\theta}_t)\}_{t=0}^{\infty}$ is still guaranteed to converge to a local maximum.

Inference: the general pipeline

We derive a generalized EM algorithm (6) to maximize each model's log-likelihood, which is proportional in $(\Gamma, \mathrm{W}, \Theta)$ to

$$\mathcal{L}(\mathcal{A}^{(:)}, \upmu^{(:)}) = -\sum_{d=2}^{D}\sum_{\mathbf{i}\in\Omega^{(d)}} \mu_{\mathbf{i}}^{(d)} + \sum_{A_{\mathbf{i}}^{(d)}>0} A_{\mathbf{i}}^{(d)} \log(\mu_{\mathbf{i}}^{(d)}). \tag{S17}$$

We define the complete likelihood by introducing latent subcounts $A_{\mathbf{i}\mathbf{c}k}^{(d)}$ for each hyperedge $\mathbf{i} = (i_1, \ldots, i_d)$, combination of classes $\mathbf{c} = (c_1, \ldots, c_d)$ and community $k \in [K]$:

$$A_{\mathbf{i}\mathbf{c}k}^{(d)} \overset{\text{ind.}}{\sim} \text{Poisson}(\gamma_k^{(d)} \prod_{r=1}^{d} w_{c_r k} \prod_{r=1}^{d} \theta_{i_r c_r}), \quad A_{\mathbf{i}}^{(d)} = \sum_{c_1=1}^{C} \cdots \sum_{c_d=1}^{C}\sum_{k=1}^{K} A_{\mathbf{i}\mathbf{c}k}^{(d)}.$$

The evidence lower bound is proportional in $(\Gamma, \mathrm{W}, \Theta)$ to

$$\mathcal{Q}(\mathcal{A}^{(:)}, \upmu^{(:)}) = -\sum_{d=2}^{D}\sum_{\mathbf{i}\in\Omega^{(d)}} [\mu_{\mathbf{i}}^{(d)} + \sum_{c_1=1}^{C} \cdots \sum_{c_d=1}^{C}\sum_{k=1}^{K} \mathbb{E}\left[A_{\mathbf{i}\mathbf{c}k}^{(d)} \mid A_{\mathbf{i}}^{(d)}\right] \log(\mu_{\mathbf{i}\mathbf{c}k}^{(d)})], \tag{S18}$$

where $\mu_{\mathbf{i}\mathbf{c}k}^{(d)} := \gamma_k^{(d)} \prod_{r=1}^{d} \theta_{i_r c_r} w_{c_r k}$. At first glance, maximizing $\mathcal{Q}$ is computationally expensive. Evaluating $\mathcal{Q}$ requires computing the conditional expectation of the most fundamental latent subcounts $A_{\mathbf{i}\mathbf{c}k}^{(d)}$, given by

$$\mathbb{E}\left[A_{\mathbf{i}\mathbf{c}k}^{(d)} \mid A_{\mathbf{i}}^{(d)}\right] \propto \gamma_k^{(d)} \prod_{r=1}^{d} \theta_{i_r c_r} w_{c_r k},$$

and there are $C^d \cdot K$ of them for each multi-index $(i_1, \ldots, i_d)$. This introduces a cost of $\mathrm{O}(K \sum_{d=2}^{D} ||A^{(d)}||_0 C^d)$ over all hyperedges. However, all that are required are the expected sufficient statistics

$$\varphi_{\mathbf{i}k}^{(d)} = \sum_{c_1=1}^{C} \cdots \sum_{c_d=1}^{C} \mathbb{E}\left[A_{\mathbf{i}\mathbf{c}k}^{(d)} \mid A_{\mathbf{i}}^{(d)}\right],$$

$$\varphi_{ik} = \sum_{d=2}^{D} \sum_{\mathbf{i}\in\Omega^{(d)}, i\in\mathbf{i}} \varphi_{\mathbf{i}k}^{(d)}, \text{ and } \varphi_{ick} = \mathbb{E}[\sum_{d=2}^{D} \sum_{\mathbf{i}\in\Omega^{(d)}, i\in\mathbf{i}} \sum_{c_r : i_r \neq i} A_{\mathbf{i}\mathbf{c}k}^{(d)} \mid A_{\mathbf{i}k}^{(d)}].$$

These expectations are available in closed form and cost $\mathrm{O}(KN_{nz} + NCK)$ to compute, a cost linear in C and K. This cost is typically dominated by the first term, the number of nonzero hyperedges by the number of communities. The M-step iterates through parameters, maximizing $\mathcal{Q}$ with respect to each parameter, conditional on all other parameters fixed. We give closed-form expressions for the optimal $\gamma_k^{(d)}$ and θ_{ic} in each of the strictly, semi-, and omniassortative models, as well as derivations of these expressions in the next section. To update W, we use automatic differentiation (8), taking gradient steps to increase $\mathcal{Q}(\mathcal{A}^{(:)}, \upmu^{(:)})$. For a user-specified step size $\delta > 0$, we let $\nu_{ck} = \log(\exp(w_{ck}) - 1) \in \mathbb{R}$ and update $\boldsymbol{\nu}$, writing $\mathrm{W} = \mathrm{W}(\boldsymbol{\nu})$ as a function of $\boldsymbol{\nu}$ and use the update rule

$$\boldsymbol{\nu} \leftarrow \boldsymbol{\nu} + \delta \nabla_{\boldsymbol{\nu}} \mathcal{Q}(\mathrm{W}(\boldsymbol{\nu})), \quad w_{ck} \leftarrow \log(\exp(\nu_{ck}) + 1).$$

For sufficiently small δ, the update rule increases $\mathcal{Q}$. The cost of evaluating $\nabla_{\boldsymbol{v}} \mathcal{Q}$ is equal to the cost of evaluating the subset of $\mathcal{Q}$ dependent on $\boldsymbol{\nu}$, given by

$$\mathcal{B}(\boldsymbol{\nu}) = \Psi(\boldsymbol{\nu}) + \sum_{c=1}^{C}\sum_{k=1}^{K} \varphi_{ck} \log\left(w_{ck}(\nu_{ck})\right),$$

where $\Psi(\boldsymbol{\nu}) = -\sum_{d=2}^{D} \sum_{\mathbf{i}\in\Omega^{(d)}} \mu_{\mathbf{i}}^{(d)}(\boldsymbol{\nu})$ explicitly denotes the dependence of $\mu_{\mathbf{i}}^{(d)}$ on $\boldsymbol{\nu}$ and $\varphi_{ck} = \sum_{i=1}^{N} \varphi_{ick}$.We derive a dynamic programming algorithm to compute $\Psi(\boldsymbol{\nu})$ in $\mathrm{O}(NDK)$ time; each gradient evaluation costs $\mathrm{O}(NDK + KC)$. To preserve the pure community constraint, we keep the first C columns of W fixed and update the remaining parameters.

Model-specific updates

Here, we give specific updates in the E and M steps for each of the strictly assortative, semi-assortative, and omniassortative models.

Strictly assortative. The model is equivalent to Hypergraph-MT (9) parameterized by C communities. In particular, each observed hyperedge may be expressed as the sum of C latent subcounts:

$$A^{(d)}_{\mathbf{i}} = \sum_{c=1}^{C} A^{(d)}_{\mathbf{i}c},$$
$$A^{(d)}_{\mathbf{i}c} \overset{\text{ind.}}{\sim} \text{Poisson}(\gamma^{(d)}_c \prod_{i\in\mathbf{i}} \theta_{ic}).$$

The evidence lower bound (up to a scalar constant) is

$$\begin{aligned}
\mathcal{Q}(\mathcal{A}^{(:)}, \boldsymbol{\mu}^{(:)}) &= -\sum_{d=2}^{D}\sum_{\mathbf{i}\in\Omega^{(d)}} \mu^{(d)}_{\mathbf{i}} + \sum_{d=2}^{D}\sum_{\mathbf{i}\in\Omega^{(d)}}\sum_{c=1}^{C} \mathbb{E}[A^{(d)}_{\mathbf{i}c} \mid A^{(d)}_{\mathbf{i}}] \log \mu^{(d)}_{\mathbf{i}c} \\
&= -\sum_{d=2}^{D}\sum_{\mathbf{i}\in\Omega^{(d)}}\sum_{c=1}^{C} \mu^{(d)}_{\mathbf{i}c} + \sum_{d=2}^{D}\sum_{\mathbf{i}\in\Omega^{(d)}}\sum_{c=1}^{C} \mathbb{E}[A^{(d)}_{\mathbf{i}c} \mid A^{(d)}_{\mathbf{i}}] \log \mu^{(d)}_{\mathbf{i}c} \\
&= -\sum_{d=2}^{D}\sum_{c=1}^{C} \gamma^{(d)}_c \sum_{\mathbf{i}\in\Omega^{(d)}} \prod_{i\in\mathbf{i}} \theta_{ic} + \sum_{c=1}^{C}\sum_{d=2}^{D}\sum_{\mathbf{i}\in\Omega^{(d)}} \varphi^{(d)}_{\mathbf{i}c} \left(\log \gamma^{(d)}_c + \sum_{i\in\mathbf{i}} \log \theta_{ic} \right) \\
&= -\sum_{d=2}^{D}\sum_{c=1}^{C} \gamma^{(d)}_c \phi^{(d)}_c + \sum_{c=1}^{C}\sum_{d=2}^{D}\sum_{\mathbf{i}\in\Omega^{(d)}} \varphi^{(d)}_{\mathbf{i}c} \left(\log \gamma^{(d)}_c + \sum_{i\in\mathbf{i}} \log \theta_{ic} \right) \\
&= -\sum_{d=2}^{D}\sum_{c=1}^{C} \gamma^{(d)}_c \phi^{(d)}_c + \sum_{c=1}^{C}\sum_{d=2}^{D}\sum_{A^{(d)}_{\mathbf{i}}>0} \varphi^{(d)}_{\mathbf{i}c} \left(\log \gamma^{(d)}_c + \sum_{i\in\mathbf{i}} \log \theta_{ic} \right).
\end{aligned}$$

E-step. Conditional on $A^{(d)}_{\mathbf{i}} > 0$, the vector of latent subcounts $(A^{(d)}_{\mathbf{i}c})_{c=1}^{C}$ is multinomial distributed, where

$$(A^{(d)}_{\mathbf{i}c})_{c=1}^{C} \mid A^{(d)}_{\mathbf{i}} \sim \text{Multinomial}\left(A^{(d)}_{\mathbf{i}}, \frac{\gamma^{(d)}_c \prod_{i\in\mathbf{i}} \theta_{ic}}{\sum_{c'=1}^{C} \gamma^{(d)}_{c'} \prod_{i\in\mathbf{i}} \theta_{ic'}} \right).$$

The expected latent subcounts are given by

$$\varphi^{(d)}_{\mathbf{i}c} = \mathbb{E}[A^{(d)}_{\mathbf{i}c} \mid A^{(d)}_{\mathbf{i}}] = A^{(d)}_{\mathbf{i}} \cdot \frac{\gamma^{(d)}_c \prod_{i\in\mathbf{i}} \theta_{ic}}{\sum_{c'=1}^{C} \gamma^{(d)}_{c'} \prod_{i\in\mathbf{i}} \theta_{ic'}}. \tag{S19}$$

We add them together to obtain $\varphi_{ic} = \sum_{d=2}^{D} \sum_{\mathbf{i}\in\Omega^{(d)}} \varphi^{(d)}_{\mathbf{i}c}$ and $\varphi^{(d)}_c = \sum_{\mathbf{i}\in\Omega^{(d)}} \varphi^{(d)}_{\mathbf{i}c}$.

M-step. The optimal coordinate-wise updates are found by setting the partial derivative (with respect to a given parameter) to 0 and solving for the given parameter. The optimal updates are given by

$$\theta_{ic} = \frac{\varphi_{ic}}{\sum_{d=2}^{D} \gamma^{(d)}_c \bar{\phi}^{(d-1)}_{ic}}, \quad \gamma^{(d)}_c = \frac{\varphi^{(d)}_c}{\phi^{(d)}_c}, \tag{S20}$$

where $\phi^{(d)}_c$ is defined as $\phi^{(d)}_c := \sum_{\mathbf{i}\in\Omega^{(d)}} \prod_{i\in\mathbf{i}} \theta_{ic}$ and $\bar{\phi}^{(d)}_{ic} := \sum_{\mathbf{i}\in\Omega^{(d)}, i\notin\mathbf{i}} \prod_{j\in\mathbf{i}} \theta_{jc}$.

Semi-asssortative. The model is given by

$$A^{(d)}_{\mathbf{i}} = \sum_{c_1=1}^{C} \cdots \sum_{c_d=1}^{C} \sum_{k=1}^{K} A^{(d)}_{\mathbf{i}\mathbf{c}k}, \tag{S21}$$

$$A^{(d)}_{\mathbf{i}\mathbf{c}k} \overset{\text{ind.}}{\sim} \text{Poisson}(\gamma^{(d)}_k \prod_{q=1}^{d} (w_{c_q k} \theta_{i_q c_q})). \tag{S22}$$

The EM algorithm is given in Algorithm 1 and alternates between the E-step, computing the expectations conditional on $(\Gamma, \mathrm{W}, \Theta)$ using Eq. (S23) and Eq. (S24), and the M-step, maximizing $\mathcal{Q}(\mathcal{A}^{(\cdot)}, \upmu^{(\cdot)})$ with respect to the parameters $(\Gamma, \mathrm{W}, \Theta)$.

For $\mathbf{i} = (i_1, \ldots, i_d)$, the conditional expectation $\mathbb{E}[A^{(d)}_{\mathbf{i}k} \mid A^{(d)}_{\mathbf{i}}] = \varphi^{(d)}_{\mathbf{i}k}$ is given by

$$\varphi^{(d)}_{\mathbf{i}k} = A^{(d)}_{\mathbf{i}} \cdot \frac{\gamma^{(d)}_k \prod_{i \in \mathbf{i}} \boldsymbol{\theta}_i^\top \mathbf{w}_k}{\sum_{k'=1}^K \gamma^{(d)}_{k'} \prod_{i \in \mathbf{i}} \boldsymbol{\theta}_i^\top \mathbf{w}_{k'}}, \tag{S23}$$

under the multinomial thinning property of the Poisson. Then, we have:

$$\mathbb{E}[A^{(d)}_{ick} \mid \mathcal{A}^{(\cdot)}] = \varphi^{(d)}_{ick} = \frac{\theta_{ic} w_{ck}}{\sum_{c'=1}^C \theta_{ic'} w_{c'k}} \cdot \sum_{d=2}^D \sum_{\mathbf{i} \in \Omega^{(d)}} \varphi^{(d)}_{\mathbf{i}k} 1(i \in \mathbf{i}). \tag{S24}$$

For full derivations of the optimal updates we refer to Supplementary Note 5.

Algorithm 1 EM algorithm for the semi-assortative model.

Require: Adjacency tensors $\mathcal{A}^{(\cdot)}$, initialized parameters $\Gamma, \mathrm{W}, \Theta$, step size $\delta > 0$, stopping criterion.

repeat

E-step: compute the expected latent subcounts as in Eqs. (S23) and (S24)

$$\varphi^{(d)}_{\mathbf{i}k} \leftarrow A^{(d)}_{\mathbf{i}} \cdot \frac{\gamma^{(d)}_k \prod_{i \in \mathbf{i}} \boldsymbol{\theta}_i^\top \mathbf{w}_k}{\sum_{k'=1}^K \gamma^{(d)}_{k'} \prod_{i \in \mathbf{i}} \boldsymbol{\theta}_i^\top \mathbf{w}_{k'}}$$

$$\varphi_{ik} \leftarrow \sum_{d=2}^D \sum_{\mathbf{i} \in \Omega^{(d)}, i \in \mathbf{i}} \varphi^{(d)}_{\mathbf{i}k}, \qquad \varphi_{ick} \leftarrow \varphi_{ik} \cdot \frac{w_{ck} \theta_{ic}}{\sum_{c'=1}^C w_{c'k} \theta_{ic'}}$$

M-step: update the parameters as in Eq. (S20)

$$\gamma^{(d)}_k \leftarrow \frac{\sum_{\mathbf{i} \in \Omega^{(d)}} \varphi^{(d)}_{\mathbf{i}k}}{\phi^{(d)}_k}$$

$$\textbf{for } i \in [N] : \theta_{ic} \leftarrow \frac{\sum_{k=1}^K \varphi_{ick}}{\sum_{k=1}^K w_{ck} \left(\sum_{d=2}^D \gamma^{(d)}_k \bar{\phi}^{(d-1)}_{ik} \right)}$$

$$\text{update } \boldsymbol{\phi}, (\bar{\phi}^{(d)}_{i+1,k})_{d=2}^D$$

$$\boldsymbol{\nu} \leftarrow \boldsymbol{\nu} + \delta \nabla \mathcal{Q}(\mathrm{W}(\boldsymbol{\nu})), \quad \mathrm{W} \leftarrow \log(\exp(\boldsymbol{\nu}) + 1)$$

compute $\boldsymbol{\phi}, (\bar{\boldsymbol{\phi}}^{(d)})_{d=2}^D$

until stopping criterion met

return $\Gamma, \mathrm{W}, \Theta$

Omniassortative. In Supplementary Note 1, we show that the semi-assortative model is constrained to satisfy the inequality

$$\frac{\sum_{c=1}^C \sum_{k=1}^K \Lambda^{(d)}_{c\ldots ck}}{\sum_{c_1=1}^C \cdots \sum_{c_d=1}^C \sum_{k=1}^K \Lambda^{(d)}_{c_1 \ldots c_d k}} \geq \frac{1}{C^{d-1}}, \tag{S25}$$

where $\Lambda^{(d)}_{c_1 \ldots c_d k} = \gamma^{(d)}_k \prod_{q=1}^d w_{c_q k}$, such that $\Lambda^{(d)}_{c_1 \ldots c_d} = \sum_{k=1}^K \Lambda^{(d)}_{c_1 \ldots c_d k}$. To relax the bound, we impose that for $k > C$,

the diagonal elements $\Lambda^{(d)}_{c\ldots ck} = 0$. Under this modification, we express each affinity tensor as

$$\Lambda^{(d)}_{c_1\ldots c_d k} = 1(k \leq C \text{ or } \exists c_i \neq c_j)\gamma^{(d)}_k \prod_{q=1}^{d} w_{c_q k}. \tag{S26}$$

Under this modification, inequality (S25) is not necessarily satisfied and we may capture pure disassortativity (i.e., $\Lambda^{(2)}_{c_1 c_1} \approx 0$ but $\Lambda^{(2)}_{c_1 c_2} \gg 0$). Under this adaptation, we derive a similar generalized EM algorithm with closed-form updates to Θ and Γ as in the semi-assortative setting. The adaptation requires an additional factor of C in the computational cost of the E-step; see Algorithm 2 for more details.

The evidence lower bound is

$$\mathcal{Q}(\mathcal{A}^{(:)}, \boldsymbol{\mu}^{(:)}) = \sum_{d=2}^{D} \sum_{\mathbf{i}\in\Omega^{(d)}} \sum_{k=1}^{K} \sum_{c_1=1}^{C} \cdots \sum_{c_d=1}^{C} \left(1(k \leq C \text{ or } \exists c_i \neq c_j)\gamma^{(d)}_k \prod_{r=1}^{d} (\theta_{i_r c_r} w_{c_r k}) + A^{(d)}_{\mathbf{i}ck} \left(\log \gamma^{(d)}_k + \sum_{r=1}^{d} \log(\theta_{i_r c_r} w_{c_r k}) \right) \right).$$

The optimal update for θ_{ic} is

$$\theta_{ic} = \frac{\sum_{k=1}^{K} \varphi_{ick}}{\sum_{d=2}^{D} \gamma^{(d)}_c \bar{\phi}^{(d-1)}_{ic} + \sum_{k=C+1}^{K} \gamma^{(d)}_k w_{ck} \left(\bar{\phi}^{(d-1)}_{ik} - w^{d-1}_{ck} \bar{\phi}^{(d-1)}_{ic} \right)}. \tag{S27}$$

The optimal update for $\gamma^{(d)}_c$, $c \in [C]$ is

$$\gamma^{(d)}_c = \frac{\sum_{\mathbf{i}\in\Omega^{(d)}} \varphi^{(d)}_{\mathbf{i}c}}{\phi^{(d)}_c}, \tag{S28}$$

and the optimal update for $\gamma^{(d)}_k$, $k > C$ is

$$\gamma^{(d)}_k = \frac{\sum_{\mathbf{i}\in\Omega^{(d)}} \varphi^{(d)}_{\mathbf{i}k}}{\phi^{(d)}_k - \sum_{c=1}^{C} w^{d}_{ck} \phi^{(d)}_c}. \tag{S29}$$

Moreover,

$$\varphi_{ick} = \sum_{d=2}^{D} \sum_{\mathbf{i}\in\Omega^{(d)}} \varphi^{(d)}_{\mathbf{i}ick}, \tag{S30}$$

where

$$\varphi^{(d)}_{\mathbf{i}ick} = \varphi^{(d)}_{\mathbf{i}k} \cdot \frac{\theta_{ic} w_{ck} \left(\prod_{j\neq i} m_{jk} - w^{d-1}_{ck} \prod_{j\neq i} \theta_{jc} \right)}{\sum_{c'=1}^{C} w_{c'k}\theta_{ic'} \left(\prod_{j\neq i} m_{jk} - w^{d-1}_{c'k} \prod_{j\neq i} \theta_{jc'} \right)}, \tag{S31}$$

$$\varphi^{(d)}_{\mathbf{i}k} = A^{(d)}_{\mathbf{i}} \cdot \frac{\prod_{i\in\mathbf{i}} \mathbf{w}_{k'}^{\top}\boldsymbol{\theta}_i - 1(k > C) \sum_{c=1}^{C} w^{d}_{ck} \prod_{i\in\mathbf{i}} \theta_{ic}}{\sum_{k'=1}^{K} \left(\prod_{i\in\mathbf{i}} \mathbf{w}_{k'}^{\top}\boldsymbol{\theta}_i - 1(k' > C) \sum_{c=1}^{C} w^{d}_{ck'} \prod_{i\in\mathbf{i}} \theta_{ic} \right)}. \tag{S32}$$

Algorithm 2 EM algorithm for the omniassortative model.

Require: Adjacency tensors $\mathcal{A}^{(\cdot)}$, initialized parameters $\Gamma, \mathrm{W}, \Theta$, step size $\delta > 0$, stopping criterion.

repeat

E-step: $M \leftarrow \Theta\mathrm{W}$, compute the expected latent subcounts as in Eqs. (S30)–(S32)

$$\varphi^{(d)}_{\mathbf{i}k} \leftarrow A^{(d)}_{\mathbf{i}} \cdot \frac{\prod_{i\in\mathbf{i}} \mathbf{w}_{k'}^\top \boldsymbol{\theta}_i - 1(k>C)\sum_{c=1}^C w^d_{ck}\prod_{i\in\mathbf{i}}\theta_{ic}}{\sum_{k'=1}^K \left(\prod_{i\in\mathbf{i}} \mathbf{w}_{k'}^\top\boldsymbol{\theta}_i - 1(k'>C)\sum_{c=1}^C w^d_{ck'}\prod_{i\in\mathbf{i}}\theta_{ic}\right)},$$

$$\varphi^{(d)}_{\mathbf{i}ick} \leftarrow \frac{\theta_{ic}w_{ck}\left(\prod_{j\neq i} m_{jk} - w_{ck}^{d-1}\prod_{j\neq i}\theta_{jc}\right)}{\sum_{c'=1}^C w_{c'k}\theta_{ic'}\left(\prod_{j\neq i} m_{jk} - w_{c'k}^{d-1}\prod_{j\neq i}\theta_{jc'}\right)}\cdot \varphi^{(d)}_{\mathbf{i}k}, \quad \varphi_{ick} \leftarrow \sum_{d=2}^D \sum_{\mathbf{i}\in\Omega^{(d)}} \varphi^{(d)}_{\mathbf{i}ick}$$

M-step: update the parameters as in Eqs. (S27)–(S29)

$$\gamma^{(d)}_k \leftarrow \frac{\sum_{\mathbf{i}\in\Omega^{(d)}}\varphi^{(d)}_{\mathbf{i}k}}{\phi^{(d)}_k - \sum_{c=1}^C w^d_{ck}\phi^{(d)}_c}, \quad \gamma^{(d)}_c \leftarrow \frac{\sum_{\mathbf{i}\in\Omega^{(d)}}\varphi^{(d)}_{\mathbf{i}c}}{\phi^{(d)}_c}$$

$$\textbf{for } i\in[N] : \theta_{ic} \leftarrow \frac{\sum_{k=1}^K \varphi_{ick}}{\sum_{d=2}^D \gamma^{(d)}_c \bar{\phi}^{(d-1)}_{ic} + \sum_{k=C+1}^K \gamma^{(d)}_k w_{ck}\left(\bar{\phi}^{(d-1)}_{ik} - w^{d-1}_{ck}\bar{\phi}^{(d-1)}_{ic}\right)}$$

$$\text{update } \boldsymbol{\phi}, (\bar{\phi}^{(d)}_{i+1,k})_{d=2}^D$$

$$\boldsymbol{\nu} \leftarrow \boldsymbol{\nu} + \delta\nabla\mathcal{Q}(\mathrm{W}(\boldsymbol{\nu})), \quad \mathrm{W} \leftarrow \log(\exp(\boldsymbol{\nu})+1)$$

compute $\boldsymbol{\phi}, (\bar{\boldsymbol{\phi}}^{(d)})_{d=2}^D$

until stopping criterion met

return $\Gamma, \mathrm{W}, \Theta$

SUPPLEMENTARY NOTE 3: ADDITIONAL MATHEMATICAL DETAILS

Priors and MAP estimation

We may consider maximum a posteriori (MAP) estimation as an alternative to maximum likelihood estimation to infer the parameters. Here we provide a framework for this, by placing Gamma priors on the elements θ_{ic} of the node class-membership matrix and the community rate terms $\gamma^{(d)}_k$. The probability density function of $\gamma \sim \mathrm{Gamma}(\alpha,\beta)$ is given by

$$\mathrm{Gamma}(\gamma;\alpha,\beta) = \frac{\beta^\alpha}{\Gamma(\alpha)}\gamma^{\alpha-1}e^{-\beta\gamma}. \tag{S33}$$

In the case of independent and identically distributed $\theta_{ic} \overset{i.i.d.}{\sim} \mathrm{Gamma}(\alpha,\beta)$, the log likelihood is extended from $\mathcal{L}$ to $\mathcal{L} - (\alpha-1)\sum_{i,c}\log(\theta_{ic}) - \beta\sum_{i,c}\theta_{ic}$.

Under the maximum likelihood formulation, $\mathrm{y} \sim \mathrm{Poisson}(c\gamma)$, the optimal update is $\gamma = \frac{\mathrm{y}}{c}$ for scalar $c \in \mathbb{R}_{>0}$. Under the gamma prior $\gamma \sim \mathrm{Gamma}(\alpha,\beta)$, the optimal update is $\gamma = \max(\frac{\mathrm{y}+\alpha-1}{c+\beta}, 0)$. That is, we add $\alpha-1$ to the numerator and β to the denominator, and ensure the resulting update is non-negative. When $\alpha = 1$, the gamma distribution simplifies to the Exponential(β) distribution, and the optimal update is $\gamma = \frac{\mathrm{y}}{c+\beta}$. The exponential prior serves as a shrinkage prior, promoting sparse parameters, with shrinkage levels increasing with β. Increasing the sparsity via a suitable prior has the effect of driving the inferred memberships closer to hard membership vectors. We can apply these rules to the closed-form updates under each model. We note that such a parameterization removes the constraint that $||\boldsymbol{\theta}_c|| = 1$.

Graph (pairwise) setting

In the standard (pairwise) graph setting, all hyperedges are pairwise, i.e., $d{=}2$. Then

$$\mu_{ij}^{(d)} = \sum_{k=1}^{K} \gamma_k^{(2)} \sum_{c_1=1}^{C} \sum_{c_2=1}^{C} w_{c_1 k} w_{c_2 k} \theta_{ic_1} \theta_{ic_2} = \sum_{c_1=1}^{C} \sum_{c_2=1}^{C} \tilde{w}_{c_1 c_2} \theta_{ic_1} \theta_{ic_2} \,, \tag{S34}$$

where $\tilde{w}_{c_1c_2} := \sum_{k=1}^{K} \gamma_k^{(2)} w_{c_1k} w_{c_2k}$. We thus obtain a Tucker-2 decomposition (10), which has been used in various mixed-membership models for networks (11–14) to model disassortative community structure.

We can also find an alternative factorization, by writing the matrix $M = \Theta \mathrm{W}$ with entries $m_{ik} = \sum_{c=1}^{C} \theta_{ic} w_{ck}$. With this, we get

$$\begin{aligned} \mu_{ij}^{(d)} &= \sum_{k=1}^{K} \gamma_k^{(2)} \sum_{c_1=1}^{C} (\theta_{ic_1} w_{c_1 k}) \sum_{c_2=1}^{C} (\theta_{jc_2} w_{c_2 k}) \\ &= \sum_{k=1}^{K} \gamma_k^{(2)} (\boldsymbol{\theta}_i^\top \mathbf{w}_k)(\boldsymbol{\theta}_j^\top \mathbf{w}_k) = \sum_{k=1}^{K} \gamma_k^{(2)} m_{ik} m_{jk}. \end{aligned}$$

This is a symmetric non-negative matrix factorization that generalizes into a canonical polyadic (CP) decomposition (2, 10) if we consider $\boldsymbol{\gamma}^{(2)}$ to be the diagonal entries of a diagonal $K \times K$ affinity matrix. It is an assortative decomposition over the classes $[C]$, but allows for disassortativity over the nodes. In fact, writing $S = \mathrm{diag}(\boldsymbol{\gamma}^{(2)})$, $\upmu^{(:)} = MSM^\top = \Theta \mathrm{W} S \mathrm{W}^\top \Theta^\top \in \mathbb{R}^{N \times N}$, we obtain a bilinear model with affinity matrix $\tilde{\mathrm{W}} := \mathrm{W} S \mathrm{W}^\top$ as in Eq. (S34).

Properties of the Poisson

In our derivations we used two main properties of the Poisson distribution (15), as described below.

Poisson additivity. Let $y_k \overset{\text{ind.}}{\sim} \text{Poisson}(\lambda_k)$ for $k \in [K]$. Then marginally, the sum $y = \sum_{k=1}^{K} y_k \sim \text{Poisson}(\lambda)$ is also Poisson distributed, with rate $\lambda = \sum_{k=1}^{K} \lambda_k$.

Multinomial thinning. Let $y_k \overset{\text{ind.}}{\sim} \text{Poisson}(\lambda_k)$ for $k \in [K]$. Then conditional on the sum $y_\bullet = \sum_{k=1}^{K} y_k = n$, the vector $\boldsymbol{y} = (y_k)_{k=1}^{K}$ is distributed as

$$(\boldsymbol{y} \mid y_\bullet = n) \sim \begin{cases} \text{Multinomial}\,(n, \boldsymbol{\pi})\,, \ \text{where } \pi_k = \frac{\lambda_k}{\sum_{k'=1}^{K} \lambda_{k'}} & \text{if } n > 0 \\ \delta_{\mathbf{0}} & \text{otherwise.} \end{cases}$$

Computation

The updates to $\gamma_k^{(d)}$, θ_{ic} and w_{ck} require computing the sums over a combinatorial number of summands

$$\phi_k^{(d)} := \sum_{\mathbf{i}:|\mathbf{i}|=d} \prod_{r=1}^{d} \boldsymbol{\theta}_{i_r}^\top \mathbf{w}_k = \sum_{\mathbf{i}:|\mathbf{i}|=d} \prod_{r=1}^{d} m_{i_r k},$$

and

$$\bar{\phi}_{ik}^{(d)} := \sum_{\mathbf{i}:|\mathbf{i}|=d, i \notin \mathbf{i}} \prod_{r=1}^{d} \boldsymbol{\theta}_{i_r}^\top \mathbf{w}_k = \sum_{\mathbf{i}:|\mathbf{i}|=d, i \notin \mathbf{i}} \prod_{r=1}^{d} m_{i_r k}.$$

In particular, each $\phi_k^{(d)}$ contains $\binom{N}{d}$ summands and each $\bar{\phi}_{ik}^{(d)}$ contains $\binom{N-1}{d}$ summands. Since the optimal θ_{ic} depends on the values of $\theta_{i'c'}$ for other nodes, updates to θ_{ic} are performed sequentially. Each update to θ_{ic} changes $\phi_{i'k}^{(d)}$ for $i' \neq i$, necessitating a recalculation of $\bar{\phi}_{ik}^{(d)}$ for each i in a sequential manner. We use the algorithm derived in (9) to compute each term in O(1) time, which conditional on the previous values, updates $\phi_k^{(d)}$ according to an update rule.

The updates to W, however, cannot rely on this algorithm to compute $\phi_k^{(d)}$, as the automatic differentiation call requires a function which computes $\phi_k^{(d)}$ as functions of Θ and W from scratch. Therefore, we derive a dynamic

programming algorithm to compute $\phi_k^{(d)}$ and $\bar{\phi}_{ik}^{(d)}$ efficiently. Letting $\bar{\phi}_{ik}^{(0)} = 1$, it holds that

$$\phi_k^{(d)} = \frac{1}{d}\sum_{i=1}^{N} m_{ik}\bar{\phi}_{ik}^{(d-1)}, \quad \bar{\phi}_{ik}^{(d)} = \phi_k^{(d)} - m_{ik}\bar{\phi}_{ik}^{(d-1)} \quad \text{for } d \in [D]. \tag{S35}$$

Proof. The result relies on the following proposition.
Proposition. As defined in Eq. (S35), $\bar{\phi}_{ik}^{(d-1)}$ may be equivalently expressed as $\bar{\phi}_{ik}^{(d-1)} = \sum_{\mathbf{i}\in\Omega^{(d)}, i\in\mathbf{i}} \prod_{j\neq i} m_{jk}$.

Proof. Let

$$\Omega_i^{(d)} = \{\mathbf{i} \in \Omega^{(d)} : i \in \mathbf{i}\}, \quad \bar{\Omega}_i^{(d)} = \{\mathbf{i} \in \Omega^{(d)} : i \notin \mathbf{i}\}.$$

Define the bijection

$$f_i : \bar{\Omega}_i^{(d-1)} \to \Omega_i^{(d)}, \quad f_i(\mathbf{i}) = \mathbf{i} \cup \{i\}.$$

Since f_i is bijective, we have

$$|\bar{\Omega}_i^{(d-1)}| = |\Omega_i^{(d)}|.$$

Moreover, for any $\mathbf{i} \in \bar{\Omega}_i^{(d-1)}$:

$$\prod_{j\in\mathbf{i}} m_{jk} = \prod_{j\in f_i(\mathbf{i}), j\neq i} m_{jk},$$

since $f_i(\mathbf{i}) \setminus \{i\} = \mathbf{i} \cup \{i\} \setminus \{i\} = \mathbf{i}$.
Therefore,

$$\begin{aligned}\bar{\phi}_{ik}^{(d-1)} &:= \sum_{\mathbf{i}\in\Omega^{(d-1)}, i\notin\mathbf{i}} \prod_{j\in\mathbf{i}} m_{jk} = \sum_{\mathbf{i}\in\Omega^{(\bar{d}-1)}{}_i} \prod_{j\in\mathbf{i}} m_{jk} = \sum_{\mathbf{i}\in\Omega^{(\bar{d}-1)}{}_i} \prod_{j\in f_i(\mathbf{i}), j\neq i} m_{jk} \\ &= \sum_{f_i(\mathbf{i})\in\Omega_i^{(d)}} \prod_{j\in f_i(\mathbf{i}), j\neq i} m_{jk} = \sum_{\boldsymbol{u}\in\Omega_i^{(d)}} \prod_{j\in\boldsymbol{u}, j\neq i} m_{jk} \\ &= \sum_{\boldsymbol{u}\in\Omega^{(d)}, i\in\boldsymbol{u}} \prod_{j\neq i} m_{jk}.\end{aligned}$$

□

Proof of first relation. By definition,

$$\phi_k^{(d)} = \sum_{\mathbf{i}\in\Omega^{(d)}} \prod_{i_r=1}^{d} m_{i_r k} \tag{S36}$$

$$= \sum_{\mathbf{i}\in\Omega^{(d)}} \frac{1}{d}\sum_{r=1}^{d} \prod_{i_{r'}=1}^{d} m_{i_{r'} k} \tag{S37}$$

$$= \frac{1}{d}\sum_{\mathbf{i}\in\Omega^{(d)}} \sum_{r=1}^{d} m_{i_r k} \prod_{r'\neq r} m_{i_{r'} k}. \tag{S38}$$

Since each node i occurs in each hyperedge $\mathbf{i}$ at most once,

$$\sum_{r=1}^{d} m_{i_r k} \prod_{m'\neq m} m_{i'_r k} = \sum_{r=1}^{d}\sum_{i=1}^{N} 1(i_r = i) m_{ik} \prod_{j\in\mathbf{i}, j\neq i} m_{jk} \tag{S39}$$

$$= \sum_{i=1}^{N} 1(i \in \mathbf{i}) m_{ik} \prod_{j\in\mathbf{i}, j\neq i} m_{jk}. \tag{S40}$$

Plugging Eq. (S40) into Eq. (S38) yields

$$\begin{aligned}\phi_k^{(d)} &= \frac{1}{d}\sum_{i=1}^{N} m_{ik} \sum_{\mathbf{i}\in\Omega^{(d)}, i\in\mathbf{i}} \prod_{j\in\mathbf{i}, j\neq i} m_{jk} \\ &= \frac{1}{d}\sum_{i=1}^{N} m_{ik} \sum_{\mathbf{i}\in\Omega^{(d-1)}, i\notin\mathbf{i}} \prod_{j\in\mathbf{i}} m_{jk} \\ &= \frac{1}{d}\sum_{i=1}^{N} m_{ik}\bar{\phi}_{ik}^{(d-1)}.\end{aligned}$$

Proof of second relation. For $i \in [N]$, we partition $\Omega^{(d)}$ into two sets, based on whether a hyperedge contains node i.

$$\Omega^{(d)} = \{\mathbf{i} \in \Omega^{(d)} : i \in \mathbf{i}\} \cup \{\mathbf{i} \in \Omega^{(d)} : i \notin \mathbf{i}\}. \tag{S41}$$

Then the sum over all hyperedges of order d expands as

$$\begin{aligned}\phi_k^{(d)} = \sum_{\mathbf{i}\in\Omega^{(d)}} \prod_{r=1}^{d} m_{i_r k} &= \sum_{\mathbf{i}\in\Omega^{(d)}: i\in\mathbf{i}} \prod_{r=1}^{d} m_{i_r k} + \sum_{\mathbf{i}\in\Omega^{(d)}: i\notin\mathbf{i}} \prod_{r=1}^{d} m_{i_r k} \\ &= m_{ik}\phi_{ik}^{(d-1)} + \bar{\phi}_{ik}^{(d)},\end{aligned}$$

implying that $\bar{\phi}_{ik}^{(d)} = \phi_k^{(d)} - m_{ik}\bar{\phi}_{ik}^{(d-1)}$. □

Computation scales as $\mathrm{O}(NDK)$, where $\phi_{ik}^{(d)}$ can be computed in parallel over i, k and $\phi_k^{(d)}$ in parallel over k.

SUPPLEMENTARY NOTE 4: EXPERIMENTAL DETAILS

Initialization. In our experiments, we initialize the main parameters as:

$$\begin{aligned} \boldsymbol{\theta}_i &\overset{\text{i.i.d.}}{\sim} \text{Dirichlet}(10^3, \ldots, 10^3) && \forall i \in [N] \\ \mathbf{w}_k &\overset{\text{i.i.d.}}{\sim} \text{Dirichlet}(1, \ldots, 1) && \text{for } k > C \\ \gamma_k^{(d)} &= 1 && \forall d \in \{2, \ldots, D\}, k \in [K]. \end{aligned}$$

To promote the learning of disassortative structure in the experiments in the hospital data setting, we initialize $\gamma_k^{(d)} = 0.01$ for $k \leq C$ and $\gamma_k^{(d)} = 1$ otherwise.

Training. All of our experiments were run using one CPU. Convergence was assessed by monitoring the log-likelihood given in Eq. (S17). We trained each model until its value changed by less than 1 over 10 iterations, or 1,000 iterations, whichever occurred first. The models which require a learning rate were trained with a learning rate of 10^{-6}. We do not update the first C columns of W during gradient ascent to preserve the structure $\mathrm{W} = [\mathrm{I}_C \mid \mathbf{w}_{c+1}, \ldots, \mathbf{w}_K]$.

While larger choices of K add computational cost to fitting each model, we find that it is better to overestimate K than underestimate when evaluating results. In particular, we find that if a community contains no meaningful structure, the model learns to set $\gamma_k^{(d)}$ very close to zero for each d.

Post-training transformation. Each generalized EM algorithm converges to yield estimates $\Gamma, \Theta, \mathrm{W}$. These estimates are not ensured to fall into the constrained model class. Therefore, define the constants and parameters $\psi_c = \sum_{i=1}^{N} \theta_{ic}$, $\tilde{\theta}_{ic} = \frac{\theta_{ic}}{\psi_c}$, $\psi_k = \sum_{c=1}^{C} w_{ck}\psi_c$, $\tilde{w}_{ck} = \frac{w_{ck}\psi_c}{\psi_k}$, $\tilde{\gamma}_k^{(d)} = \gamma_k^{(d)}(\psi_k)^d$. Then $(\tilde{\Gamma}, \tilde{\Theta}, \tilde{\mathrm{W}})$ belongs in the constrained

model class. Moreover,

$$\begin{aligned}\tilde{\mu}^{(d)}_{i_1\ldots i_d} &= \sum_{k=1}^{K} \tilde{\gamma}^{(d)}_k \prod_{r=1}^{d} \left(\sum_{c=1}^{C} \tilde{w}_{ck} \tilde{\theta}_{ic} \right) \\ &= \sum_{k=1}^{K} \gamma^{(d)}_k (\psi_k)^d \prod_{r=1}^{d} \left(\sum_{c=1}^{C} \frac{w_{ck}\psi_c\theta_{ic}}{\psi_c\psi_k} \right) \\ &= \sum_{k=1}^{K} \gamma^{(d)}_k \prod_{r=1}^{d} \left(\sum_{c=1}^{C} w_{ck}\theta_{ic} \right) = \mu^{(d)}_{i_1\ldots i_d}.\end{aligned}$$

That is, we may fit the unconstrained model and transform the parameters accordingly only once at the end, after convergence is achieved. Doing so makes them more interpretable and ensures they lie in the identifiable model class. In practice, however, we find that applying these transformations at each iteration improves the numerical stability of the algorithm.

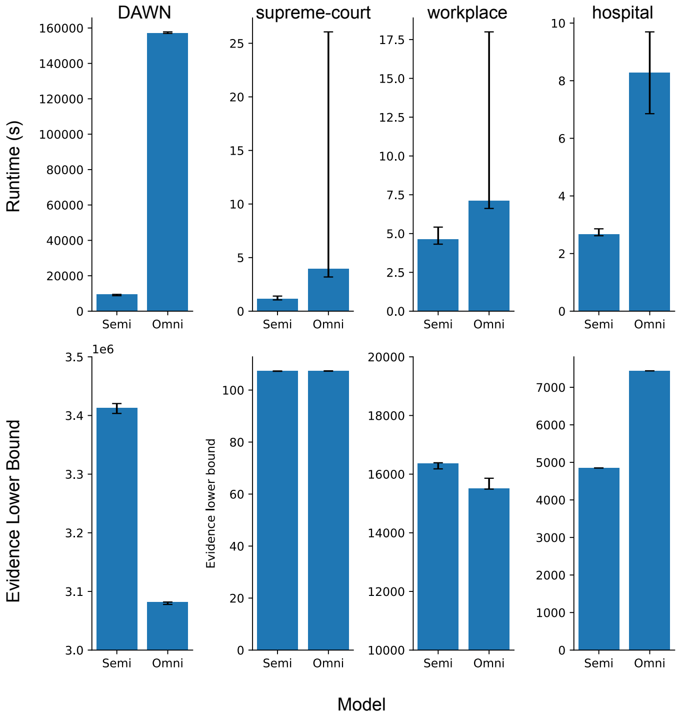

Supplementary Figure 1. **The tradeoff between computational cost and model expressivity varies by dataset.** Top) Median runtime of performing a full run of the algorithm for $R = 10$ random initialization restarts, measured in seconds. We use the same combination of C and K for both models. Bottom) Median Evidence Lower Bound $\mathcal{Q}$ as defined in Eq. (S18); higher values imply better performance. For the DAWN dataset we see how the semi-assortative model performs significantly better both in terms of lower runtimes and better $\mathcal{Q}$. Instead, the hospital dataset shows a trade-off where the omni-assortative variant has better $\mathcal{Q}$ at the cost of higher runtime. In both settings, error bars represent the interquartile range.

Drug case study: selection criteria.

For $C, K \in (8, 16, 32, 48, 64)$ and $C \leq K$, we fit the omniassortative model to the data, masking a subset of the data and select the C and K that achieves the highest heldout log-likelihood, which occurs for $C = 16$ and $K = 48$ (approximately the best combination of C and K).

To measure how much a community differs from the set of classes, we compute the Jensen-Shannon divergence (16) from its nearest class, where larger values imply greater values of disassortativity. During inference, our algorithm allocates observed hyperedges to communities and clusters. We can understand the number of hyperedges allocated to a given community as a measure of its importance (17, 18). Communities with larger assigned latent counts account for more of the observed data.

We select two communities with large dissimilarity measures (as measured by Jensen-Shannon divergence to its nearest class) that are assigned at least $1,000$ hyperedge counts (see Methods: allocation). These communities

correspond to $k = 19$ and $k = 44$, shown in the main paper. We use these two communities and their corresponding classes to identify related classes and communities (classes are shown in maroon, communities in bronze). The term w_{ck} for class c and community k is proportional to the width of the edge between class c and community k.

Runtime comparisons. Here, we compare model runtime and performance, as measured by the evidence lower bound (ELBO) $\mathcal{Q}$ as defined in Eq. (S18), for the omni-assortative and semi-assortative model variants. In Supplementary Figure 1 we show how the trade-off between runtime and performance varies by dataset. While the semi-assortative model achieves a better runtime and ELBO on the DAWN data, the omni-assortative model achieves a better fit to the hospital data, at the cost of extra runtime.

Sensitivity to small perturbations in the data. We have run a stability assessment to ensure that the inferred mesoscale structures are robust. For this, we have run a perturbation experiment on the Supreme Court and hospital datasets, where we removed a random 10% of all hyperedges and measure the similarity of the community assignments resulting from the different perturbed datasets. Specifically, we measure the cosine similarity between communities inferred from the whole dataset and from the dataset partially pruned and find the communities align very closely to one another. The cosine similarity is measured between 0 and 1, where 1 represents identical membership assignment. We find nearly identical community structure learned from the full and ablated datasets. For each dataset, we observe a mean cosine similarity greater than 0.99 with little variation across seeds (standard deviation less than 0.005).

Hypergraph generation: inclusion occurrences. The inclusion occurrence of a hyperedge size d is the number of nonzero hyperedges of size d which appear as a subset of a hyperedge of size $d+1$. Computing the inclusion occurrences of the hypergraph we generate is expensive due to the size of the hypergraph. Therefore, for each hyperedge order, we randomly sample 10^4 hyperedges to form a smaller hypergraph and compute its inclusion occurrences. We repeat this procedure 10 times, for both the true and synthetic hypergraph data and show the average.

SUPPLEMENTARY NOTE 5: DETAILED DERIVATIONS

In Supplementary Note 2, we give the updates and the general framework for how we derive them. Here, we explicitly derive them in more detail. Specifically, we give full derivations of the optimal updates for the semi-assortative model. The derivations of the fully omniassortative updates are similar to the derivations here—the derivations for the strictly assortative derivations are given by (9). Abusing notation, we write $\mathcal{Q}$ to mean $\mathcal{Q}(\mathcal{A}^{(\cdot)}, \Gamma, \mathrm{W}, \Theta)$.

The evidence lower bound is given by:

$$\mathcal{Q} = -\sum_{d=2}^{D}\sum_{k=1}^{K}\gamma_k^{(d)}\phi_k^{(d)} + \sum_{d=2}^{D}\sum_{k=1}^{K}\sum_{\mathbf{i}\in\Omega^{(d)}}\mathbb{E}\left[A_{\mathbf{i}k}^{(d)} \mid \mathcal{A}^{(\cdot)}\right]\log(\gamma_k^{(d)}) + \sum_{d=2}^{D}\sum_{k=1}^{K}\sum_{\mathbf{i}\in\Omega^{(d)}}\sum_{r=1}^{d}\sum_{c_r=1}^{C}\mathbb{E}\left[A_{\mathbf{i}i_r c_r k}^{(d)} \mid \mathcal{A}^{(\cdot)}\right]\log(\theta_{i_r c_r} w_{c_r k}) \tag{S42}$$

$$= -\sum_{d=2}^{D}\sum_{k=1}^{K}\gamma_k^{(d)}\phi_k^{(d)} + \sum_{d=2}^{D}\sum_{k=1}^{K}\sum_{\mathbf{i}\in\Omega^{(d)}}\mathbb{E}\left[A_{\mathbf{i}k}^{(d)} \mid \mathcal{A}^{(\cdot)}\right]\log(\gamma_k^{(d)}) + \sum_{k=1}^{K}\sum_{i=1}^{N}1(i\in\mathbf{i})\sum_{c=1}^{C}\sum_{d=2}^{D}\sum_{\mathbf{i}\in\Omega^{(d)}}\mathbb{E}\left[A_{\mathbf{i}ick}^{(d)} \mid \mathcal{A}^{(\cdot)}\right]\log(\theta_{ic} w_{kc}) \tag{S43}$$

$$= -\sum_{d=2}^{D}\sum_{k=1}^{K}\gamma_k^{(d)}\phi_k^{(d)} + \sum_{d=2}^{D}\sum_{k=1}^{K}\sum_{\mathbf{i}\in\Omega^{(d)}}\mathbb{E}\left[A_{\mathbf{i}k}^{(d)} \mid \mathcal{A}^{(\cdot)}\right]\log(\gamma_k^{(d)}) + \sum_{k=1}^{K}\sum_{i=1}^{N}\sum_{c=1}^{C}\mathbb{E}\left[A_{ick}^{(d)} \mid \mathcal{A}^{(\cdot)}\right]\log(\theta_{ic} w_{kc}) \tag{S44}$$

$$= -\sum_{d=2}^{D}\sum_{k=1}^{K}\gamma_k^{(d)}\phi_k^{(d)} + \sum_{d=2}^{D}\sum_{k=1}^{K}\sum_{\mathbf{i}\in\Omega^{(d)}}\mathbb{E}\left[A_{\mathbf{i}k}^{(d)} \mid \mathcal{A}^{(\cdot)}\right]\log(\gamma_k^{(d)}) + \sum_{i=1}^{N}\sum_{c=1}^{C}\log(\theta_{ic})\sum_{k=1}^{K}\mathbb{E}\left[A_{ick}^{(d)} \mid \mathcal{A}^{(\cdot)}\right] \tag{S45}$$

$$+\sum_{k=1}^{K}\sum_{c=1}^{C}\log(w_{ck})\sum_{i=1}^{N}\mathbb{E}\left[A_{ick}^{(d)} \mid \mathcal{A}^{(\cdot)}\right]. \tag{S46}$$

M-step.

Update to $\boldsymbol{\theta_{ic}}$. To update θ_{ic}, we set the partial derivative $\frac{\partial\mathcal{Q}}{\partial\theta_{ic}} = 0$ and solve for θ_{ic}. By definition,

$$\frac{\partial m_{ik}}{\partial\theta_{ic}} = w_{ck}, \quad \frac{\partial\phi_k^{(d)}}{\partial m_{ik}} = \sum_{\mathbf{i}\in\Omega^{(d)}, i\in\mathbf{i}}\prod_{i_r\neq i} m_{i_r k} := \bar{\phi}_{ik}^{(d-1)}.$$

The partial derivative is given by:

$$\begin{aligned}\frac{\partial \mathcal{Q}}{\partial \theta_{ic}} &= -\sum_{d=2}^{D}\sum_{k=1}^{K}\gamma_k^{(d)}\left(\frac{\partial \phi_k^{(d)}}{\partial m_{ik}}\cdot\frac{\partial m_{ik}}{\partial \theta_{ic}}\right)+\frac{1}{\theta_{ic}}\sum_{k=1}^{K}\mathbb{E}[A_{ick}^{(d)} \mid \mathcal{A}^{(:)}] \\ &= \sum_{d=2}^{D}\sum_{k=1}^{K}\gamma_k^{(d)}\bar{\phi}_{ik}^{(d-1)}w_{ck}+\frac{1}{\theta_{ic}}\sum_{k=1}^{K}\mathbb{E}[A_{ick}^{(d)} \mid \mathcal{A}^{(:)}].\end{aligned}$$

Setting $\frac{\partial \mathcal{Q}}{\partial \theta_{ic}} = 0$ and solving for θ_{ic} yields

$$\theta_{ic} = \frac{\sum_{k=1}^{K}\mathbb{E}[A_{ick}^{(d)} \mid \mathcal{A}^{(:)}]}{\sum_{d=2}^{D}\sum_{k=1}^{K}\gamma_k^{(d)}\bar{\phi}_{ik}^{(d-1)}w_{ck}}.$$

Update to $\gamma_k^{(d)}$. The partial derivative $\frac{\partial Q}{\partial \gamma_k^{(d)}}$ is given by:

$$\frac{\partial Q}{\partial \gamma_k^{(d)}} = -\phi_k^{(d)} + \frac{1}{\gamma_k^{(d)}}\sum_{\mathbf{i}\in\Omega^{(d)}}\mathbb{E}\left[A_{\mathbf{i}k}^{(d)} \mid \mathcal{A}^{(:)}\right].$$

Setting $\frac{\partial Q}{\partial \gamma_k^{(d)}} = 0$ and solving for $\gamma_k^{(d)}$ yields:

$$\gamma_k^{(d)} = \frac{\sum_{\mathbf{i}\in\Omega^{(d)}}\mathbb{E}\left[A_{\mathbf{i}k}^{(d)} \mid \mathcal{A}^{(:)}\right]}{\phi_k^{(d)}}.$$

E-step. The updates require computing $\varphi_{\mathbf{i}k}^{(d)} := \mathbb{E}[A_{\mathbf{i}k}^{(d)} \mid \mathcal{A}^{(:)}]$ and $\varphi_{\mathbf{i}ick}^{(d)} := \mathbb{E}[A_{\mathbf{i}ick}^{(d)} \mid \mathcal{A}^{(:)}]$. By multinomial thinning,

$$A_{i_1\dots i_d k}^{(d)} \mid A_{i_1\dots i_d}^{(d)} = a \sim \text{Multinomial}\left(a, \frac{\gamma_k^{(d)}\prod_{r=1}^{d} m_{i_r k}}{\sum_{k'=1}^{K}\gamma_{k'}^{(d)}\prod_{r=1}^{d} m_{i_r k'}}\right).$$

Therefore,

$$\mathbb{E}[A_{\mathbf{i}k}^{(d)} \mid \mathcal{A}^{(:)}] = \mathbb{E}[A_{\mathbf{i}k}^{(d)} \mid A_{\mathbf{i}}^{(d)}] = A_{\mathbf{i}}^{(d)}\cdot\frac{\gamma_k^{(d)}\prod_{r=1}^{d} m_{i_r k}}{\sum_{k'=1}^{K}\gamma_{k'}^{(d)}\prod_{r=1}^{d} m_{i_r k'}}.$$

To compute $\mathbb{E}[A_{\mathbf{i}ick}^{(d)} \mid \mathcal{A}^{(:)}]$, we use the law of total expectation.

$$\begin{aligned}\mathbb{E}[A_{\mathbf{i}ick}^{(d)} \mid \mathcal{A}^{(:)}] &= \mathbb{E}[\mathbb{E}[A_{\mathbf{i}ick}^{(d)} \mid A_{\mathbf{i}k}^{(d)}, A_{\mathbf{i}}^{(d)}] \mid A_{\mathbf{i}}^{(d)}] \\ &= \mathbb{E}[\mathbb{E}[A_{\mathbf{i}ick}^{(d)} \mid A_{\mathbf{i}k}^{(d)}] \mid A_{\mathbf{i}}^{(d)}].\end{aligned}$$

$(A_{\mathbf{i}ick}^{(d)})_{c=1}^{C} \mid A_{\mathbf{i}k}^{(d)} \sim \text{Multinomial}\left(A_{\mathbf{i}k}^{(d)}, \frac{\theta_{ic}w_{ck}}{\sum_{c'=1}^{C}\theta_{ic'}w_{kc'}}\right)$. As such,

$$\mathbb{E}[A_{\mathbf{i}ick}^{(d)} \mid \mathcal{A}^{(:)}] = \mathbb{E}[A_{\mathbf{i}k}^{(d)} \mid A_{\mathbf{i}}^{(d)}]\cdot\frac{\theta_{ic}w_{ck}}{\sum_{c'=1}^{C}\theta_{ic'}w_{c'k}}.$$

We compute expectations in a compositional manner, first computing $\mathbb{E}[A_{\mathbf{i}k}^{(d)} \mid A_{\mathbf{i}}^{(d)}]$, and then $\mathbb{E}[A_{\mathbf{i}ick}^{(d)} \mid A_{\mathbf{i}}^{(d)}]$ conditional on $\mathbb{E}[A_{\mathbf{i}k}^{(d)} \mid A_{\mathbf{i}}^{(d)}]$.

REFERENCES

[1] N. Gillis, *Nonnegative Matrix Factorization* (SIAM, 2020).

[2] F. L. Hitchcock, The expression of a tensor or a polyadic as a sum of products. *Journal of Mathematics and Physics* **6**, 164–189 (1927).

[3] N. D. Sidiropoulos, R. Bro, On the uniqueness of multilinear decomposition of n-way arrays. *Journal of Chemometrics: A Journal of the Chemometrics Society* **14**, 229–239 (2000).

[4] A. Ben-Israel, T. N. Greville, *Generalized inverses: theory and applications* (Springer Science & Business Media, 2006).
[5] J. B. Kruskal, Rank, decomposition, and uniqueness for 3-way and n-way arrays. *Multiway data analysis* (1989), pp. 7–18.
[6] A. P. Dempster, N. M. Laird, D. B. Rubin, Maximum likelihood from incomplete data via the em algorithm. *Journal of the Royal Statistical Society: Series B (Methodological)* **39**, 1–22 (1977).
[7] C. J. Wu, On the convergence properties of the em algorithm. *The Annals of statistics* pp. 95–103 (1983).
[8] A. G. Baydin, B. A. Pearlmutter, A. A. Radul, J. M. Siskind, Automatic differentiation in machine learning: a survey. *Journal of machine learning research* **18**, 1–43 (2018).
[9] M. Contisciani, F. Battiston, C. De Bacco, Inference of hyperedges and overlapping communities in hypergraphs. *Nature Communications* **13**, 7229 (2022).
[10] T. G. Kolda, B. W. Bader, Tensor Decompositions and Applications. *SIAM Review* **51**, 455–500 (2009).
[11] B. Ball, B. Karrer, M. E. Newman, Efficient and principled method for detecting communities in networks. *Physical Review E* **84**, 036103 (2011).
[12] C. De Bacco, E. A. Power, D. B. Larremore, C. Moore, Community detection, link prediction, and layer interdependence in multilayer networks. *Physical Review E* **95**, 042317 (2017).
[13] A. Schein, M. Zhou, D. M. Blei, H. Wallach, *Proceedings of the 33rd International Conference on Machine Learning* (2016), pp. 2810–2819.
[14] M. Contisciani, E. A. Power, C. De Bacco, Community detection with node attributes in multilayer networks. *Scientific reports* **10**, 1–16 (2020).
[15] J. F. C. Kingman, *Poisson processes*, vol. 3 (Clarendon Press, 1992).
[16] J. Lin, Divergence measures based on the shannon entropy. *IEEE Transactions on Information theory* **37**, 145–151 (1991).
[17] A. Schein, Allocative Poisson factorization for computational social science (2019).
[18] S. Yıldırım, M. B. Kurutmaz, M. Barsbey, U. Şimşekli, A. T. Cemgil, Bayesian allocation model: marginal likelihood-based model selection for count tensors. *IEEE Journal of Selected Topics in Signal Processing* **15**, 560–573 (2020).
