# Supplementary material for: Broad spectrum structure discovery in large-scale higher-order networks
Source: Nat Commun. 2026 Apr 27;17:5765. doi: 10.1038/s41467-026-71903-0 (PMC13324705; doi:10.1038/s41467-026-71903-0)
Supplement: Supplementary file 2 — Transparent Peer Review file [file 41467_2026_71903_MOESM2_ESM.pdf]

nature portfolio

Peer Review File

Broad Spectrum Structure Discovery in Large-Scale Higher-Order Networks

Corresponding Author: Dr Caterina De Bacco

This file contains all reviewer reports in order by version, followed by all author rebuttals in order by version.

Version 0:

Reviewer comments:

Reviewer #1

(Remarks to the Author)
The manuscript "Broad Spectrum Structure Discovery in Large-Scale Higher-Order Networks" by J. Hood and co-authors introduces a versatile probabilistic framework for hypergraphs that captures mesoscale structures, from strictly assortative communities to disassortative class–interaction patterns. By treating latent classes as nodes in an auxiliary hypergraph and imposing a low-rank factorization on class–class affinities, the method remains scalable and yields interpretable node- and class-level structures. Empirical results on pharmacological and social link-prediction benchmarks show clear improvements over existing approaches. Altogether, this is an important contribution to the literature, and I believe it will be of wide interest to researchers using higher-order networks across fields. Overall, the paper is well-written and clear. However, I have some comments and suggestions before recommending it for publication. Please find below a detailed list of my comments:

- The manuscript would benefit from clear guidance on selecting the number of communities and classes. If I understood correctly, it seems that grid search was used throughout the manuscript to obtain specific choices for each dataset. While cross-validation is mentioned, incorporating a possible model-selection criterion or presenting a sensitivity analysis over different values would help practitioners balance under- and over-clustering and make the approach easier to use.

- To ensure the inferred mesoscale structures are robust, it could be worth including a comprehensive stability assessment. For example, report the variability in community assignments and link-prediction metrics across a larger set of random initializations (beyond the ten runs currently noted), and/or conduct a perturbation experiment—such as removing 5–10 % of hyperedges—to confirm that results remain consistent under data changes. These analyses will demonstrate that the detected patterns reflect genuine structure rather than artifacts of particular seeds/noise.

- In the manuscript, the authors highlight that the omni-assortative variant is the most expressive (but also the most expensive), while the semi-assortative model strikes a balance and is used in most experiments. To elucidate the trade-offs between expressivity and computational cost, I recommend including a few comparisons of the semi-assortative and omni-assortative models on a selection of synthetic/real-world datasets. Such side-by-side benchmarks would clarify each variant's relative performance, runtime, and suitability for different applications.

- The description of critical implementation choices—such as EM initialization strategies, convergence thresholds, and stopping criteria—is currently spread across the Methods and Supplementary sections, which makes reproduction challenging. I recommend adding a concise pseudocode box or flowchart in the main Methods that clearly outlines each inference step, lists key hyperparameters, and specifies used criteria for convergence and termination.

- In addition to the previous comment, it is crucial that the authors provide a well-documented code on Github. The current repository contains unannotated Julia scripts with no usage examples, making them difficult to use. I strongly recommend releasing a well-commented version of the code that clearly exposes each model variant, includes example notebooks on synthetic and real datasets, and perhaps reproduces core figures from the manuscript. This will greatly enhance reproducibility and adoption.

- While the current benchmarks on pharmacological and social datasets are compelling, including an additional domain—such as co-authorship hypergraphs—would further demonstrate the method's versatility and broaden its appeal.

Minor comments:
- Ensure all figure panels and supplementary notes are numbered in the order they appear.
- In Figure 5, choose more distinguishable color palettes and include error bars (or clarify deterministic vs. stochastic behavior) for the omni-assortative results.
- It's not clear whether the statement "The omniassortative model achieves lower H(Θ) values for five of the six datasets (senate-bills, senate-committees, NDC-substances, DAWN, and workplace) while for contact-high-school, the values are similar." is supported by any hypothesis tests or significance (e.g., error bars in some cases should not support the claim)

(Remarks on code availability)
The authors must provide a well-documented code on Github. The current GitHub repository contains unannotated Julia scripts with no usage examples or code for reproducing existing figures. I strongly recommend releasing a well-commented version of the code that exposes each model variant, includes example notebooks on synthetic and real datasets, and perhaps reproduces core figures from the manuscript. This will greatly enhance reproducibility and adoption.

Reviewer #2

(Remarks to the Author)
I co-reviewed this manuscript with one of the reviewers who provided the listed reports. This is part of the Nature Communications initiative to facilitate training in peer review and to provide appropriate recognition for Early Career Researchers who co-review manuscripts.

(Remarks on code availability)

Reviewer #3

(Remarks to the Author)
The paper introduces a family of probabilistic generative models aimed at discovering and interpreting mesoscale structures in large-scale hypergraphs. The authors use a framework that jointly clusters nodes into latent classes and classes into communities, using low-rank tensor factorizations to ensure scalability and interpretability. They applied the framework to study some
real-world datasets.

I believe that the study is technically correct and, although it uses known techniques, their combination produces novel results. Also, the problem that is investigated is worth looking at. Therefore, I believe this contribution has the potential to be considered for publication in Nat Comm. However, it is not acceptable in its present form for the reasons listed below (in no particular order):

- I believe that the generality of the current framework is not discussed enough. The authors certainly present "success cases", but I don't see a way to prove that their method is practical for large datasets or that the use of low-rank factorizations and variational inference allows for tractable estimation on high-order, large-scale hypergraphs. More details on these or a deeper discussion would improve the potential impact and general appeal of the study.

- The validation using real-world data is somewhat limited: While the datasets used are diverse, more domain-specific insights would strengthen claims of interpretability.

- Please, provide more evidence regarding statements of scalability. In the current version, not enough quantitative indicators of performance are provided. Otherwise, some statements should be significantly toned down.

- The paper is hard to follow in several parts as the notation is dense. It could benefit from a "running example" to ground the concepts. Also, for the sake of reaching a wider audience, a bit more of (even if trivial) explanations regarding the use of multiple indices and latent variables (e.g., nodes, classes, communities) could be helpful to avoid confusion of readers unfamiliar with tensor-based models. I particularly like the approach, but the subset of network scientists used to work with tensorial representations is limited, and so could be the audience if the text is not simplified a bit or explained in more detail. For instance, a figure with a model diagram and a step-by-step illustration of the generative process could help.

- The assumption of low-rank structure in class interactions might be unrealistic for certain hypergraphs with high local complexity. Please, add a discussion of the limitations or alternative extensions for more complex hypergraph topologies.

Once the above comments and suggestions have been thoroughly addressed, the paper could be reconsidered for publication.

(Remarks on code availability)

Reviewer #4

(Remarks to the Author)
This paper introduces a principled and scalable framework for uncovering mesoscale structure in large-scale hypergraphs, networks capturing polyadic interactions. The Authors propose a probabilistic model that combines latent node classes with low-rank tensor decomposition to extract interpretable patterns of interaction.
The framework generalizes classical stochastic block models to higher-order data, enables structure discovery beyond traditional graph-based techniques, and is applicable to a broad range of domains, including social networks, tagging systems, and pharmacological interactions.
The model is evaluated on several large, real-world datasets and consistently outperforms state-of-the-art baselines on link prediction tasks while also offering highly interpretable structural insights.

In general the manuscript is clearly written, well-organized, and supported by informative and well-labeled figures.
The introduction of latent-class modeling for hypergraphs fills a notable gap in the field. The approach bridges the strengths of the stochastic block modeling approaches with modern tensor techniques.
The Authors provide a variational expectation maximization algorithm capable of handling datasets with millions of nodes and hyperedges, making the method suitable for large-scale applications.
Beyond prediction, the proposed model gives meaningful structural insights via latent hypergraphs and class-role visualizations.
A thorough evaluation on multiple datasets, two biological datasets, three human-contact networks, and three political datasets, showcases strong performance in link prediction and interpretable structure discovery.
In the following some comments and suggestions:
1. While theoretical identifiability is discussed, practical convergence behavior and empirical stability of the variational expectation maximization algorithm are not deeply addressed. Including convergence plots or runtime benchmarks would strengthen this section.
2. The manuscript could benefit from ablation experiments comparing hard vs. soft class assignments or evaluating the role of multi-membership in predictive accuracy.
3. In addition to link prediction, metrics such as modularity, normalized mutual information, or adjusted rand index could be used to assess how well the learned classes align with known structures (when available).
4. Clarify whether hyperedge sizes are assumed to be uniform or variable.
5. A brief discussion of computational complexity, especially for large-rank tensors, would be helpful.
6. Reporting standard deviations or confidence intervals in performance metrics would improve result robustness.
The manuscript presents a novel, well-executed, and broadly applicable framework for structure discovery in higher-order networks. The methodological rigor, scalability, and interpretability of the approach—coupled with convincing empirical results—make this paper a valuable contribution for network science communities.

(Remarks on code availability)

Version 1:

Reviewer comments:

Reviewer #1

(Remarks to the Author)
I appreciate the authors' revisions, which addressed most of my comments, improved the manuscript's clarity, and strengthened the results.

I nonetheless encourage the authors to explicitly cite in the main text all new results and figures introduced in response to reviewers' comments. For example:
-If not yet done, the small-perturbation test results should be included in the Supplementary Notes and referenced in the main manuscript (currently they appear only in the author response).
-Likewise, the new runtime-complexity plot needs a brief mention and contextualization in the main text, rather than existing solely in Supplementary Notes without any citation.

Finally, although the cosine similarity values in Figure 2 are useful, the caption should better explain their meaning (e.g., standard deviations or confidence bounds stand for what?) to help readers interpret them with confidence.

(Remarks on code availability)

Reviewer #2

(Remarks to the Author)
I co-reviewed this manuscript with one of the reviewers who provided the listed reports. This is part of the Nature Communications initiative to facilitate training in peer review and to provide appropriate recognition for Early Career Researchers who co-review manuscripts.

(Remarks on code availability)

Reviewer #3

(Remarks to the Author)
I thank the authors for satisfactorily addressing most of my previous comments. I believe that the revised manuscript now meets the technical standards for publication in Nat Comm. However, there is still one comment that I believe has not been addressed in full: whether the new method proposed here represents a step forward in terms of providing new insights into the domains in which the algorithm is applied. The authors have responded to my questions, but either I didn't explain myself well in my first report, or they didn't understand my concern. In practice, they have shown that the method works well using several networks of different natures (social, political, etc.). However, what they haven't shown is what *new* insights we have learned about the system (social, political, etc.) described by the data. That is, is there something new that this method extracts from the data that we could not get before? Or, on the contrary, does the new method only represent an algorithmic advantage (in some datasets, as this is not always the case) with respect to existing ones? I believe this is a key point that determines whether the MS is a solid technical contribution or one that could also be relevant to get new insights about the systems being studied.

Apart from this remaining question, I believe there is nothing left to be addressed.

(Remarks on code availability)

Reviewer #4

(Remarks to the Author)
The Authors have carefully and comprehensively addressed all comments from the previous review round. The revision substantially strengthens the paper, both in methodological clarity and empirical validation. The added analyses, in particular the convergence and runtime benchmarks, the additional error bars, and the expanded discussion of computational complexity, meaningfully enhance transparency and reproducibility. The improved documentation and inclusion of pseudocode significantly increase the usability of the proposed framework for the broader research community.
I really appreciate information in Figure 1, which now provides convergence and runtime comparisons across datasets, adequately illustrating empirical stability. The results have been improved by appropriately contextualizes the role of mixed membership and possible regularization for sparsity.
The inclusion of cosine-similarity analyses with node attributes effectively addresses the point while avoiding over-interpretation of metadata as ground truth, and also, the manuscript now clearly states that variable-size hyperedges are supported.
The new explanation and Table 2 adequately detail scaling behavior and trade-offs among model variants. Standard-deviation error bars and clarifications on entropy differences have been added, improving robustness of the results. The revised manuscript is now clearer, more complete, and convincingly demonstrates both scalability and interpretability of the proposed framework. The authors’ responses are thorough and technically appropriate, and no substantive issues remain.

(Remarks on code availability)
The Authors have carefully and comprehensively addressed all comments from the previous review round. The revision substantially strengthens the paper, both in methodological clarity and empirical validation. The added analyses, in particular the convergence and runtime benchmarks, the additional error bars, and the expanded discussion of computational complexity, meaningfully enhance transparency and reproducibility. The improved documentation and inclusion of pseudocode significantly increase the usability of the proposed framework for the broader research community.
I really appreciate information in Figure 1, which now provides convergence and runtime comparisons across datasets, adequately illustrating empirical stability. The results have been improved by appropriately contextualizes the role of mixed membership and possible regularization for sparsity.
The inclusion of cosine-similarity analyses with node attributes effectively addresses the point while avoiding over-interpretation of metadata as ground truth, and also, the manuscript now clearly states that variable-size hyperedges are supported.
The new explanation and Table 2 adequately detail scaling behavior and trade-offs among model variants. Standard-deviation error bars and clarifications on entropy differences have been added, improving robustness of the results. The revised manuscript is now clearer, more complete, and convincingly demonstrates both scalability and interpretability of the proposed framework. The authors’ responses are thorough and technically appropriate, and no substantive issues remain.

Version 2:

Reviewer comments:

Reviewer #3

(Remarks to the Author)
I thank the authors for their reply. Unfortunately, I am not convinced by their answer.

While the authors have substantially improved the clarity, scalability analysis, and usability of their framework, I remain

unconvinced that the revised manuscript demonstrates genuinely new structural insights about higher-order data that could not already be obtained using existing models/approaches. The paper effectively unifies and generalizes several known approaches and provides a flexible parameterization spanning assortative to disassortative patterns, but this methodological generality does not translate into clear empirical novelty. Specifically, the manuscript does not present structural discoveries that are uniquely revealed by the proposed model. The case studies (e.g., DAWN, etc) illustrate interpretable patterns, but these appear consistent with what other approaches could already recover. No side-by-side comparisons are shown in which prior methods fail to recover a meaningful structure that the new framework succeeds in identifying. As a result, the claim that the method enables previously inaccessible mesoscale characterization remains unsubstantiated.

In its current form, the contribution is primarily methodological: improved scalability, identifiability, and unification of existing ideas. These are valuable advances, but not sufficient to claim a methodological breakthrough without a clear demonstration that the model leads to new scientific insight from real data.

(Remarks on code availability)

Version 3:

Reviewer comments:

Reviewer #3

(Remarks to the Author)
The authors have addressed the remaining criticism and I believe the paper is now acceptable for publication.

(Remarks on code availability)

Open Access This Peer Review File is licensed under a Creative Commons Attribution 4.0 International License, which permits use, sharing, adaptation, distribution and reproduction in any medium or format, as long as you give appropriate credit to the original author(s) and the source, provide a link to the Creative Commons license, and indicate if changes were made.
In cases where reviewers are anonymous, credit should be given to 'Anonymous Referee' and the source.
The images or other third party material in this Peer Review File are included in the article’s Creative Commons license, unless indicated otherwise in a credit line to the material. If material is not included in the article’s Creative Commons license and your intended use is not permitted by statutory regulation or exceeds the permitted use, you will need to obtain permission directly from the copyright holder.
To view a copy of this license, visit https://creativecommons.org/licenses/by/4.0/

Response to the reviewers

Dear Reviewers,

Thank you for the detailed remarks regarding our Manuscript NCOMMS-25-45134 originally titled “Broad Spectrum Structure Discovery in Large-Scale Higher-Order Networks". The feedback has improved our paper and we hope that the revised version of our work can now be considered suitable for publication in *Nature Communications*.

Here we summarize the main changes to the manuscript with further details given below.

- Throughout the main manuscript we have made revisions and clarifications addressing reviewer comments. The revised text is colored blue.
- In Section IV “Methods" we have added Algorithm 1 which provides pseudocode meant to aid users in fitting the proposed models and configuring the inference algorithms.
- We have added Supplementary Figure 1 and a corresponding paragraph to Supplementary Note 4 to compare model runtimes and performance across the semi- and omni-assortative variants. These provide empirical evidence of the method’s scalability and rapid convergence, while also illustrating the trade-off against performance.
- We have expanded the section "Conclusions" by discussing further the model’s limitations, as suggested by the referees.
- To improve the method’s usability, we have added a Jupyter notebook tutorial to the project’s Github repository illustrating how to use the method. The Jupyter notebook is written in Python. We have also added comments to the previously undocumented Julia scripts and have begun transcribing the code fully into Python, where it will be integrated into the `HypergraphX` package, a popular Python package for modeling and studying hypergraph data.

We provide detailed responses to all reviewers’ comments and suggestions below.

Thank you again for your time and consideration.

Reviewer 1

> *The manuscript "Broad Spectrum Structure Discovery in Large-Scale Higher-Order Networks" by J. Hood and co-authors introduces a versatile probabilistic framework for hypergraphs that captures mesoscale structures, from strictly assortative communities to disassortative class–interaction patterns. By treating latent classes as nodes in an auxiliary hypergraph and imposing a low-rank factorization on class–class affinities, the method remains scalable and yields interpretable node- and class-level structures. Empirical results on pharmacological and social link-prediction benchmarks show clear improvements over existing approaches. Altogether, this is an important contribution to the literature, and I believe it will be of wide interest to researchers using higher-order networks across fields. Overall, the paper is well-written and clear. However, I have some comments and suggestions before recommending it for publication. Please find below a detailed list of my comments:*

Thank you for your careful consideration. We agree—this is an excellent summary of our paper's contributions.

> *The manuscript would benefit from clear guidance on selecting the number of communities and classes. If I understood correctly, it seems that grid search was used throughout the manuscript to obtain specific choices for each dataset. While cross-validation is mentioned, incorporating a possible model-selection criterion or presenting a sensitivity analysis over different values would help practitioners balance under- and over-clustering and make the approach easier to use.*

We agree that selecting the number of communities and classes is not trivial, and offering further guidance, in addition to the paragraph provided in Section II-D, titled "Prediction and model selection" will improve the manuscript. We have now added additional sentences within that paragraph offering such guidance, where we provide problem-dependent recommendations.

> *To ensure the inferred mesoscale structures are robust, it could be worth including a comprehensive stability assessment. For example, report the variability in community assignments and link-prediction metrics across a larger set of random initializations (beyond the ten runs currently noted), and/or conduct a perturbation experiment—such as removing 5–10 % of hyperedges—to confirm that results remain consistent under data changes. These analyses will demonstrate that the detected patterns reflect genuine structure rather than artifacts of particular seeds/noise.*

We agree that the algorithm's sensitivity to small perturbations in the data should be better understood. We followed your suggestion and have run additional experiments on the Supreme Court and hospital datasets, shown in Figure 2. We find that when removing a random 10% of all hyperedges, as suggested, we observe very similar community assignments. We measure the cosine similarity between communities inferred from the whole dataset and from the dataset partially pruned and find the communities align very closely to one another. For each dataset, we observe a mean cosine similarity greater than 0.99 with little variation across seeds (standard deviation less than 0.005). The cosine similarity is measure between 0 and 1, where 1 represents identical membership assignment. We find nearly identical community structure learned from the full and ablated datasets.
More generally, we note that the standard errors of the predictive values over 5 train-test splits as shown

in Fig. 4a are relatively small across datasets, thus we do not expect the communities inferred under edge removal to change substantially.

> *In the manuscript, the authors highlight that the omni-assortative variant is the most expressive (but also the most expensive), while the semi-assortative model strikes a balance and is used in most experiments. To elucidate the trade-offs between expressivity and computational cost, I recommend including a few comparisons of the semi-assortative and omni-assortative models on a selection of synthetic/real-world datasets. Such side-by-side benchmarks would clarify each variant's relative performance, runtime, and suitability for different applications.*

This is a great point. While we provide a discussion of the runtime complexity for each method in Section IV “Methods" and a summary in Table 2, we agree that it is important to empirically demonstrate differences in runtime and relative performance. We have added Supplementary Figure 1 to Supplementary Note 4, where we compare runtime against the evidence lower bound (ELBO) achieved by each model variant.

> *The description of critical implementation choices—such as EM initialization strategies, convergence thresholds, and stopping criteria—is currently spread across the Methods and Supplementary sections, which makes reproduction challenging. I recommend adding a concise pseudocode box or flowchart in the main Methods that clearly outlines each inference step, lists key hyperparameters, and specifies used criteria for convergence and termination.*

This is a great idea, thank you for your suggestion. We have added pseudocode to the “Methods" section in Algorithm 1 that clearly lists the key hyperparameters, outputs, the inner training algorithm and convergence criteria. We also include the inference algorithms specific to each model variant in Supplementary Note 2.

> *In addition to the previous comment, it is crucial that the authors provide a well-documented code on Github. The current repository contains unannotated Julia scripts with no usage examples, making them difficult to use. I strongly recommend releasing a well-commented version of the code that clearly exposes each model variant, includes example notebooks on synthetic and real datasets, and perhaps reproduces core figures from the manuscript. This will greatly enhance reproducibility and adoption.*

We also really appreciated this point and have worked to substantially improve the code's usability. We have added a Jupyter notebook tutorial to the project's Github repository which illustrates how to use the method and how to interpret and visualize the model parameters. The Jupyter Notebook is written in Python and shows how to replicate plots from Figure 2b. We have also commented the Julia code, as requested. Moreover, we are currently developing a Python implementation in parallel for integratation into `HypergraphX` Lotito et al. (2023), a popular library for modeling and studying hypergraph data; we have added this to the repository as well.

> *While the current benchmarks on pharmacological and social datasets are compelling, including an additional domain—such as co-authorship hypergraphs—would further demonstrate the method's*

> *versatility and broaden its appeal.*

We agree that applications to additional domains would further demonstrate the method's versatility and broaden its appeal. We feel the paper is already quite long and includes several different examples; however, we have added further discussion in Section D "Datasets" of various other domains that this method could be applied to. Moreover, we are currently beginning to collaborate with a domain specialist on an in-depth case study on patent citation hypergraph data as follow-up work, which we believe will provide further demonstration of the method.

> *Minor comments:*

> *Ensure all figure panels and supplementary notes are numbered in the order they appear.*

We have re-ordered the Supplementary Notes to match the order they appear and ensured the each Figure is numbered in the order it appears.

> *In Figure 5, choose more distinguishable color palettes and include error bars (or clarify deterministic vs. stochastic behavior) for the omni-assortative results.*

We have changed the color palettes to a blue/bronze contrast and included error bars, reflecting variability across 5 randomly generated datasets.

> *It's not clear whether the statement "The omniassortative model achieves lower H() values for five of the six datasets (senate-bills, senate-committees, NDC-substances, DAWN, and workplace) while for contact-high-school, the values are similar." is supported by any hypothesis tests or significance (e.g., error bars in some cases should not support the claim)*

We have clarified for which datasets the standard errors overlap, and added prose indicating where there are substantial differences.

Reviewer 2

> *I co-reviewed this manuscript with one of the reviewers who provided the listed reports. This is part of the Nature Communications initiative to facilitate training in peer review and to provide appropriate recognition for Early Career Researchers who co-review manuscripts.*

Thank you for your help and assistance in reviewing. We appreciate the time and energy you put into the review.

Reviewer 3

> *The paper introduces a family of probabilistic generative models aimed at discovering and interpreting mesoscale structures in large-scale hypergraphs. The authors use a framework that jointly clusters nodes into latent classes and classes into communities, using low-rank tensor factorizations to ensure scalability and interpretability. They applied the framework to study some real-world datasets. I believe that the study is technically correct and, although it uses known techniques, their combination produces novel results. Also, the problem that is investigated is worth looking at. Therefore, I believe this contribution has the potential to be considered for publication in Nat Comm. However, it is not acceptable in its present form for the reasons listed below (in no particular order):*

Thank you for the feedback. We hope that the revision has addressed your comments.

> *I believe that the generality of the current framework is not discussed enough. The authors certainly present "success cases", but I don't see a way to prove that their method is practical for large datasets or that the use of low-rank factorizations and variational inference allows for tractable estimation on high-order, large-scale hypergraphs. More details on these or a deeper discussion would improve the potential impact and general appeal of the study.*

We agree that conveying and characterizing the model's generality is an important priority. While the model is "low-rank", our method scales only linearly in K, and arbitrarily complex structure can be modeled as K grows large. Moreover, the EM algorithm is provably guaranteed to converge to a local maximum of the log-likelihood, regardless of the size of the dataset. We have provided a discussion of computational complexity in Section IV "Methods", with a summary in Table 2, where we show that it is linear in either the number of nodes or the number of observed hyperedges (whichever is larger); critically, our method does not scale with the number of *possible* hyperedges, only the observed ones, which in practice, is often a vanishing fraction of the total possible. This is very efficient, considering that several existing and popular inference methods for networks scale quadratically in the number of nodes. If the data is prohibitively large for running the proposed algorithm, our algorithm may be modified to handle mini-batch updates, which would be useful to develop and implement in future work. The DAWN data represents an illustrative case study, where the data consists of thousands of nodes and hundreds of thousands of hyperedges, ranging from order 2 to 16. Multiple datasets in our study consist of high-order interactions beyond order 5 and contain thousands of hyperedges. We have also added a plot comparing runtime to log-likelihood for each dataset, and hope that this further strengthens the generality of the current framework.

> *The validation using real-world data is somewhat limited: While the datasets used are diverse, more domain-specific insights would strengthen claims of interpretability.*

While space in the paper is limited, we do currently include experiments on political, social, judicial, and pharmacological datasets, which we believe is already fairly diverse. However, we have further included a list of additional domains in Section D where the method could be applicable. We also note that the different experiments reported in the article are meant to convey different important aspects of the model. The empirical results in Section D are meant to convey the method's predictive performance and its interpretability while the in-depth case study on the DAWN dataset in Section C is meant to provide a more grounded illustration of the model's ability to surface scientifically meaningful latent structure. In the future, we intend to build on this work by using the proposed model to analyze co-authorship and patent citation hypergraph data, which we hope will provide yet more diversity to this model's applications.

> *Please, provide more evidence regarding statements of scalability. In the current version, not enough quantitative indicators of performance are provided. Otherwise, some statements should be significantly toned down.*

We agree that the scalability of our method should be better understood. We thoroughly discuss computational complexity under the Section IV "Methods". A summary of the computational complexity of each variant may be found in Table 2. We have also added Supplementary Figure 1, which compares the runtime and log-likelihood values across datasets and model variants to further provide empirical statistics regarding scalability. Supplementary Figure 1 includes metrics for the DAWN data, the largest of the datasets we study, which demonstrates the advantage of the semi-assortative model over the omni-assortative model in this setting.

> *The paper is hard to follow in several parts as the notation is dense. It could benefit from a "running example" to ground the concepts. Also, for the sake of reaching a wider audience, a bit more of (even if trivial) explanations regarding the use of multiple indices and latent variables (e.g., nodes, classes, communities) could be helpful to avoid confusion of readers unfamiliar with tensor-based models. I particularly like the approach, but the subset of network scientists used to work with tensorial representations is limited, and so could be the audience if the text is not simplified a bit or explained in more detail. For instance, a figure with a model diagram and a step-by-step illustration of the generative process could help.*

We agree that it is crucial for the work to reach a wider audience. We recognize that tensor representations can be difficult to digest, and have taken several actions to make our model more accessible. We iterated several times before finally deciding on the current notation, optimizing for a minimal set of indices that does not compromise mathematical rigor and precision. The model is itself non-trivial and we believe the current notation provides a good tradeoff between rigor and clarity. However, in addition to Figure 1 and Figure 2 which are meant to aid intuition, we have added Algorithm 1 to Section IV "Methods" which clearly outlines the set of key inputs, training process, and outputs to the training algorithm. The Justice and hospital datasets are meant to serve as illustrative examples, and the case study in Section IIC is meant to ground the concepts further. We have also added a tutorial to the Github, which is meant to illustrate how the model may be used and interpreted. Finally, we are implementing a pure

Python implementation of the code to be integrated into `HypergraphX` Lotito et al. (2023), a popular Python library for modeling and studying hypergraph data. We hope that these various measures make our model more accessible.

> *The assumption of low-rank structure in class interactions might be unrealistic for certain hypergraphs with high local complexity. Please, add a discussion of the limitations or alternative extensions for more complex hypergraph topologies.*

While our model significantly extends the range of possible mesoscopic structures that can be captured, we agree that this assumption might fail in more complex scenarios that do not match our chosen model family. Following your insight, we have added a paragraph describing the limitations to the conclusion. There, we point to alternative extensions for complex hypergraph topologies, as well as extensions for modeling different data types and structures, such as continuous or binary hypergraph data.

> *Once the above comments and suggestions have been thoroughly addressed, the paper could be reconsidered for publication.*

Thanks you for your constructive and thorough review, which we greatly appreciate. Your suggestions have substantially improved the quality of the paper. We hope we have thoroughly addressed your comments.

Reviewer 4

> *This paper introduces a principled and scalable framework for uncovering mesoscale structure in large-scale hypergraphs, networks capturing polyadic interactions. The Authors propose a probabilistic model that combines latent node classes with low-rank tensor decomposition to extract interpretable patterns of interaction. The framework generalizes classical stochastic block models to higher-order data, enables structure discovery beyond traditional graph-based techniques, and is applicable to a broad range of domains, including social networks, tagging systems, and pharmacological interactions. The model is evaluated on several large, real-world datasets and consistently outperforms state-of-the-art baselines on link prediction tasks while also offering highly interpretable structural insights. In general the manuscript is clearly written, well-organized, and supported by informative and well-labeled figures. The introduction of latent-class modeling for hypergraphs fills a notable gap in the field. The approach bridges the strengths of the stochastic block modeling approaches with modern tensor techniques. The Authors provide a variational expectation maximization algorithm capable of handling datasets with millions of nodes and hyperedges, making the method suitable for large-scale applications. Beyond prediction, the proposed model gives meaningful structural insights via latent hypergraphs and class-role visualizations. A thorough evaluation on multiple datasets, two biological datasets, three human-contact networks, and three political datasets, showcases strong performance in link prediction and interpretable structure discovery.*

Thank you for your careful consideration and analysis. We agree—this is an excellent summary of our paper's contributions.

> *In the following some comments and suggestions:*

> *1. While theoretical identifiability is discussed, practical convergence behavior and empirical stability of the variational expectation maximization algorithm are not deeply addressed. Including convergence plots or runtime benchmarks would strengthen this section.*

This is a great point—other reviewers have made similar comments regarding convergence and runtime, and we agree that the empirical stability of our method should be better understood. We have included Supplementary Figure 1 which compares log-likelihoods and runtime across models to serve as runtime benchmarks for datasets of varying size.

> *2. The manuscript could benefit from ablation experiments comparing hard vs. soft class assignments or evaluating the role of multi-membership in predictive accuracy.*

We agree that it is important to understand how hard versus soft class assignments affect a model's scope, limitations, and results. Previous work by Contisciani et al. (2022) compares hard—e.g., the all-or-nothing model by Chodrow et al. (2021)—to soft class assignment structures. They find that in general mixed-membership improves predictive performance. We also note that existing hard membership models for hypergraphs, like the variants proposed by Chodrow et al. (2021), are either restricted to express only assortative structure or are not scalable and can be run only on small datasets; as a result, it is challenging to perform a fair comparison to these methods.

Our model is a mixed-membership model. Ideally, one could devise ways for any of these models to select between hard and soft adaptively based on the data and some performance criteria. This is however challenging, as it would imply setting complex constraints on the parameters. Alternatively, one could impose a regularization term to the cost function to encourage sparsity. This is something that can be easily incorporated into our model via a suitable prior and then using maximum a posteriori estimates, as we discuss in the Supplementary Note 2. This additional term would be independent from the specific choices behind our model. We have now added a sentence in Supplementary Note 3 to clearly state this connection between prior, MAP and regularization for sparsity. Finally, we note that in principle, our model allows for hard solutions to be inferred, as they are particular cases of a mixed-membership where the learned membership vectors have only one non-zero entry. We find empirically that the omni-assortative model tends to learn sparser parameters than a strictly-assortative model, in several cases finding hard memberships, as shown in Figure 2.

> *3. In addition to link prediction, metrics such as modularity, normalized mutual information, or adjusted rand index could be used to assess how well the learned classes align with known structures (when available).*

We understand the point of making use of extra information when available, so we report cosine similarities with attributes in Figure 2b to illustrate how learned communities align with attributes, as you have suggested. We are hesitant in making conclusions about the model performance based on node attributes. This is tricky, given the absence of ground truth and the possible pitfalls of considering node attributes as a proxy for it, as thoroughly discussed in Peel et al. (2017).

> *4. Clarify whether hyperedge sizes are assumed to be uniform or variable.*

We have added two lines clarifying this in the first full paragraph of page 4.

> *5. A brief discussion of computational complexity, especially for large-rank tensors, would be helpful.*

We provide details on computational complexity in Table 2 and discuss the tradeoff between models in subsection A of the Section IV "Methods". The runtime scales linearly in the rank of the factorization (K).

> *6. Reporting standard deviations or confidence intervals in performance metrics would improve result robustness.*

We completely agree that error bars are important for quantifying and assessing uncertainty. In addition to the plots that had standard errors for error bars, we have now added error bars representing standard deviations to Figure 5 and have clarified which differences in entropy are more substantial, as their error bars suggest, in Figure 4b.

> *The manuscript presents a novel, well-executed, and broadly applicable framework for structure discovery in higher-order networks. The methodological rigor, scalability, and interpretability of the approach—coupled with convincing empirical results—make this paper a valuable contribution for network science communities.*

Thank you again for your careful and thoughtful evaluation of our work.

References

Chodrow, P. S., Veldt, N., and Benson, A. R. (2021). Generative hypergraph clustering: From blockmodels to modularity. *Science Advances*, 7(28):eabh1303.

Contisciani, M., Safdari, H., and De Bacco, C. (2022). Community detection and reciprocity in networks by jointly modelling pairs of edges. *Journal of Complex Networks*, 10(4):cnac034.

Lotito, Q. F., Contisciani, M., De Bacco, C., Di Gaetano, L., Gallo, L., Montresor, A., Musciotto, F., Ruggeri, N., and Battiston, F. (2023). Hypergraphx: a library for higher-order network analysis. *Journal of Complex Networks*, 11(3):cnad019.

Peel, L., Larremore, D. B., and Clauset, A. (2017). The ground truth about metadata and community detection in networks. *Science advances*, 3(5):e1602548.

Response to the reviewers

Dear Reviewers,

Thank you for the additional, detailed remarks regarding our Manuscript NCOMMS-25-45134 originally titled “Broad Spectrum Structure Discovery in Large-Scale Higher-Order Networks". The feedback has further improved our paper and we hope that the revised version of our work can now be considered suitable for publication in *Nature Communications*.

We provide detailed responses to the reviewers’ remaining comments and suggestions below.

Thank you again for your time and consideration.

Reviewer 1

> *I appreciate the authors’ revisions, which addressed most of my comments, improved the manuscript’s clarity, and strengthened the results.*

We are glad that you find the manuscript stronger and clearer.

> *I nonetheless encourage the authors to explicitly cite in the main text all new results and figures introduced in response to reviewers’ comments. For example: -If not yet done, the small-perturbation test results should be included in the Supplementary Notes and referenced in the main manuscript (currently they appear only in the author response). -Likewise, the new runtime-complexity plot needs a brief mention and contextualization in the main text, rather than existing solely in Supplementary Notes without any citation.*

Thank you for suggesting this. We have now added a paragraph in “Supplementary Note 4: Experimental details” discussing sensitivity of community assignments to small perturbations and referenced it inside the main in section D “Applicability across domains: prediction, interpretability, and heterogeneity”. We have then added prose to the section "Methods: computational complexity" of the main text, which mentions and contextualizes the runtime-complexity plot and the trade-off with performance.

> *Finally, although the cosine similarity values in Figure 2 are useful, the caption should better explain their meaning (e.g., standard deviations or confidence bounds stand for what?) to help readers interpret them with confidence.*

We have added additional context and prose in the caption.

Reviewer 2

> *I co-reviewed this manuscript with one of the reviewers who provided the listed reports. This is part of the Nature Communications initiative to facilitate training in peer review and to provide appropriate recognition for Early Career Researchers who co-review manuscripts.*

Again, thank you for your help and assistance in reviewing. We appreciate the time and energy you put into the review.

Reviewer 3

> *I thank the authors for satisfactorily addressing most of my previous comments. I believe that the revised manuscript now meets the technical standards for publication in Nat Comm. However, there is still one comment that I believe has not been addressed in full: whether the new method proposed here represents a step forward in terms of providing new insights into the domains in which the algorithm is applied. The authors have responded to my questions, but either I didn't explain myself well in my first report, or they didn't understand my concern. In practice, they have shown that the method works well using several networks of different natures (social, political, etc.). However, what they haven't shown is what *new* insights we have learned about the system (social, political, etc.) described by the data. That is, is there something new that this method extracts from the data that we could not get before? Or, on the contrary, does the new method only represent an algorithmic advantage (in some datasets, as this is not always the case) with respect to existing ones? I believe this is a key point that determines whether the MS is a solid technical contribution or one that could also be relevant to get new insights about the systems being studied.*

Thank you raising this, we agree that this is a key point indeed. We do believe our model is extracting a new kind of structure from the data, which previous approaches cannot extract, and which is qualitatively more informative about the underlying system. More specifically, while a substantial portion of the paper is indeed devoted to technical and algorithmic advances, those advances then enable these kinds of models to discover a full spectrum of assortative to disassortative structure in large-scale hypergraphs.

Applying this approach, we then do find novel substantive insights. In particular, when applying this approach to large-scale pharmacological data (i.e., the DAWN dataset), our model discovers disassortative classes of drugs. Drugs in such classes are often combined by ER patients with drugs in other classes, but rarely combined with other drugs in the same class. We find that such classes correspond to drugs with similar pharmacological function (e.g., heart pressure drugs) that patients typically only take one of. Previous modeling approaches which assume strictly assortative structure for computational convenience cannot discover such structure, and the drug classes inferred by such models thus conflate co-occurrence with co-function. This distinction is particularly important for generating good hypotheses about dangerous drug combinations. While the combination of *any one* heart pressure drug and *any one* recreational drug may cause dangerous complications, a model which cannot represent disassortativity may instead suggest the false insight that combinations of multiple heart pressure drugs are dangerous.

We have updated the manuscript to emphasize both the particular insight in this case study, as well as the more general fact that our technical advances permit the discovery of a fundamentally new kinds of insights about the complex systems underlying large-scale hypergraph data.

> *Apart from this remaining question, I believe there is nothing left to be addressed.*

Thank you for your detailed feedback and insights, which have substantially improved the manuscript.

Reviewer 4

> *The Authors have carefully and comprehensively addressed all comments from the previous review round. The revision substantially strengthens the paper, both in methodological clarity and empirical validation. The added analyses, in particular the convergence and runtime benchmarks, the additional error bars, and the expanded discussion of computational complexity, meaningfully enhance transparency and reproducibility. The improved documentation and inclusion of pseudocode significantly increase the usability of the proposed framework for the broader research community. I really appreciate information in Figure 1, which now provides convergence and runtime comparisons across datasets, adequately illustrating empirical stability. The results have been improved by appropriately contextualizes the role of mixed membership and possible regularization for sparsity. The inclusion of cosine-similarity analyses with node attributes effectively addresses the point while avoiding over-interpretation of metadata as ground truth, and also, the manuscript now clearly states that variable-size hyperedges are supported. The new explanation and Table 2 adequately detail scaling behavior and trade-offs among model variants. Standard-deviation error bars and clarifications on entropy differences have been added, improving robustness of the results. The revised manuscript is now clearer, more complete, and convincingly demonstrates both scalability and interpretability of the proposed framework. The authors' responses are thorough and technically appropriate, and no substantive issues remain.*

Thank you for your detailed feedback. We are glad the manuscript is now clearer, more complete, and overall much improved.

Response to the reviewers

Dear Reviewers,

Thank you for the additional, detailed remarks regarding our Manuscript NCOMMS-25-45134 originally titled “Broad Spectrum Structure Discovery in Large-Scale Higher-Order Networks". The feedback has further improved our paper and we hope that the revised version of our work can now be considered suitable for publication in *Nature Communications*.

We provide a detailed response to Reviewer 3’s remaining comments and suggestions below. Specifically, we have now provided a side-by-side comparison (shown in Fig. 3) where we show that prior methods fail to recover meaningful structure from pharmacological data that our model successfully identifies.

Thank you again for your time and consideration.

Reviewer 3

> *The paper effectively unifies and generalizes several known approaches and provides a flexible parameterization spanning assortative to disassortative patterns, but this methodological generality does not translate into clear empirical novelty. Specifically, the manuscript does not present structural discoveries that are uniquely revealed by the proposed model. The case studies (e.g., DAWN, etc) illustrate interpretable patterns, but these appear consistent with what other approaches could already recover. No side-by-side comparisons are shown in which prior methods fail to recover a meaningful structure that the new framework succeeds in identifying. As a result, the claim that the method enables previously inaccessible mesoscale characterization remains unsubstantiated. In its current form, the contribution is primarily methodological: improved scalability, identifiability, and unification of existing ideas. These are valuable advances, but not sufficient to claim a methodological breakthrough without a clear demonstration that the model leads to new scientific insight from real data.*

We are grateful for your response, as it motivated us to perform additional side-by-side comparisons that have strengthened our manuscript. Specifically, we compared the results from the DAWN case study to those obtained by the method of Contisciani et al. (2022), a state-of-the-art method that we compare to elsewhere in the paper and which is representative of existing baselines. We have added these side-by-side comparisons to Figure 3 of the main paper and added several paragraphs to the corresponding discussion, highlighting the structural differences between the methods. In particular, we detail how our method discovers disassortative structure which existing methods cannot recover. Moreover, we discuss how when the baseline does recover similar communities to our method, the communities are less interpretable, since the model cannot express how such communities interact disassortatively. We further discuss how in this particular setting, this restriction prevents the baseline method from identifying possible combinations of complementary medications. Again, we believe these empirical comparisons significantly strengthen the manuscript by substantiating the theoretical claim that existing baselines cannot adequately measure disassortative structure, and we thank the reviewer for suggesting them.

References

Contisciani, M., Battiston, F., and De Bacco, C. (2022). Inference of hyperedges and overlapping communities in hypergraphs. *Nature Communications*, 13(1):7229.

Response to the reviewers

Reviewer 3

> *The authors have addressed the remaining criticism and I believe the paper is now acceptable for publication.*

Thank you for the positive feedback and for recommending our paper for publication. Thank you again for your time and consideration.
